# Exoskeletal-Assisted Walking in Veterans With Paralysis: A Randomized Clinical Trial

**DOI:** 10.1001/jamanetworkopen.2024.31501

**Published:** 2024-09-04

**Authors:** Ann M. Spungen, Ellen J. Dematt, Kousick Biswas, Karen M. Jones, Zhibao Mi, Amanda J. Snodgrass, Kel Morin, Pierre K. Asselin, Christopher M. Cirnigliaro, Steven Kirshblum, Peter H. Gorman, Lance L. Goetz, Katherine Stenson, Kevin T. White, Alice Hon, Sunil Sabharwal, B. Jenny Kiratli, Doug Ota, Bridget Bennett, Joseph E. Berman, Denis Castillo, Kenneth K. Lee, Byron W. Eddy, M. Kristi Henzel, Michelle Trbovich, Sally A. Holmes, Felicia Skelton, Michael Priebe, Stephen L. Kornfeld, Grant D. Huang, William A. Bauman

**Affiliations:** 1 Spinal Cord Damage Research Center, James J. Peters Veterans Affairs (VA) Medical Center, Bronx, New York; 2 Departments of Rehabilitation and Human Performance and Medicine, Icahn School of Medicine at Mount Sinai, New York, New York; 3 VA Cooperative Studies Program Coordinating Center, VA Maryland Health Care System, Perry Point; 4 Department of Epidemiology and Public Health, Division of Biostatistics School of Medicine, University of Maryland, Baltimore; 5 Now retired.; 6 VA Cooperative Studies Program Clinical Research Pharmacy Coordinating Center, Albuquerque, New Mexico; 7 University of New Mexico, College of Pharmacy, Albuquerque; 8 VA Providence Healthcare System, Providence, Rhode Island; 9 Department of Physical Medicine and Rehabilitation, Rutgers New Jersey Medical School, Newark; 10 Kessler Institute for Rehabilitation and The Kessler Foundation, West Orange, New Jersey; 11 Department of Neurology, University of Maryland School of Medicine, Baltimore; 12 Richmond VA Medical Center, Richmond, Virginia; 13 Department of Physical Medicine and Rehabilitation, Virginia Commonwealth University School of Medicine, Richmond; 14 VA St Louis Health Care System–Jefferson Barracks, St Louis, Missouri; 15 Departments of Orthopaedics and Neurology, Division of Physical Medicine and Rehabilitation, Washington University School of Medicine, St Louis, Missouri; 16 James A. Haley Veterans’ Hospital, Tampa, Florida; 17 Department of Physical Medicine and Rehabilitation, University of South Florida, Tampa; 18 VA Long Beach Health Care System, Long Beach, California; 19 Department of Physical Medicine and Rehabilitation, University of California Irvine; 20 VA Boston Health Care System, Boston, Massachusetts; 21 Department of Physical Medicine and Rehabilitation, Harvard Medical School, Boston, Massachusetts; 22 VA Palo Alto Health Care System, Palo Alto, California; 23 Department of Orthopaedic Surgery, Stanford University School of Medicine, Stanford, California; 24 VA North Texas Health Care System, Dallas; 25 Department of Physical Medicine and Rehabilitation, The University of Texas Southwestern Medical Center, Dallas; 26 Clement J. Zablocki VA Medical Center, Milwaukee, Wisconsin; 27 Department of Physical Medicine and Rehabilitation, Medical College of Wisconsin, Milwaukee; 28 Minneapolis VA Health Care System, Minneapolis, Minnesota; 29 Louis Stokes Cleveland VA Medical Center, Cleveland, Ohio; 30 Department of Physical Medicine & Rehabilitation, Case Western Reserve School of Medicine, Cleveland, Ohio; 31 South Texas Veterans Health Care System–Audie Murphy Division, San Antonio; 32 Department of Rehabilitation Medicine, University of Texas Health Science Center, San Antonio; 33 Michael E. DeBakey VA Medical Center, Houston, Texas; 34 Department of Physical Medicine and Rehabilitation, Baylor College of Medicine, Houston, Texas; 35 Charlie Norwood VA Medical Center, VA Augusta Health Care System, Augusta, Georgia; 36 Spinal Cord Injury/Disorders Service, James J. Peters VA Medical Center, Bronx, New York; 37 VA Cooperative Studies Program Central Office, VA Office of Research and Development, Washington, DC

## Abstract

**Importance:**

Robotic exoskeletons leverage technology that assists people with spinal cord injury (SCI) to walk. The efficacy of home and community exoskeletal use has not been studied in a randomized clinical trial (RCT).

**Objective:**

To examine whether use of a wheelchair plus an exoskeleton compared with use of only a wheelchair led to clinically meaningful net improvements in patient-reported outcomes for mental and physical health.

**Design, Setting, and Participants:**

This RCT of veterans with SCI was conducted at 15 Veterans Affairs medical centers in the US from September 6, 2016, to September 27, 2021. Data analysis was performed from March 10, 2022, to June 20, 2024.

**Interventions:**

Participants were randomized (1:1) to standard of care (SOC) wheelchair use or SOC plus at-will use of a US Food and Drug Administration (FDA)–cleared exoskeletal-assisted walking (EAW) device for 4 months in the home and community.

**Main Outcomes and Measures:**

Two primary outcomes were studied: 4.0-point or greater improvement in the mental component summary score on the Veterans RAND 36-Item Health Survey (MCS/VR-36) and 10% improvement in the total T score of the Spinal Cord Injury–Quality of Life (SCI-QOL) physical and medical health domain and reported as the proportion who achieved clinically meaningful changes. The primary outcomes were measured at baseline, post randomization after advanced EAW training sessions, and at 2 months and 4 months (primary end point) in the intervention period. Device usage, reasons for not using, and adverse events were collected.

**Results:**

A total of 161 veterans with SCI were randomized to the EAW (n = 78) or SOC (n = 83) group; 151 (94%) were male, the median age was 47 (IQR, 35-56) years, and median time since SCI was 7.3 (IQR, 0.5 to 46.5) years. The difference in proportion of successes between the EAW and SOC groups on the MCS/VR-36 (12 of 78 [15.4%] vs 14 of 83 [16.9%]; relative risk, 0.91; 95% CI, 0.45-1.85) and SCI-QOL physical and medical health domain (10 of 78 [12.8%] vs 11 of 83 [13.3%]; relative risk, 0.97; 95% CI, 0.44-2.15) was not statistically different. Device use was lower than expected (mean [SD] distance, 1.53 [0.02] miles per month), primarily due to the FDA-mandated companion being unavailable 43.9% of the time (177 of 403 instances). Two EAW-related foot fractures and 9 unrelated fractures (mostly during wheelchair transfers) were reported.

**Conclusions and Relevance:**

In this RCT of veterans with SCI, the lack of improved outcomes with EAW device use may have been related to the relatively low device usage. Solutions for companion requirements and user-friendly technological adaptations should be considered for improved personal use of these devices.

**Trial Registration:**

ClinicalTrials.gov Identifier: NCT02658656

## Key Points

**Question:**

Does use of a wheelchair plus an exoskeleton for 4 months in the home and community environments compared with use of a wheelchair only improve patient-reported mental and physical health outcomes for veterans with spinal cord injury?

**Findings:**

In this randomized clinical trial of 161 veterans with paralysis, no significant differences were found in mental or physical health for those using a wheelchair plus an exoskeleton vs those using a wheelchair only. The exoskeleton group reported low use of the device.

**Meaning:**

Personal use of an exoskeleton in the home and community did not result in improved patient-reported outcomes in veterans with paralysis; safe device modifications to reduce companion requirements are needed to increase personal use.

## Teaser

This randomized clinical trial examines whether the use of an exoskeletal-assisted walking device improves patient-reported mental and physical health outcomes among veterans with paralysis.

## Introduction

Paralysis from spinal cord injury (SCI) is a catastrophic condition with diminishing gains in functional recovery over time, often with severe walking limitations and some degree of permanent disability.^1,2^ The Veterans Health Administration (VHA) cares for the single largest population of people with SCI in the US. Approximately 17 000 veterans with SCI are seen annually in 1 of 25 spinal cord injuries and disorders (SCI/D) centers (eFigure 1 in Supplement 1).^3^

Historically, strategies for walking after SCI have included use of leg bracing^4^ or functional electrical stimulation with crutches or a walker,^5^ requiring high energy consumption and minimal long-term user adoption.^5,6,7,8,9,10^ Robotic or manual body weight–supported treadmill training offers a form of assisted-walking but is not performed over ground.^11,12,13,14^ The first-generation exoskeletal devices potentially provide an energy-efficient solution to support over-ground walking by using computer algorithm–controlled external leg bracing with motors at the hip and knees along with use of assistive devices for balance.

Improvements in outcomes after 2 to 4 months of in-hospital, supervised exoskeletal-assisted walking (EAW) were reported for body composition,^15,16^ energy expenditure,^16,17^ and bowel function.^18,19^ The safety and efficacy of EAW in the home and community have not been prospectively studied. We performed a randomized clinical trial (RCT) to examine the efficacy of supervised training sessions followed by 4 months of EAW in the home and community environments in veterans with SCI on clinically meaningful net improvements in patient-reported outcomes for mental and physical health and an array of exploratory outcome measures.

## Methods

### Trial Design

A parallel, 2-group RCT was conducted at 15 VHA SCI/D centers (eFigure 1 in Supplement 1). Eligible participants were randomly assigned within site (1:1 allocation) using an interactive touch-tone randomization system generated by the coordinating center.^20^ Participant and trainer blinding were not possible due to the visibility of exoskeleton use. However, outcome assessors at each site were blinded. The study chairpersons were blinded to enrollment without access to participants or results until study closure to data collection. The study was reviewed and approved by the VHA Central Institutional Review Board. This report follows the Consolidated Standards of Reporting Trials (CONSORT) reporting guidelines for RCTs.^21^ Study design details may be found in the full trial protocol (Supplement 2) and a prior publication.^20^

### Participants and Companions

This study was conducted between September 6, 2016, and September 27, 2021. During this time, 424 veterans with SCI were screened, 210 were excluded, 53 declined, and 161 consented to participate and were randomized (Figure; eTable 3 in Supplement 1). Twenty-eight were treated as early terminators due to the COVID-19 pandemic (Figure).

**Figure.**
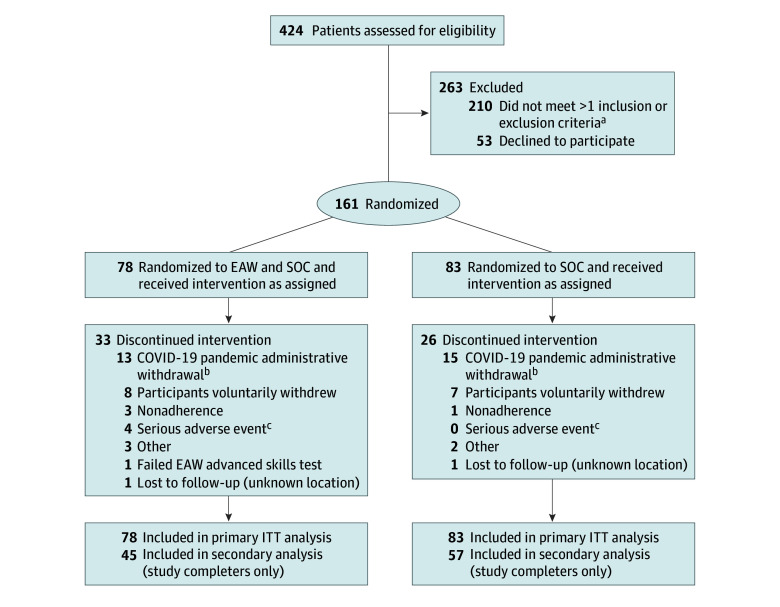
Participant Flow Diagram EAW indicates exoskeletal-assisted walking; ITT, intention to treat; and SOC, standard of care control group. Note that the EAW group is labeled “EAW and SOC” but is referred to in analyses as the EAW group. ^a^The major reasons for 1 or more screening failures were bone mineral density loss (n = 64), range of motion or spasticity (n = 39), medical contraindications (n = 31), failed EAW basic skills test (n = 32), anthropometric or weight limits (n = 27), fracture history (n = 21), level of spinal cord injury or neurologic status (n = 21), no companion available (n = 15), and other (n = 9). ^b^The COVID-19 early administrative withdrawals were participants who were actively enrolled in the study when the pandemic necessitated a study hold and were eventually terminated by the data monitoring committee due to an inability to restart the study. ^c^The 4 serious adverse events in the EAW group that resulted in early withdrawals were 2 fractures, including 1 femur fracture that occurred during transfer from wheelchair to toilet (not related) and 1 foot stress fracture (related) diagnosed as a probable preexisting stress fracture reoccurring during EAW; the other 2 serious adverse events were due to substance abuse and a medical issue, both of which were not study related. There were no adverse event–related early terminations in either group.

Veterans or active-duty soldiers 18 years or older with SCI of more than 6 months and who used a wheelchair for mobility were recruited. Key exclusion criteria included weight greater than 100 kg, history of lower-extremity fracture(s), extremely low hip (less than −3.5 total hip T score) or knee bone mineral density (BMD) (proximal tibia or distal femur BMD<0.60 g/cm^2^),^22^ severe spasms or contractures, and medical or health conditions for which walking was contraindicated. Participants were asked to self-report their race and ethnicity identity for diversity inclusion in accordance with Veterans Affairs research policy. A complete list of eligibility criteria is provided (eTables 1 and 2 in Supplement 1).^20^

The US Food and Drug Administration (FDA) requires a trained companion to accompany the personal exoskeletal device user. The site team determined whether each companion was physically able to assist the participant with the required tasks. Each participant had up to 3 companions who trained with them during the EAW training sessions. Veteran participants and companions provided separate written informed consents for screening and randomization.

### Procedures and Intervention

The study consisted of screening, randomization, orientation for the standard of care (SOC) group and advanced exoskeletal in-hospital training for the EAW group (phase 1), and 4 months of intervention (phase 2). The SOC group was offered poststudy training sessions in the exoskeleton; no outcome data were collected. Adverse events were recorded for all study components.

To ensure members of both groups were eligible and able to use an exoskeleton, all participants were fitted for the device, received 5 preliminary EAW training sessions, and were required to pass the EAW basic skills test before randomization.^20^ The SOC group received orientation by attending weekly meetings and/or were contacted to review their usual activities as an attention balance (while the EAW group was being trained on the exoskeleton). The EAW group received 30 or fewer sessions of advanced training and were required to pass the advanced skills test^20^ with their companion(s) before taking the exoskeleton home. During the home and community intervention, the EAW group continued with usual activities using their wheelchair but were also provided with a ReWalk 6.0 powered exoskeleton (ReWalk Robotics Inc) for at-will use. The SOC group continued with usual activities. Both groups received weekly contact from the research coordinators to review their weekly log forms.

### Primary and Major Secondary Outcomes

On the basis of preliminary data, 2 primary outcome measurements were chosen: (1) mental component summary (questions 4, 5, 8, and 9) of the Veterans RAND 36-Item Health Survey (MCS/VR-36), for which a 4.0-point or greater improvement was considered to indicate greater vitality, social functioning, and improved role-emotional and mental health^23,24^; and (2) the summary T score of the Spinal Cord Injury–Quality of Life (SCI-QOL) physical and medical health domain for bladder management difficulties, bladder complications, bowel management difficulties, and pain interference item banks, for which a 10% improvement was considered a clinically relevant change.^25^ The major secondary outcome was a 1.0-kg or greater total body fat mass loss (TBFmass).^26,27^ These outcome measures were analyzed as the proportion of group participants who did or did not achieve the clinically meaningful change from baseline to month 4.

### Secondary Outcomes

All secondary outcomes were intended to be exploratory and included 6 patient-reported outcome surveys: (1) Global Impression of Change Scale for Severity and Improvement, completed by participants and companions; (2) Patient-Reported Outcomes Measurement Information System sleep disturbance scale^28^; (3) SCI Functional Index^29^; (4) SCI-QOL emotional health domain^30,31^; (5) SCI-QOL social participation domain^32^; and (6) self-reported measures of bowel function. Objective secondary outcomes included change in visceral adipose tissue,^27^ high-density lipoprotein cholesterol,^33,34^ low-density lipoprotein cholesterol,^35,36^ triglycerides, total cholesterol, fasting plasma glucose,^36,37,38^ fasting plasma insulin,^36,37,38^ and calculation of Homeostasis Model Assessment for Insulin Resistance.^39^

All outcomes were assessed at baseline and 3 time points after randomization: orientation and training, 2 months, and 4 months (primary outcome time point). For the EAW group, number of steps from the exoskeleton step log, amount of time the device was used, locations of use, and reasons why the device was not used were recorded on a weekly log form. Usual activities were recorded weekly for both groups using a fixed-answer format.

### Sample Size

Sample size estimation and power analysis were based on hypothesis testing of the 2 primary and major secondary outcomes for change from baseline to 4 months. Using hypothesized expected outcomes, we hypothesized that 33% of the intervention group and 10% of the control group would achieve a 4.0-point or greater change on the MCS/VR-36; 42% and 10%, respectively, were hypothesized to have a 10% improvement on the summary T score in the combined SCI-QOL physical and medical health domain; and 35% and 10%, respectively, were hypothesized to maintain a 1.0-kg or greater TBFmass loss. Two-tailed superiority tests for the primary and major secondary end points with a sample size of 160 individuals (80 per group) had 80% power at a *P* < .025 significance level, assuming an attrition rate of 15%. In March 2019, an interim analysis was performed from 86 evaluable participants and found none of the primary or major secondary end points met the study stopping criteria (eMethods in Supplement 1).

### Statistical Analysis

The primary end point was change from baseline to month 4, and an intention-to-treat (ITT) analysis was performed, including all randomized participants. By design, there were no missing cases: participants either met the outcome criteria (success), did not meet these criteria (failure), or terminated early (failure). For the 2 primary and the major secondary outcomes, the proportion of successes was compared between the EAW and SOC groups using Pearson χ^2^ tests, constituting the primary and secondary efficacy analyses. Secondary analysis included only participants who completed the study (excluding early terminators) and was performed the same as the primary analysis. Additional secondary analyses used all data available and compared mean and mean difference scores from baseline to each time point, using 2-tailed, unpaired *t* tests. Wilcoxon tests were also performed for variables for which normality assumption was violated. Repeated-measures analyses were completed using generalized linear mixed models. Exploratory post hoc analyses were conducted to determine relationships between the number of steps taken in the exoskeleton with all outcomes. All statistical tests were 2-sided. The 2 primary and major secondary outcomes were tested at a *P* < .0125 level of significance. Due to the number of secondary outcomes, all were tested at a significance level of *P* < .01 to reduce type 1 error. SAS software, version 9.4 (SAS Institute Inc) was used to conduct all analyses. Data analysis was performed from March 10, 2022 to June 20, 2024.

## Results

### Study Participants

A total of 161 veterans with SCI were randomized to EAW (n = 78) or SOC (n = 83), of whom 151 (94%) were male and 10 (6%) were female. The median age was 47 (IQR, 35-56) years, and median time since injury was 7.3 (IQR, 0.5-46.5) years. Two participants (1.2%) were American Indian or Alaska Native, 1 (0.6%) Asian, 27 (16.8%) Black, 109 (67.7%) White, 19 (11.8%) of other race (including multiple or unspecified), and 3 (1.9%) of unknown or unreported race (Table 1).

**Table 1.** Demographic and Clinical Characteristics of the Participants by Group at Baseline^a^

Characteristic	EAW group (n = 78)	SOC group (n = 83)
Age, median (IQR), y	47 (34-56)	46 (36-56)
Height, mean (SD), cm	176.5 (6.1)	177.0 (7.1)
Weight, median (IQR), kg	82.1 (70.3-90.7)	80.3 (72.6-90.3)
BMI, mean (SD)	25.8 (3.7)	25.7 (3.7)
Total body fat mass, mean (SD), kg	27.3 (9.1)	27.0 (8.6)
Visceral fat mass, median (IQR), kg	1.60 (0.69-2.39)	1.50 (0.67-2.23)
Duration of SCI, median (IQR), y	7.4 (0.5-44.1)	6.3 (0.5-46.5)
Sex		
Male	72 (92.3)	79 (95.2)
Female	6 (7.7)	4 (4.8)
Ethnicity		
Hispanic or Latino	8 (10.3)	19 (22.9)
Not Hispanic or Latino	66 (84.6)	63 (75.9)
Unknown or not given	4 (5.1)	1 (1.2)
Race		
Asian	0	1 (1.2)
Black or African American	14 (17.9)	13 (15.7)
American Indian or Alaska Native	2 (2.6)	0
White	55 (70.5)	54 (65.1)
Other^b^	6 (7.7)	13 (15.7)
Unknown or not given	1 (1.3)	2 (2.4)
Cause of SCI		
Traumatic	66 (84.6)	74 (89.2)
Nontraumatic	12 (15.4)	9 (10.8)
Paraplegia	68 (87.2)	71 (85.5)
Complete motor	45 (66.2)	57 (80.3)
Incomplete motor	23 (33.8)	14 (19.7)
Tetraplegia	10 (12.8)	12 (14.5)
Complete motor	4 (40.0)	6 (50.0)
Incomplete motor	6 (60.0)	6 (50.0)

Abbreviations: BMI, body mass index (calculated as weight in kilograms divided by height in meters squared); EAW, exoskeletal-assisted walking; SCI, spinal cord injury; SOC, standard of care.

^a^ Data are presented as number (percentage) of participants unless otherwise indicated.

^b^ This category included participants who identified with multiple races (n = 8) and those who selected “other” but did not specify race (n = 11).

### Primary and Major Secondary Outcome Results

All 161 randomized participants were included in the ITT analysis. The proportions of successes were not statistically different between the groups for the MCS/VR-36 (EAW: 12 of 78 [15.4%]; SOC: 14 of 83 [16.9%]; relative risk [RR], 0.91; 95% CI, 0.45-1.85; *P* = .80); SCI-QOL physical and medical health domain (EAW: 10 of 78 [12.8%]; SOC: 11 of 83 [13.3%]; RR, 0.97; 95% CI, 0.44-2.15; *P* = .93), and TBFmass loss (EAW: 14 of 78 [17.9%]; SOC: 16 of 83 [19.3%]; RR 0.93, 95% CI, 0.49 to 1.78; *P* = .83) (Table 2). Secondary analysis of study completers (45 in the EAW group and 57 in the SOC group) demonstrated a similar percentage of successes in both groups as the primary analyses but failed to reach significance (MCS/VR-36: 12 of 45 [26.7%] vs 14 of 57 [24.6%]; *P* = .81; SCI-QOL physical and medical health: 10 of 45 [22.2%] vs 11 of 57 [19.3%]; *P* = .72); and TBFmass loss: 14 of 45 [31.1%] vs 16 of 57 [28.1%]; *P* = .74).

**Table 2.** Results of the Primary and Major Secondary Outcome Measures by Time Point and Group (Primary Analysis, All Participants)^a^

Outcome	EAW group (n = 78)	SOC group (n = 83)	RR (95% CI)	*P* value
Mental component summary of the Veterans RAND 36-Item Health Survey^b^				
Baseline score, median (IQR)	65.4 (58.4 to 67.3) (n = 78)	63.4 (53.9 to 67.2) (n = 82)		.34
Training and orientation	66.5 (59.8 to 68.6) (n = 54)	62.4 (55.8 to 66.1) (n = 68)		.01
2 Months (intervention)	65.3 (58.0 to 68.3) (n = 48)	63.7 (49.6 to 67.6) (n = 60)		.17
4 Months (intervention)	65.4 (60.2 to 69.1) (n = 45)	62.1 (48.1 to 67.8) (n = 57)		.15
Change from baseline by time point				
Training and orientation	1.3 (−2.2 to 6.1) (n = 54)	−1.2 (−4.7 to 3.4) (n = 67)		.08
2 Months (intervention)	1.1 (−2.4 to 4.3) (n = 48)	0.3 (−4.3 to 3.8) (n = 59)		.36
4 Months (intervention)	0.0 (−4.8 to 4.3) (n = 45)	0.8 (−6.7 to 3.9) (n = 56)		.88
Primary outcome (≥4-point improvement), No. (%)				
Success	12 (15.4)	14 (16.9)	0.91 (0.45 to 1.85)	.80
Failure	66 (84.6)	69 (83.1)		
SCI-QOL physical medical health domain^c^				
Baseline score, median (IQR)	152 (137 to 167) (n = 78)	149 (135 to 170) (n = 83)		.86
Training and orientation	146 (137 to 162) (n = 54)	153 (134 to 173) (n = 67)		.61
2 Months (intervention)	147 (130 to 161) (n = 47)	145 (134 to 169) (n = 58)		.41
4 Months (intervention)	146 (133 to 163) (n = 43)	147 (129 to 170) (N = 57)		.58
Change from baseline by time point				
Training and orientation	−3.2 (−13.4 to 5.9) (n = 54)	0.0 (−11.6 to 1.9) (n = 67)		.19
2 Months (intervention)	−1.3 (−12.4 to 3.0) [47]	−1.4 (−12.9 to 9.4) (n = 58)		.26
4 Months (intervention)	−4.1 (−17.2 to 3.3) (n = 43)	−1.0 (−1.5 to 12.0) (n = 57)		.16
Primary outcome (10% improvement in summary T score), No. (%)				
Success	10 (12.8)	11 (13.3)	0.97 (0.44 to 2.15)	.94
Failure	68 (87.2)	72 (86.7)		
Total body fat mass^d^				
Baseline, mean (SD), kg	27.3 (9.1) (n = 78)	27.0 (8.6) (n = 83)		.86
Training and orientation	28.4 (9.5) (n = 54)	27.4 (8.7) (n = 65)		.57
2 Months (intervention)	28.5 (9.6) (n = 47)	27.0 (8.5) (n = 57)		.41
4 Months (intervention)	28.5 (9.7) (n = 45)	26.5 (7.7) (n = 55)		.26
Change from baseline by time point, median (IQR)				
Training and orientation	0.4 (−0.9 to 1.2) (n = 54)	0.2 (−0.8 to 1.3) (n = 65)		.84
2 Months (intervention)	0.6 (−1.1 to 2.2) (n = 47)	0.0 (−1.3 to 1.2) (n = 57)		.45
4 Months (intervention)	0.4 (−1.7 to 1.9) (n = 45)	−0.2 (−1.2 to 1.9) (n = 55)		.79
Major secondary outcome (≥1.0 kg loss), No. (%)				
Success	14 (17.9)	16 (19.3)	0.93 (0.49 to 1.78)	.83
Failure	64 (82.1)	67 (80.7)		

Abbreviations: EAW, exoskeletal-assisted walking; RR, relative risk; SCI-QOL, Spinal Cord Injury–Quality of Life; SOC, standard of care.

^a^ The 2 primary and the major secondary outcome measurements were based on the percentage of individuals in each group who achieved the described specific criteria (success) for each variable at 4 months after randomization. Continuous data with normal distribution are presented as mean (SD) using unpaired, 2-tailed *t* tests, and nonnormally distributed data are presented as median (IQR) using Wilcoxon tests.

^b^ Mental component summary of the Veterans RAND 36-Item Health Survey for vitality, social functioning, role-emotional, and mental health.^22,23^

^c^ Summary T score for the SCI-QOL physical, medical, and health domain for 4 of the item banks: bladder management difficulties, bladder complications, bowel management difficulties, and pain interference.^24^

^d^ Total body fat mass was determined by dual energy x-ray absorptiometry scanning.^25,26^

### Secondary Outcomes

The participants’ Global Impression of Severity of SCI at baseline was not statistically different between the 2 groups (Table 3). The EAW participants and companions reported significant global impression of improvement at each time point compared with the SOC group (Table 3). The EAW group compared with the SOC group reported significant reduction in sleep disturbance at training and orientation (median change from baseline T score, −1.9 [IQR, −5.7 to 3.0] vs 1.3 [IQR, −3.2 to 7.0]); *P* = .01) and 2 months (median change from baseline T score, −2.7 [IQR, −7.0 to 2.2] vs 1.8 (IQR, −3.2 to 5.7); *P* = .01) but not at 4 months. No other significant differences between the groups were noted for change in SCI Functional Index, SCI-QOL emotional health domain, and SCI-QOL social participation domain (Table 3). Self-reported measures of bowel function (eTable 4 in Supplement 1) and change in visceral adipose tissue, high-density lipoprotein cholesterol, low-density lipoprotein cholesterol, triglycerides, total cholesterol, fasting plasma glucose, fasting plasma insulin, and Homeostasis Model Assessment for Insulin Resistance (eTable 5 in Supplement 1) were not statistically different between the groups at any time point.

**Table 3.** Results of the Secondary Outcome Measures by Time Point and Group^a^

Outcome	EAW group	SOC group	*P* value
Global impression of severity of SCI			
Participant’s impression			
Baseline score, median (IQR)	2 (2 to 3) (n = 78)	2 (2 to 3) (n = 82)	.74
Training and orientation	2 (1 to 3) (n = 54)	3 (2 to 3) (n = 67)	.01
2 Months (intervention)	2 (2 to 3) (n = 47)	3 (2 to 3) (n = 59)	.15
4 Months (intervention)	2 (1.5 to 3) (n = 44)	3 (2 to 4) (n = 57)	.07
Change from baseline score			
Training and orientation	0 (−1 to 0) (n = 54)	0 (0 to 1) (n = 66)	.01
2 Months (intervention)	0 (−1 to 1) (n = 47)	0 (0 to 1) (n = 58)	.25
4 Months (intervention)	0 (−0.5 to 0) (n = 44)	0 (0 to 1) (n = 56)	.06
Companion’s impression			
Baseline score, median (IQR)	2 (2 to 3) (n = 77)	2 (2 to 3) (n = 81)	.93
Training and orientation	2 (2 to 3) (n = 51)	2 (1 to 3) (n = 64)	.34
2 Months (intervention)	2 (2 to 3) (n = 45)	2 (2 to 3) (n = 56)	.63
4 Months (intervention)	2 (1 to 3) [41]	2 (2 to 4) [51]	.92
Change from baseline score			
Training and orientation	0 (−1 to 1) (n = 51)	0 (−1 to 1) (n = 63)	.99
2 Months (intervention)	0 (−1 to 1) (n = 45)	0 (−1 to 1 (n = 56)	.92
4 Months (intervention)	0 (0 to 1) (n = 41)	0 (0 to 1) (n = 51)	.89
Global impression of improvement of SCI			
Participant’s change from baseline score, median (IQR)			
Training and orientation	5 (3 to 6) (n = 54)	2 (1 to 3) (n = 69)	<.001^b^
2 Months (intervention phase)	4 (3 to 6) (n = 47)	2 (1 to 5) (n = 58)	<.001^b^
4 Months (postrandomization)	5 (3 to 6) (n = 43)	3 (2 to 4) (n = 55)	<.001^b^
Companion’s change from baseline score, median (IQR)			
Training and orientation	5 (3 to 6) (n = 51)	2.5 (1 to 4.5) (n = 64)	<.001^b^
2 Months (intervention)	5 (3 to 6) (n = 45)	3 (2 to 4) (n = 55)	<.001^b^
4 Months (intervention)	5 (4 to 6) (n = 40)	3 (1 to 5) (51)	.01^b^
PROMIS sleep disturbance			
Baseline T score, median (IQR)	46 (38 to 53) (n = 78)	46 (38 to 54) (83)	.83
Training and orientation	44 (38 to 54) (n = 54)	50 (41 to 55) (68)	.20
2 Months (intervention)	46 (38 to 55) (n = 48)	49 (40 to 54) (59)	.92
4 Months (intervention)	47 (40 to 58) (n = 45)	48 (41 to 56) (57)	.94
Change from baseline T score, median (IQR)			
Training and orientation	−1.9 (−5.7 to 3.0) (n = 54)	1.3 (−3.2 to 7.0) (68)	.01
2 Months (intervention)	−2.7 (−7.0 to 2.2) (n = 48)	1.8 (−3.2 to 5.7) (59)	.01
4 Months (intervention)	0.0 (−4.8 to 4.0) (n = 45)	1.5 (−4.1 to 5.3) (57)	.50
SCI-FI			
Baseline summary T score, mean (SD)	344 (24.50) (n = 29)	343 (20.6) (n = 31)	.92
Training and orientation	345 (23.72) (n = 20)	338 (17.6) (n = 24)	.24
2 Months (intervention)	348 (24.92) (n = 18)	335 (20.6) (n = 24)	.09
4 Months (intervention)	348 (16.53) (n = 22)	339 (24.0) (n = 22)	.17
Change from baseline T score, mean (SD)			
Training and orientation	5.4 (20.97) (n = 18)	−5.8 (11.63) (n = 19)	.06
2 Months (intervention)	7.3 (16.00) (n = 16)	−4.5 (12.99) (n = 19)	.02
4 Months (intervention)	3.8 (16.36) (n = 17)	−8.6 (20.60) (n = 17)	.06
Emotional health			
Negative constraints			
Baseline summary T score, median (IQR)	219 (200 to 255) (n = 78)	226 (201 to 257) (83)	.60
Training and orientation	220 (199 to 245) (n = 54)	226 (195 to 272) (68)	.28
2 Months (intervention)	208 (194 to 233) (n = 48)	219 (188 to 273) (60)	.35
4 Months (intervention)	207 (191 to 226) (n = 44)	223 (193 to 263) (57)	.10
Change from baseline summary T score, median (IQR)			
Training and orientation	−5.3 (−19.0 to 7.1) (n = 54)	1.1 (−10.3 to 14.2) (n = 68)	.06
2 mo (intervention)	−6.4 (−21.3 to 6.5) (n = 48)	−0.7 (−10.6 to 14.0) (n = 60)	.08
4 mo (intervention)	−8.7 (−21.3 to 4.0) (n = 44)	−3.0 (−13.0 to 10.6) (n = 57)	.06
Positive aspects			
Baseline score, median (IQR)	173 (159 to 189) (n = 78)	167 (150 to 192) (n = 82)	.70
Training and orientation	173 (155 to 187) (n = 54)	160 (144 to 199) (n = 68)	.70
2 Months (intervention)	178 (154 to 192) (n = 48)	165 (141 to 191) (n = 60)	.30
4 Months (intervention)	176 (160 to 192) (n = 45)	162 (141 to 189) (n = 57)	.19
Change from baseline score, median (IQR)			
Training and orientation	0.0 (−7.9 to 7.6) (n = 54)	0.0 (−11.3 to 5.9) (n = 67)	.50
2 Months (intervention)	0.2 (−2.9 to 11.6) (n = 48)	0.0 (−10.6 to 6.7) (n = 59)	.21
4 Months (intervention)	2.4 (−2.7 to 12.7) (n = 45)	0.0 (−10.0 to 7.1) (n = 56)	.12
Social participation			
Baseline summary T score, median (IQR)	155 (141 to 169) (n = 77)	155 (143 to 178) (n = 83)	.33
Training and orientation	154 (145 to 169) (n = 53)	153 (141 to 177) (n = 68)	.87
2 Months (intervention)	149 (142 to 173) (n = 47)	155 (137 to 179) (n = 59)	.98
4 Months (intervention)	158 (145 to 178) (n = 44)	152 (139 to 176) (n = 57)	.37
Change from baseline summary T score, median (IQR)			
Training and orientation	2.4 (−7.8 to 10.5) (n = 52)	0.0 (−8.3 to 4.8) (n = 68)	.12
2 Months (intervention)	0.0 (−7.5 to 9.5) (n = 46)	−0.2 (−7.7 to 4.4) (n = 59)	.23
4 Months (intervention)	4.8 (−4.3 to 14.5) (n = 43)	0.0 (−13.4 to 6.3) (n = 57)	.04

Abbreviations: EAW, exoskeletal-assisted walking; PROMIS, Patient-Reported Outcomes Measurement Information System (short form); SCI, spinal cord injury; SCI-FI, Spinal Cord Injury–Functional Index; SOC, stand of care.

^a^ Continuous data with normal distribution are presented as mean (SD) using unpaired, 2-tailed *t* tests, and nonnormally distributed data are presented as median (IQR) using Wilcoxon tests.

^b^ Significant results (*P* ≤ .001) are in favor of the EAW group. Any other nonsignificant results (*P* ≤ .01) are in favor of the EAW group at those time points.

### Exoskeletal Device Use and Usual Activities

The EAW group reported using the exoskeletal device a mean (SD) of 86 (46) minutes per week (range, 0-248 minutes per week) for 7.7 (5.3) weeks (range, 0-16 weeks). The mean (SD) cumulative total step count ranged from 4321 (4654) to 6192 (10 707) steps per month (range across all EAW participants, 250-57 766 steps per month), which is a mean (SD) distance of 1.53 (0.02) miles per month (range, 0.07-16.40 miles per month). The EAW group reported walking in the exoskeleton on tile, wood, carpet, and smooth indoor surfaces and concrete, asphalt, cement, dirt, gravel, and cobblestone outdoor surfaces; in their homes; on sidewalks; and in shopping centers, restaurants, places of worship, and parks (eTable 6 in Supplement 1). Among 403 reported instances, the predominant reason for not using the exoskeleton was companion unavailability (177 [43.9%]). Other nonuse reasons were illness (70 [17.4%]), being busy (58 [14.4%]), travel (37 [9.2%]), and inclement weather (24 [6.0%]) (eTable 6 in Supplement 1). No significant relationships were found between the number of EAW steps and any of the outcome measures.

The mean (SD) times of participation in usual activities were similar between the EAW (830 [666] minutes per week) and SOC (937 [754] minutes per week) groups (eTable 7 in Supplement 1). Usual activities included household chores, pushing a wheelchair for exercise, stationary arm cycling, weightlifting, stretching, wheelchair sports, and some non–wheelchair-based activities (eTable 7 in Supplement 1).

### Safety and Adverse Events

During screening, 34 serious adverse events (SAEs) occurred in 27 participants; 145 adverse events (AEs) occurred in 87 participants (eTable 8 in Supplement 1). During the postrandomization period, 18 SAEs and 157 AEs occurred in the EAW group and 28 SAEs and 165 AEs in the SOC group. Twelve of 78 participants (15.4%) in the EAW group and 14 of 83 (16.9%) in SOC group experienced 1 or more SAEs (eTable 8 in Supplement 1). Overall, 11 fractures occurred, including 9 non–study-related and 2 exoskeleton-related fractures (eTable 8 in Supplement 1). The 2 study-related fractures occurred during initial standing and stepping (no fall or trauma) in the first year of enrollment. Because both cases were possibly undiagnosed preexisting calcaneus fractures from the radiology report, bilateral foot radiographs were added to screening with no other foot or other device-related fractures occurring (eResults in Supplement 1). To determine the number of exoskeletal-related SAEs and AEs, we reported on the whole study group during screening and separately by randomized group (eTable 8 in Supplement 1).

## Discussion

In this trial, the use of an exoskeleton in the home and community of veterans with chronic SCI did not result in significantly greater proportions of clinically meaningful changes in mental as well as physical and medical health outcomes compared with the control group who used only their wheelchairs. Likewise, there was no significant difference between the groups for TBFmass loss. Secondary analysis of study completers also failed to reach statistical significance between groups. Because both groups had a small proportion of improvement in the primary outcomes, the positive changes may have been attributed to participation in a clinical trial. The lack of difference in the primary and major secondary outcomes are speculated to be explained by the relatively low device usage: a mean of 86 minutes per week for a total mean walking distance of 1.53 miles per month. This low amount of walking may have been insufficient to elicit a change and is considerably less than the approximately 5000 steps per day reported in ambulatory sedentary populations.^40^ Van Dijsseldonk et al^41^ surveyed 14 motor-complete SCI users of the ReWalk exoskeleton in their homes and communities and reported similar findings of low use but good satisfaction with use for social events and exercise. A previous report of home- and community-based use of other ambulatory devices in patients with SCI also showed poor long-term use.^42^ The low amount of device use in this trial is in contrast to the institutional-based RCT in which EAW was performed under supervision for 3 to 5 hours per week with the mean (SD) steps being much higher at 1420 (491) per session (approximately 17 000 steps per month).^43^ In that study and others with supervised training, favorable changes in body composition, bowel function, energy expenditure, and quality of life were noted.^15,17,18,19,44^ Among our participants, EAW demonstrated extreme nonuse and use for any given month (0.07-16.40 miles).

After training in the exoskeleton and at 2 and 4 months of personal use, veterans with SCI reported a significant impression of global improvement in their SCI, suggesting that even with minimal use of an exoskeleton, or possibly from being in the study intervention arm, there was an associated positive change in their global impression of SCI. These findings are similar to those of van Dijsseldonk et al,^41^ who also reported low use but good satisfaction. However, use of the exoskeleton in the home and community was not associated with statistically significant improvements in functional independence, emotional health, and social participation.

Potential exoskeletal device–related safety concerns included falls resulting in a fracture or other injury and fragility fractures from weightbearing while standing and stepping in those with extremely low BMD. Reduction of long-bone fracture risk was addressed with strict hip and knee BMD exclusion criteria.^22^ No exoskeleton-related long bone fractures occurred during this study. Two device-related calcaneus fractures occurred with initial standing in the device, which were likely preexisting. Nine fractures occurred that were related to wheelchair falls, transfers, or other nonexoskeletal device activities.

### Future Considerations and Lessons Learned

Research for a cure for SCI has made little progress in helping patients recover the ability to walk, and few other interventions have made over-ground walking safe and feasible in this population. Powered exoskeletons are assistive devices and not a cure for SCI. To increase usability, exoskeletal devices require further improvements, such as easier donning and doffing, increased walking speed, hands-free or self-balancing technology, lateral and backward stepping for better maneuverability, stair climbing, and, most importantly, enhancement of safety features that reduce or eliminate the need for a companion. Although the EAW group only walked a little more than a 1.5 miles per month on average, that is an amount of walking they could not have accomplished without the exoskeleton. Although a few small studies have shown that EAW sessions in a supervised hospital setting have demonstrated health benefits in participants with SCI,^15,18^ device innovations are needed to improve usability in the home and community.

Lessons from this study include the need to estimate (a priori) high screen failure and withdrawal rates due to the daily challenges faced by people living with paralysis. Clinicians treating patients with SCI and individuals with SCI should be made aware of the importance of maintaining bone health and adequate range of motion in the lower extremities for eligibility to participate in future walking strategies.

### Limitations

The high number of screen failures and withdrawals and the low amount of device use were limitations to this study. Of the veterans with chronic SCI who wished to participate, more than half did not meet the BMD and fracture history and/or range of motion criteria at screening. Although it is widely known that people with SCI lose significant BMD,^22,45,46,47,48^ the high percentage of screen failures due to significant bone loss and/or fracture history was unexpected. A major contributor to the withdrawal rate was the interruption to the study by the COVID-19 pandemic. The most influential factor associated with low exoskeleton use was the unavailability of companion(s). Additionally, the study was underpowered because when the sample size was calculated it did not account for any predefined interim analyses. Despite these limitations, no other technology to date has offered over-ground walking on this scale to people with SCI. With appropriate screening and training, using an exoskeleton in the home and community can be safely accomplished. The importance of this technology is evidenced by the decisions of the VHA and Centers for Medicare & Medicaid Services to provide FDA-cleared exoskeletal devices to eligible veterans and nonveterans with SCI.^49,50^

## Conclusions

Given current health care trends of identifying positive quality-of-life outcomes in many populations,^51,52,53,54^ home and community use of this first-generation personal exoskeleton in the SCI population failed to support improved quality of life. The VHA Cooperative Studies Program and SCI/D centers provided an appropriate infrastructure to conduct this RCT. Safety-focused eligibility criteria were implemented successfully to minimize AEs. Lessons learned from this trial may be implemented for people with SCI in future protocols of more advanced exoskeletal devices and other walking strategies, such as spinal stimulation for people with SCI.

## Notes

Supplement 1. **eMethods.** Supplemental Methods **eResults.** Supplemental Results **eFigure.** Geographic Locations of the Veterans Health Administration Spinal Cord Injury/Disorders (SCI/D) System of Care and the Participating Sites **eTable 1.** Participant and Companion Eligibility Criteria **eTable 2.** Fracture Definition for Eligibility Criteria **eTable 3.** Reasons for Screen Failures and Study Withdrawals **eTable 4.** Results of Self-reported Bowel Function for Each Time Point Assessment and Group **eTable 5.** Results of Visceral Adipose Tissue Mass, Lipid Profile, and HOMA-IR for Each Time Point Assessment and Group **eTable 6.** Location, Surface, and Step Count for Exoskeletal Device Usage and Reasons for Not Using the Device **eTable 7.** Self-Reported Record of Usual Weekly Activities During the Intervention Phase by Group **eTable 8.** Serious Adverse and Adverse Events During Screening and Post Randomization

Supplement 2.
Trial Protocol and Statistical Analysis Plan

Supplement 3.
Data Sharing Statement
