# Supplementary material for: Exoskeletal-Assisted Walking in Veterans With Paralysis: A Randomized Clinical Trial
Source: JAMA Netw Open. 2024 Sep 4;7(9):e2431501. doi: 10.1001/jamanetworkopen.2024.31501 (PMC11375472; doi:10.1001/jamanetworkopen.2024.31501)
Supplement: Supplement 1. — eMethods. Supplemental Methods eResults. Supplemental Results eFigure. Geographic Locations of the Veterans Health Administration Spinal Cord Injury/Disorders (SCI/D) System of Care and the Participating Sites eTable 1. Participant and Companion Eligibility Criteria eTable 2. Fracture Definition for Eligibility Criteria eTable 3. Reasons for Screen Failures and Study Withdrawals eTable 4. Results of Self-reported Bowel Function for Each Time Point Assessment and Group eTable 5. Results of Visceral Adipose Tissue Mass, Lipid Profile, and HOMA-IR for Each Time Point Assessment and Group eTable 6. Location, Surface, and Step Count for Exoskeletal Device Usage and Reasons for Not Using the Device eTable 7. Self-Reported Record of Usual Weekly Activities During the Intervention Phase by Group eTable 8. Serious Adverse and Adverse Events During Screening and Post Randomization [file jamanetwopen-e2431501-s001.pdf]

Supplementary Online Content

Spungen AM, Dematt EJ, Biswas K, et al. Exoskeletal-assisted walking in veterans with paralysis: a randomized clinical trial. *JAMA Netw Open*. 2024;7(9):e2428372. doi:10.1001/jamanetworkopen.2024.28372

eMethods. Supplemental Methods
eResults. Supplemental Results
eFigure. Geographic locations of the Veterans Health Administration Spinal Cord Injury/Disorders (SCI/D) System of Care and the Participating Sites
eTable 1. Participant and Companion Eligibility Criteria
eTable 2. Fracture Definition for Eligibility Criteria
eTable 3. Reasons for Screen Failures and Study Withdrawals
eTable 4. Results of Self-reported Bowel Function for each Time Point Assessment and Group
eTable 5. Results of Visceral Adipose Tissue Mass, Lipid Profile, and HOMA-IR for Each Time Point Assessment and Group
eTable 6. Location, Surface, and Step Count for Exoskeletal Device Usage and Reasons for Not Using the Device
eTable 7. Self-Reported Record of Usual Weekly Activities During the Intervention Phase by Group
eTable 8. Serious Adverse and Adverse Events During Screening and Post Randomization

This supplementary material has been provided by the authors to give readers additional information about their work.

© 2024 Spungen AM et al. *JAMA Network Open.*

eMethods. Supplemental Methods

Supplemental eBackground

Veterans with traumatic and non-traumatic spinal cord injuries and disorders (SCI/D) are provided specialty health care across the Veterans Health Administration (VHA) Spinal Cord Injury and Disorders (SCI/D) Systems of Care, which includes 25 SCI/D Centers, or Hubs, and 122 affiliated SCI/D Spoke Sites. As of December 1, 2023, 16,734 living Veterans with SCI/D are identified in the VHA SCI/D Registry (VHA SCIDR), VHA Service Support Center (VSSC) platform, which is an informatics phenotype / algorithm.[1] Veterans identified in the VHA SCIDR VSSC platform meet diagnostic and SCI/D specialty care utilization criteria from October 1, 2012 to present. The VHA **SCI/D Registry** is available for aggregate, current numbers.[1] During the years of enrollment for this study, about 17,000 Veterans with SCI were registered in the VHA medical system in one of the 25 SCI/D Centers and/or their Spokes. The SCI/D Centers are uniquely poised to conduct multi-site clinical trials, which are otherwise difficult to design and implement outside such an expansive and integrated healthcare system (eFigure 1).

Supplemental eStudy Development and Oversight

The RCT was designed with extensive consideration by the study Chairpersons (James J. Peters VA Medical Center, Bronx, NY 10468), Executive Planning Committee (which consisted of subject matter experts in SCI medicine and rehabilitation, clinical trial design, patient-reported outcomes, engineering, robotics, powered exoskeletons, and statistics), and members of the Perry Point Coordinating Center, Cooperative Studies Program, Office of Research and Development, Veterans Health Administration, Department of Veterans Affairs (Perry Point, Maryland 21902) and the Clinical Research Pharmacy Coordinating Center, Cooperative Studies Program, Office of Research & Development, Veterans Health Administration, Department of Veterans Affairs, (Albuquerque, NM 87106), which also manages device studies and tracks adverse events.

The study was conducted in compliance with regulations as specified by Good Clinical Practice (GCP) guidelines. The local site investigators were assisted with GCP compliance by the CSP Site Monitoring, Auditing and Resource Team.[2] Adverse events and results were collected and reported to the sponsor (CSP) and the associated committees. Intervention and data collection integrity was ensured by standardized training of site personnel in EAW fitting/training, administration of patient-reported outcome measurements, and study procedures.

Supplemental eMethods:

Influence of the COVID-19 pandemic

The first participant was consented on September 6, 2016. By mid-March of 2020, 161 participants had been randomized with 28 actively enrolled in various protocol phases when the COVID-19 pandemic became widespread in the US. On March 16, 2020, a safety decision was made by study leadership to place CSP #2003 on administrative hold, ceasing any in-person visits until further notice, as were most other VA-sponsored clinical research studies at that time in the US. On September 20, 2020, a closed session of the Data Monitoring Committee meeting was held regarding the status of the 28 remaining participants. Due to the unpredictability imposed by the pandemic and complications involved to re-start the active participants because of the nature of the ITT study design, the following decisions were made:

- Closure of the study at the participating sites (at the end of the budgeted timeline – September 30, 2021).
- Immediate release (termination) of the remaining 28 active participants from the study.

© 2024 Spungen AM et al. *JAMA Network Open.*

- Randomization not to be re-opened following the administrative hold.

To honor of the integrity of an ITT RCT, the 28 participants were counted as withdrawals (study failures) and are reported as such in the primary results from this study.

Dual photon x-ray absorptiometry (DXA) scanning

The methods for measuring bone mineral density (BMD) at the total hip, distal femur and proximal tibia (knee)[3,4] and for measuring total body fat mass[4] and visceral adiposity tissue (VAT)[5] have been previously described.

Interim Analysis

An interim analysis was performed for the two primary endpoints and the major secondary endpoint in March 2019 when 80 participants had evaluable data as planed in the protocol. Actual data was from 86 evaluable participants (42 in EAW and 44 in SOC). None of the analysis results of these endpoints fell within the acceptance or rejection regions defined by two-sided O'Brien-Fleming boundaries. The Data Monitoring Committee (DMC) recommended continuing the study to recruit remaining participants towards the targeted sample size. The detailed methods are presented in Supplement 1.

Inclusion and Exclusion Eligibility Criteria (eTable 1 and eTable 2)

Primary and Major Secondary Outcome Measurements

Mental Component Summary of the Veterans Rand-36 (MCS/VR-36) (questions 4, 5, 8, and 9). MCS-VR-36 is a 12-item self-reported instrument used to assess: a. Vitality; b. Social Functioning; c. Role-Emotional; and d. Mental Health. For each item, participants selected a response to indicate how frequent problems arose by choosing one of the following: 'none of the time', 'a little of the time', 'some of the time', 'most of the time', or 'all of the time'. A clinically relevant change was established by a committee of SCI experts to be a ≥4.0 point improvement in the MCS/VR-36, demonstrating greater vitality and social functioning and improved role-emotional and mental health.[6]

Spinal Cord Injury-Quality of Life Physical-Medical-Health (SCI-QOLPMH) domain (sum T-score) for bladder management difficulties, bladder complications, bowel management difficulties, and pain interference item banks. The SCI-QOL PMH domain is a 3-item bank tool used to assess bladder management difficulties, bladder complications, bowel management difficulties, pain interference, and pain behavior. For each item, participants select a response to indicate the characteristics of bladder, bowel, and pain challenges 'lately' or "in the past 7 days". 'Lately' is participant self-defined. Responses are provided using a 5-item scale – 'not at all/never,' 'a little bit/rarely,' 'somewhat/sometimes,' quite a bit/often,' or 'very much/always'. A 10% improvement on the sum T-score of the SCI-QOL PMH was considered a clinically relevant change by a committee of SCI experts using the nominal group method.[6]

Total body fat mass was assessed by a total body scan using a dual energy-ray absorptiometry scanner (Lunar iDXA, GE Healthcare, Madison, WI).[4,5] The major secondary outcome measure was ≥1.0 kg total body fat mass loss.

Secondary Exploratory Outcome Descriptions

The Global Impression of Change Scale was completed by participants and companions. The companion's responses acted as a proxy validation of the participant's self-assessment. The impression of severity of spinal cord injury was

© 2024 Spungen AM et al. *JAMA Network Open.*

assessed by asking how the individual felt over the past 7 days. To assess impression of improvement, individuals were asked to describe the change (if any) in feelings about themselves since before starting this trial.

The Patient-Reported Outcomes Measurement Information System (PROMIS) includes validated assessment tools that measure health concepts and symptoms applicable to a range of health conditions. The PROMIS Sleep Disturbance (short form) questionnaire was used to assess sleep disturbance over the past seven days at each timepoint. A reduction in sleep disturbance score over time indicates less sleep disturbance or a perceived improvement in sleep quality.[7-9]

The sum T-score of the SCI Functional Index (SCI-FI) was calculated from the physical function item banks for basic mobility, self-care, fine motor, ambulation, wheelchair mobility, and assistive technology.[10-15]

The sum T-score of the SCI-QOL Emotional Health domain was calculated for anxiety, depression, positive affect and well-being, grief-loss, self-esteem, trauma, resilience, and stigma (short form).[16-21] Scoring for the SCI-QOL Emotional Health domain was split between negative constraints: anxiety, depression, grief-loss, trauma, and stigma and positive aspects: positive affect and well-being, self-esteem, and resilience. Improvements in emotional health were reflected by higher positive aspect and lower negative constraint scores over time.

The sum T-score of the SCI-QOL Social Participation domain was calculated for the ability to participate in social roles and activities, satisfaction with social roles and activities, and independence (short form).[22] Higher scores reflected better functioning and more independence.

Self-reported methods and measures of bowel function for the frequency of bowel evacuation episodes, time per episode, number of self-reported "natural" bowel movements, amount of bowel evacuation medications used (e.g., laxatives, suppositories, and/or stool softeners), frequency of enemas used, frequency of digital stimulation needed per week, stool consistency (by the Bristol Stool Scale[23]), and frequency of bowel incontinence episodes. Participants self-report methods and measures of bowel function within the last 7 days for: a. Frequency of bowel evacuation episodes; b. Time spent per bowel evacuation in minutes/day; c. Number of self-reported bowel accidents in the past month; d. Number of self-reported "natural" bowel movements in the past week, and e. The use of bowel evacuation medications (e.g., laxatives and/or stool softeners), frequency of enemas, and frequency of digital stimulation needed per week. Stool consistency was reported using the Bristol Stool Scale.[23] Participants reported satisfaction with their bowel management program and bowel control within the last month. All questions are provided with fixed-format answers adapted from Krogh, et al.[24-27]

Preliminary data in people with SCI who used an exoskeleton 4-6 hours per week suggested effects similar to those observed with an exercise program (refs).[28-30] Therefore, additional objective secondary outcomes were selected to determine change in visceral adipose tissue (VAT, by DXA)[5]. A blood draw was performed for the serum lipids for HDL-c, low density lipoprotein cholesterol (LDL-c), triglycerides (TG), and total cholesterol (TC). Lipid blood specimens were shipped to the Chair's Office for batch analysis by the James J. Peters VA Medical Center General Chemistry Laboratory (results were blinded). A blood draw was performed to obtain serum fasting plasma glucose (FPG) and fasting plasma insulin (FPI) values for the calculation of Homeostasis Model Assessment for Insulin Resistance (HOMA-IR).[31] The FPG and FPI blood samples were shipped to the Chair's Office for batch analysis in the Core Research Laboratory of the VA Rehabilitation Research and Development National Center for the Medical

Consequences of Spinal Cord Injury. The core laboratory technician was blinded to the participants, group assignment and time point of analyses.

All outcome measurements were assessed at baseline, after orientation/training (phase 1), and post randomization (phase 2) at two-months and four-months (primary outcome time point).

Data was collected on the number of steps taken in the device by week (recorded from the step log in the exoskeletal device). In a weekly log sheet, the EAW group reported the amount of time the device was used, the locations of use, and any reasons why the device was not used. Usual activities were recorded on a weekly basis for both groups using a fixed format weekly log form.[8,9]

Study Intervention Description

The study intervention consisted of standard of care (wheelchair use) for the SOC (control) group and SOC plus exoskeletal-assisted walking (EAW) of 20-30 in-hospital training sessions and four months of home/community use of the ReWalk™ exoskeletal device for the EAW (intervention) group. The EAW group were required to 1pass the EAW Advanced Skills Test[2] with their companion(s) prior to taking the ReWalk home for four months. During the four months intervention of home/community use, as per the specific recommendation by the study team during the home set-up, the EAW group was instructed to continue to participate in wheelchair or other non-exoskeletal, non-wheelchair usual activities and to also use the ReWalk in their home/community environment "at will". The SOC (control) group participated in four months of usual wheelchair use (or other non-exoskeletal, nonwheelchair activities). Both groups were required to complete a weekly Usual Activity log. Study team members recorded the number of steps taken from the step counter that is built-in to the ReWalk device throughout the four months of intervention. The location and time of the EAW activities were recorded by the participant in an EAW weekly log. Site team members contacted the participants in both groups on a weekly basis, either over the phone or through secure messaging using My HealtheVet to review the Usual and EAW Activity logs and to identify problems or issues that may have presented. Participants in both groups were encouraged to contact site team members with any questions pertaining to the study at any time. Participants in both groups were assessed on all outcomes at baseline, after training/orientation and returned to the site for outcome tests at month two and at the end of month four 4 (primary outcome assessment time point). Each of the 15 sites was expected to randomize between 4 and 24 participants. Adverse events were recorded in both groups.

eResults. Supplemental eResults

Global Impression of Change Scale (participant- and companion-rated):

The companion's impression of the participant's global change was used as a proxy validation of the participant's self-assessment. The companion's impression of the participant's Global Change in Severity of their SCI and impression of Improvement was consistent with the participant's rating (Table 3, main article).

Adverse and Serious Adverse Events:

Potential exoskeletal device-related safety concerns included falls while using the exoskeleton resulting in a fracture or other injury, fragility fractures during weightbearing while standing and stepping in persons with extremely low BMD, and skin abrasions from points of contact in the device. The risk of long bone fracture was addressed with strict hip and knee BMD exclusion criteria, however, no such criteria existed for BMD of the foot. Bi-lateral foot x-

rays were added to the screening process in the second year of the recruitment to rule out pre-existing, undiagnosed foot fractures.

Note, due to local hospital policy, some sites interpreted any hospitalization as an SAE, regardless of reason. As such, 3 of 34 screening SAEs and 11 of 46 post-randomization SAEs were because the participant lived a distance from the VA site and was admitted to the VA Medical Center study site in order to participate in the screening or post-randomization assessments over multiple days.

Screening: During Screening, one exoskeletal-possibly-related fracture and one non-related fracture from a motor vehicle accident occurred; both were labeled as SAEs due to hospitalizations. There were three additional non-exoskeletal-related fractures that occurred from a shower transfer and two wheelchair falls that were reported as AEs (no hospitalization). During Screening, a total of four fractures occurred during other activities not related to exoskeletal device use and one fracture occurred with use of the exoskeletal device. The possibly exoskeletal-related fracture occurred without trauma during one of the first sessions and the fracture was subsequently labelled by the radiologist as being “highly susceptible for non-disclosed left superior calcaneus fracture of indeterminate age”, suggesting a possible pre-existing, but undiagnosed fracture that reoccurred from weightbearing while in the exoskeletal device. Eleven skin abrasion AEs occurred during Screening that were exoskeletal-related (in 10 participants) and 16 were non-exoskeletal related (in 13 participants).

Screening Protocol Amendment: In the first year of enrollment, there were two calcaneus fractures (in two participants) that occurred. Both were diagnosed by a VA radiologist at each site as likely to have been pre-existing. In both incidences, the participant, clinical staff, and medical record were absent of knowledge of these fractures. The executive planning committee met and discussed these two unanticipated foot fractures. It was decided to create an amendment to include bi-lateral foot x-rays as part of the screening process to rule out potential pre-existing undiagnosed foot fractures.

Post-randomization: During training/orientation and the four-month home/community exoskeletal device use there were six fractures across both groups. One fracture (calcaneus) occurred during use of the exoskeletal device and five fractures occurred during daily activities (not related to the exoskeletal device). The exoskeletal-related fracture also occurred without trauma and was diagnosed as a “stress fracture of the left heel”.

The EAW group experienced 15 falls while in the exoskeleton (with no injury or mild injury) and 8 falls while not in the device. The SOC group had 17 falls (all occurring while not while in the exoskeleton). For both groups, the falls that occurred not in the exoskeletal device were mostly during transfers to or from their wheelchairs.

There were 15 superficial skin breakdown events (in 11 participants) attributed to the exoskeletal device (14 in EAW group and 1 in SOC during post-study exoskeletal training) and 41 skin issues (15 in the EAW group and 26 in the SOC group) that were not related to the exoskeletal device. No SAE skin abrasions occurred during the study.

<u>Fracture summary:</u> In addition to the two exoskeletal-related fractures (one in screening, one in post-randomization), there were nine non-exoskeletal related fractures (four in screening, five in post-randomization) that were related to wheelchair falls, transfers, or other non-exoskeletal device activities.

<u>Falls summary:</u> Overall, there were 17 falls while using the exoskeleton (two with mild injury and 14 with no injuries reported) and 30 falls that were not related to the exoskeleton, but from wheelchair transfers and other non-exoskeleton related events (14 had mild injuries, two resulted in fractures, and 13 had no injuries reported). Of the 45 falls, two were classified as SAEs; one while in the exoskeleton (no injury) and one during a bed-to-wheelchair transfer that resulted in femur fracture. Note, the local hospital policy for some sites was that any fall to the ground regardless of injury or not required categorizing as an SAE.

© 2024 Spungen AM et al. *JAMA Network Open.*

Supplemental eReferences

1. Sippel JL, Daly JE, Poggensee L, et al. Modernization of a Large Spinal Cord Injuries and Disorders Registry: The Veterans Administration Experience. *Arch Rehabil Res Clin Transl*. Dec 2022;4(4):100237. doi:10.1016/j.arrct.2022.100237
2. Spungen AM, Bauman WA, Biswas K, et al. The design of a randomized control trial of exoskeletal-assisted walking in the home and community on quality of life in persons with chronic spinal cord injury. *Contemp Clin Trials*. Sep 2020;96:106102. doi:10.1016/j.cct.2020.106102
3. Cirnigliaro CM, Myslinski MJ, La Fountaine MF, Kirshblum SC, Forrest GF, Bauman WA. Bone loss at the distal femur and proximal tibia in persons with spinal cord injury: imaging approaches, risk of fracture, and potential treatment options. *Osteoporos Int*. Mar 2017;28(3):747-765. doi:10.1007/s00198-016-3798-x
4. Morse LR, Biering-Soerensen F, Carbone LD, et al. Bone Mineral Density Testing in Spinal Cord Injury: 2019 ISCD Official Position. *J Clin Densitom*. Oct-Dec 2019;22(4):554-566. doi:10.1016/j.jocd.2019.07.012
5. Cirnigliaro CM, LaFountaine MF, Dengel DR, et al. Visceral adiposity in persons with chronic spinal cord injury determined by dual energy X-Ray absorptiometry. *Obesity*. 2015;23(9):1811-1817.
6. Horton JN. Nominal group technique. A method of decision-making by committee. *Anaesthesia*. Aug 1980;35(8):811-4. doi:10.1111/j.1365-2044.1980.tb03924.x
7. Patient Reported Outcomes Measurement Information System (PROMIS). Dynamic Tools to Measure Health Outcomes from the Patient Perspective http://www.nihpromis.org
8. Buysse DJ, Yu L, Moul DE, et al. Development and validation of patient-reported outcome measures for sleep disturbance and sleep-related impairments. *Sleep*. 2010;33(6):781.
9. Yu L, Buysse DJ, Germain A, et al. Development of short forms from the PROMIS™ sleep disturbance and sleep-related impairment item banks. *Behavioral sleep medicine*. 2012;10(1):6-24.
10. Heinemann AW, Dijkers MP, Ni P, Tulsky DS, Jette A. Measurement Properties of the Spinal Cord Injury-Functional Index (SCI-FI) Short Forms. *Archives of physical medicine and rehabilitation*. 2014;
11. Jette AM, Slavin MD, Ni P, et al. Development and initial evaluation of the SCI-FI/AT. *The journal of spinal cord medicine*. 2015;38(3):409-418.
12. Jette AM, Tulsky DS, Ni P, et al. Development and initial evaluation of the spinal cord injury-functional index. *Archives of physical medicine and rehabilitation*. 2012;93(10):1733-1750.
13. Keeney T, Slavin M, Kisala P, et al. Sensitivity of the SCI-FI/AT in Individuals With Traumatic Spinal Cord Injury. *Archives of physical medicine and rehabilitation*. 2018;99(9):1783-1788.
14. Slavin MD, Ni P, Tulsky DS, et al. Spinal Cord Injury–Functional Index/Assistive Technology Short Forms. *Archives of physical medicine and rehabilitation*. 2016;97(10):1745-1752. e7.
15. Tulsky DS, Jette AM, Kisala PA, et al. Spinal cord injury-functional index: item banks to measure physical functioning in individuals with spinal cord injury. *Archives of physical medicine and rehabilitation*. 2012;93(10):1722-1732.
16. Kisala PA, Tulsky DS, Kalpakjian CZ, et al. Measuring anxiety after spinal cord injury: Development and psychometric characteristics of the SCI-QOL Anxiety item bank and linkage with GAD-7. *The journal of spinal cord medicine*. 2015;38(3):315-325.
17. Kisala PA, Tulsky DS, Pace N, Victorson D, Choi SW, Heinemann AW. Measuring stigma after spinal cord injury: Development and psychometric characteristics of the SCI-QOL Stigma item bank and short form. *The journal of spinal cord medicine*. 2015;38(3):386-396.
18. Kisala PA, Victorson D, Pace N, Heinemann AW, Choi SW, Tulsky DS. Measuring psychological trauma after spinal cord injury: Development and psychometric characteristics of the SCI-QOL Psychological Trauma item bank and short form. *The journal of spinal cord medicine*. 2015;38(3):326-334.
19. Tulsky DS, Kisala PA, Kalpakjian CZ, et al. Measuring depression after spinal cord injury: Development and psychometric characteristics of the SCI-QOL Depression item bank and linkage with PHQ-9. *The journal of spinal cord medicine*. 2015;38(3):335-346.

© 2024 Spungen AM et al. *JAMA Network Open.*

20. Tulsky DS, Kisala PA, Victorson D, et al. Developing a contemporary patient-reported outcomes measure for spinal cord injury. *Archives of physical medicine and rehabilitation*. 2011;92(10):S44-S51.

21. Victorson D, Tulsky DS, Kisala PA, Kalpakjian CZ, Weiland B, Choi SW. Measuring resilience after spinal cord injury: Development, validation and psychometric characteristics of the SCI-QOL Resilience item bank and short form. *The journal of spinal cord medicine*. 2015;38(3):366-376.

22. Heinemann AW, Kisala PA, Hahn EA, Tulsky DS. Development and psychometric characteristics of the SCI-QOL Ability to Participate and Satisfaction with Social Roles and Activities item banks and short forms. *The journal of spinal cord medicine*. 2015;38(3):397-408.

23. Riegler G, Esposito I. Bristol scale stool form. A still valid help in medical practice and clinical research. *Techniques in coloproctology*. 2001;5(3):163-164.

24. Krogh K, Christensen P, Sabroe S, Laurberg S. Neurogenic bowel dysfunction score. *Spinal cord*. 2006;44(10):625-631.

25. Krogh K, Emmanuel A, Perrouin-Verbe B, Korsten M, Mulcahey M, Biering-Sørensen F. International spinal cord injury bowel function basic data set (Version 2.0). *Spinal Cord*. 2017;

26. Krogh K, Mosdal C, Laurberg S. Gastrointestinal and segmental colonic transit times in patients with acute and chronic spinal cord lesions. *Spinal Cord*. Oct 2000;38(10):615-21.

27. Krogh K, Perkash I, Stiens S, Biering-Sørensen F. International bowel function extended spinal cord injury data set. *Spinal Cord*. 2009;47(3):235-241.

28. Asselin PK KS, Kornfeld S, Cirnigliaro C, Agranova-Breyter I, Bauman WA, Spungen AM. Heart Rate and Oxygen Demand of Powered Exoskeleton-Assisted Walking in Persons with Paraplegia. *Under Review*.

29. Evans N, Hartigan C, Kandilakis C, Pharo E, Clesson I. Acute Cardiorespiratory and Metabolic Responses During Exoskeleton-Assisted Walking Overground Among Persons with Chronic Spinal Cord Injury. *Top Spinal Cord Inj Rehabil*. Spring 2015;21(2):122-32. doi:10.1310/sci2102-122

30. Kandilakis C, Sasso-Lance E. Exoskeletons for Personal Use After Spinal Cord Injury. *Arch Phys Med Rehabil*. Feb 2021;102(2):331-337. doi:10.1016/j.apmr.2019.05.028

31. Gayoso-Diz P, Otero-Gonzalez A, Rodriguez-Alvarez MX, et al. Insulin resistance index (HOMA-IR) levels in a general adult population: curves percentile by gender and age. The EPIRCE study. *Diabetes Res Clin Pract*. Oct 2011;94(1):146-55. doi:10.1016/j.diabres.2011.07.015

© 2024 Spungen AM et al. *JAMA Network Open.*

Supplement eAbbreviations

Abbreviation	
AEs	Adverse events
BMD	Bone mineral density
CSP	Cooperative Studies Program
COVID-19	Corona virus disease of 2019
DXA	Dual energy x-ray absorptiometry
EAW	Exoskeletal-assisted walking
FPG	Fasting plasma glucose
FPI	Fasting plasma insulin
FDA	Food and Drug Administration
GCP	Good Clinical Practice
HDL-c	High density lipoprotein cholesterol
HOMA-IR	Homeostasis Model Assessment for Insulin Resistance
ITT	Intent-to-treat
kg	Kilogram
LDL-c	Lipoprotein cholesterol
MCS/VR-36	Mental Component Summary of the Veterans Rand-36
PROMIS	Patient Reported Outcomes Measurement Information System
PMH	Physical-Medical-Health
QOL	Quality of life
RCT	Randomized control trial
SCI-FI	SCI Functional Index
SAEs	Serious adverse events
SCI/D	Spinal cord injuries and disorders
SCIDR	Spinal cord injuries and disorders registry
SCI	Spinal cord injury
SOC	Standard of care
TC	Total cholesterol
TG	Triglycerides
VA	Veterans Affairs
VHA	Veterans Health Administration
VSSC	Veterans Health Administration Service Support Center
VAT	Visceral adipose tissue

© 2024 Spungen AM et al. *JAMA Network Open.*

eFigure 1. Geographic locations of the Veterans Health Administration Spinal Cord Injury/Disorders (SCI/D) System of Care and the Participating Sites.

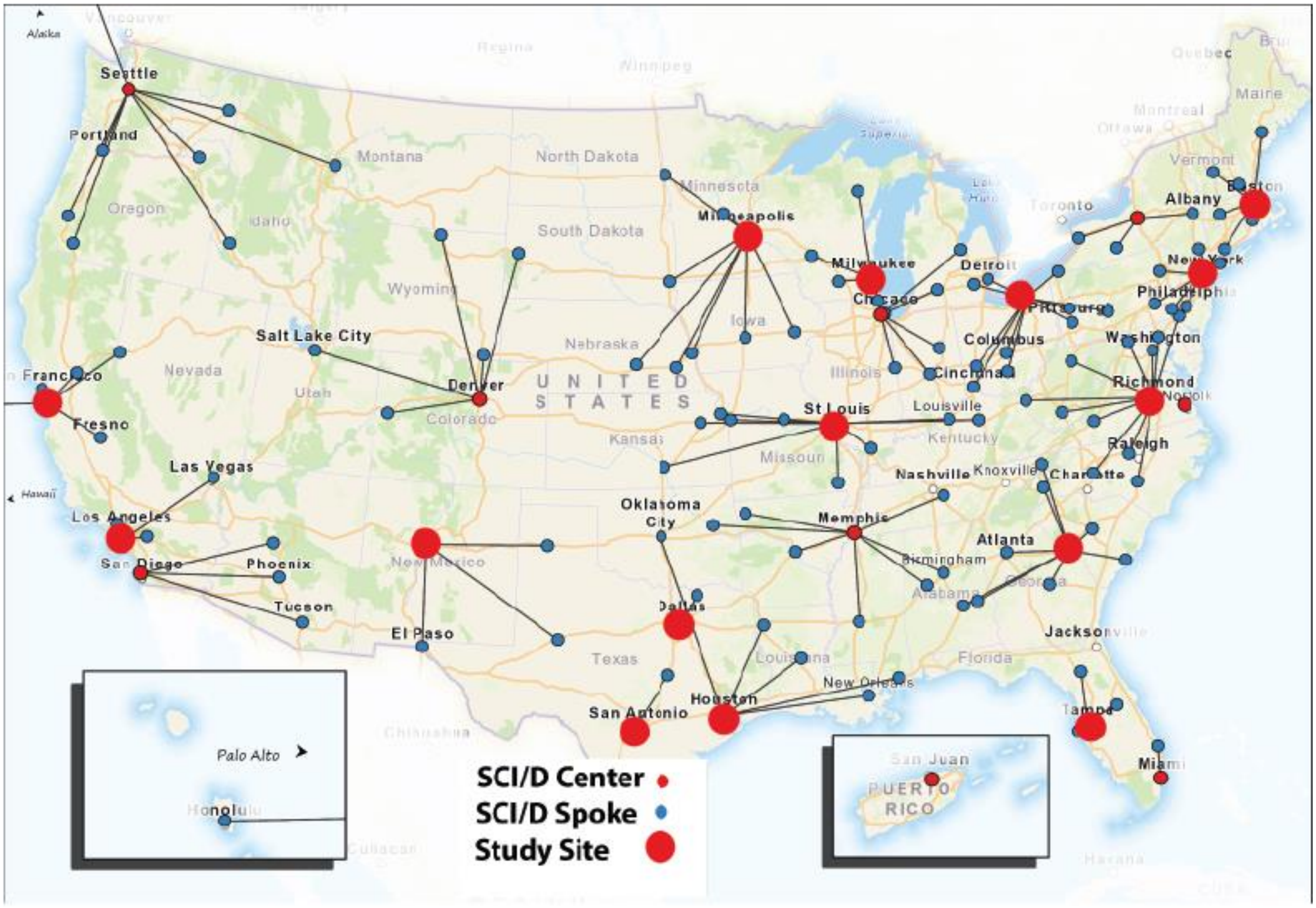

eFigure 1. Geographic locations of the Veterans Health Administration Spinal Cord Injury/Disorders (SCI/D) System of Care and the Participating Sites. The Veterans Health Administration, SCI/D System of care is comprised of 25 Centers (small and large red circles) and their spokes (small blue circles). Of the 25 SCI/D Centers, 15 were eligible and participated (large red circles) criteria previously described,[2] and 10 medical centers did not participate (small red circles) in this study. Participating SCI/D Centers are listed in alphabetical order: 1) Albuquerque, NM: Raymond G Murphy VA Medical Center, 2) Augusta, GA: Charlie Norwood VA Medical Center, 3) Boston, MA: VA Boston Healthcare System, 4) Bronx, NY: James J. Peters VA Medical Center, 5) Cleveland, OH: Louis Stokes Cleveland VA Medical Center, 6) Dallas, TX: VA North Texas Health Care System, 7) Houston, TX: Michael E. DeBakey VA Medical Center, 8) Long Beach, CA: VA Long Beach Healthcare System, 9) Milwaukee, WI: Clement J. Zablocki VA Medical Center, 10) Minneapolis, MN: Minneapolis VA Healthcare System, 11) Palo Alto, CA: VA Palo Alto Health Care System, 12) Richmond, VA: McGuire Veterans Medical Center, 13) San Antonio, TX: South Texas Veterans Health Care System-Audie Murphy Division, 14) St. Louis, MO: VA St. Louis Health Care System-Jefferson Barracks, and 15) Tampa, FL: James A. Haley VA Hospital.

© 2024 Spungen AM et al. *JAMA Network Open.*

eTables

eTable 1. Participant and Companion Eligibility Criteria	
**Inclusion Criteria**	
**Veteran/Active Duty Participants:**	
1.	Veterans or active duty military personnel who are at least 18 years of age;
2.	Traumatic or non-traumatic SCI ≥6 months duration of SCI;
3.	Wheelchair-user for indoor and outdoor mobility;
4.	Anthropometric compatibility with the device:
	a. Weight <220 lb. (100 kg),
	b. Thigh length between 14 and 19 in (36 and 48 cm),
	c. Shank length between 17 and 22 in (43 and 55 cm);
5.	Able to hold the crutches in hands without modifications;
6.	Able to have a companion who can attend approximately one-third of the training sessions who will learn how to assist them at home and in the community; and
7.	Able to provide informed consent.
**Companion Participants:**	
1.	Male/female greater than or equal to 18 years of age;
2.	Demonstrates understanding of the time commitment to be a companion;
3.	The companion and user are willing to receive training on how to assist the user with learning the device;
4.	Agrees to ensure that the exoskeleton is used with the crutches at all times;
5.	Site Investigator must deem the companion physically able to assist the participant with tasks outlined in the skills inventory (i.e. the companion is able-bodied, is able to bend, stoop, squat, kneel, etc.).
**Exclusion Criteria**	
**Veteran/Active Duty Participants:**	
1.	Diagnosis of neurological injury other than SCI;
2.	Progressive condition that would be expected to result in changing neurological status;
3.	Severe concurrent medical disease, illness or condition judged to be contraindicated by the site physician;
4.	Unhealed or unstable traumatic or high impact lower extremity fracture of any duration that is in the clinical judgement of the study physician to be exclusionary for standing and walking[1];
5.	Knee (proximal tibia and/or distal femur) BMD <0.60 gm/cm^2;
6.	Total hip BMD T-scores < -3.5;
7.	Fragility, minimal trauma or low impact fracture of the lower extremity since SCI[1];
8.	Untreatable severe spasticity judged to be contraindicated by the site physician;
9.	Flexion contracture >15º at the hip and/or >10º at the knee;
10.	Limitations in ankle range of motion that cannot be adapted with an orthotic device (plantar flexion >0º);
11.	Untreated/uncontrolled hypertension (SBP>140 mmHg; DBP >90 mmHg);
12.	Unresolved orthostatic hypotension (SBP <90 mmHg; DBP <60 mmHg) or as judged to be contraindicated by the site physician;
13.	Current pressure ulcer of the arms, trunk, pelvic area, or lower extremities;
14.	Psychopathology documentation in the medical record that may conflict with study objectives; and/or
15.	Pregnancy or women who plan to become pregnant during the study period.

eTable 1. Abbreviations: lb=pounds; kg=kilograms; in=inches; cm=centimeters; SCI=spinal cord injury; gm=grams; BMD=bone mineral density; m/s=meters per second; SBP=systolic blood pressure; and DBP=diastolic blood pressure.
[1]See eTable 2 for definitions of traumatic and fragility fractures.

© 2024 Spungen AM et al. *JAMA Network Open.*

eTable 2. Fracture Definition for Eligibility Criteria	
**Traumatic or high impact fracture**: Fracture from a forceful event, such as seen in any, or all of the following, but not limited to these circumstances:	
√	Fracture from a motor vehicle accident;
√	Fracture from a fall from a height greater than adult height standing (i.e. down steps or stairs); and/or
√	Fracture from a heavy object falling on any lower extremity body part.
**Fragility, minimal trauma, or low impact fracture** in the nonSCI population is defined by the National Osteoporosis Foundation: "Any fall from a standing height or less, that results in a fracture." Normal bones should be able to sustain a fall from this height, without a fracture, unless there is some underlying cause to suspect a bone disorder, such as osteoporosis or osteopenia that weakens bone structure.<br>In SCI, a **fragility fracture** may include any, or all, and are not limited to the following conditions and/or circumstances:	
√	Fracture that occurred without the person having knowledge of the occurrence or cause;
√	Fracture that resulted from a fall from a wheelchair, bed, toilet, etc.;
√	Fracture that occurred while performing stretching;
√	Fracture that resulted from, or during, a transfer;
√	Fracture from bumping or banging the lower extremity;
√	Fracture from dropping the foot to the ground or wheelchair footplate;
√	Fracture from a light object falling on any lower extremity body part; and/or
√	Fracture from carrying something or someone in their lap.

© 2024 Spungen AM et al. *JAMA Network Open.*

eTable 3. Reasons for Screen Failures and Study Withdrawals by Group

**A. Exclusion Reasons for Screening Failure**[1]		
Declined to participate, No. (% Consented, % Screened)	53 (13%), (17%)	
Significant BMD loss	64 (15%), (30%)	
Contracture / ROM / spasticity	39 (9%), (19%)	
Fracture history	21 (5%), (10%)	
Anthropometric / weight	27 (6%), (13%)	
Level of SCI / neurological status	21 (5%), (10%)	
Medical complication(s)	31 (7%), (15%)	
No companion / home not suitable	15 (4%), (7%)	
Other / physician discretion	9 (2%), (4%)	
Failed EAW basic skills test	32 (8%), (15%)	
**B. Reasons for Study Withdrawal by Group After Randomization**	**EAW** (N=78)	**SOC** (N=83)
Participant voluntarily withdrew, No. (% of randomized)	8 (10%)	7 (8%)
Lost to follow-up (unknown location)	1 (1%)	1 (1%)
Failed EAW advanced skills test	1 (1%)	n/a
Non-compliance	3 (4%)	1 (1%)
Other	3 (4%)	2 (2%)
Adverse event	0 (0%)	0 (0%)
Serious adverse event	4 (5%)	0 (0%)
Administrative withdrawal due to COVID-19 pandemic	13 (17%)	15 (18%)
Total	33 (42%)	26 (31%)

eTable 3. Abbreviations: BMD=Bone mineral density, SCI=Spinal cord injury, ROM=Range of motion, A five-session exoskeletal-assisted walking (EAW) basic skills test was a part of the screening procedures for both study groups prior to randomization, EAW=Exoskeletal-assisted walking group, and SOC=Standard of care group.
[1]Note, some participants are counted twice, but under different exclusions.

© 2024 Spungen AM et al. *JAMA Network Open.*

**eTable 4. Results of Self-reported Bowel Function for each Time Point Assessment and Group**				
	**EAW**	**SOC**	*P* value [1]	*P* value [2]
**Bowel control within the last 7 days**				
Baseline, No.	78	81	0.25	0.07
No leakage or accidents, No. (%)	70 (89.7)	64 (79.0)		
Leakage or an accident 1-2 times	7 (9.0)	14 (17.3)		
Leakage or an accident 3-4 times	1 (1.3)	2 (2.5)		
Leakage or an accident 5-6 times	0 (0.0)	1 (1.2)		
Leakage or an accident 7 or more times	0 (0.0)	0 (0.0)		
Training/Orientation	54	66	0.60	0.19
No leakage or accidents, No. (%)	46 (85.2)	50 (75.8)		
Leakage or an accident 1-2 times	7 (13.0)	12 (18.2)		
Leakage or an accident 3-4 times	1(1.9)	3 (4.5)		
Leakage or an accident 5-6 times	0 (0.0)	1 (1.5)		
Leakage or an accident 7 or more times	0 (0.0)	0 (0.0)		
Two Month (Intervention)	48	58	0.56	0.48
No leakage or accidents, No. (%)	42 (87.5)	45 (77.6)		
Leakage or an accident 1-2 times	6 (12.5)	9 (15.5)		
Leakage or an accident 3-4 times	0 (0.0)	2 (3.4)		
Leakage or an accident 5-6 times	0 (0.0)	1 (1.7)		
Leakage or an accident 7 or more times	0 (0.0)	1 (1.7)		
Four Month (Intervention)	45	56	0.60	0.76
No leakage or accidents, No. (%)	31 (68.9)	43 (76.8)		
Leakage or an accident 1-2 times	12 (26.7)	11 (19.6)		
Leakage or an accident 3-4 times	2 (4.4)	1 (1.8)		
Leakage or an accident 5-6 times	0 (0.0)	0 (0.0)		
Leakage or an accident 7 or more times	0 (0.0)	1 (1.8)		
**Required enemas or irrigations**	**EAW**	**SOC**	*P* value [1]	*P* value [2]
Baseline, No.	78	81	0.67	0.54
None/never, No. (%)	49 (62.8)	46 (56.8)		
Only once	5 (6.40	4 (4.9)		
A few times	2 (2.6)	6 (7.4)		
Most times (but not all)	5 (6.4)	7 (8.6)		
Every time	17 (21.8)	18 (22.2)		
Training/Orientation	53	66	0.45	0.35
None/never, No. (%)	37 (69.8)	40 (60.6)		
Only once	1 (1.9)	1 (1.5)		
A few times	2 (2.38)	6 (9.1)		
Most times (but not all)	0 (0.0)	3(4.5)		
Every time	13 (24.5)	16 (24.2)		
Two Month (Intervention)	48	59	0.87	0.91
None/never, No. (%)	29 (60.4)	33 (55.9)		
Only once	1 (2.1)	4 (6.8)		
A few times	3 (6.3)	3 (5.1)		
Most times (but not all)	2 (4.2)	3 (5.1)		
Every time	13 (27.1)	16 (27.1)		
Four Month (Intervention)	45	56	0.80	0.59

© 2024 Spungen AM et al. *JAMA Network Open.*

None/never, No. (%)	30 (66.7)	33 (58.9)		
Only once	1 (2.2)	4 (7.1)		
A few times	4 (8.9)	4 (7.1)		
Most times (but not all)	1 (2.2)	2 (3.6)		
Every time	9 (20.0)	13 (23.2)		
**Required oral medications to help**	**EAW**	**SOC**	*P* value [1]	*P* value [2]
Baseline, No.	78	81	0.30	0.33
None/never, No. (%)	40 (51.3)	46 (56.8)		
Only once	3 (3.8)	1 (1.2)		
A few times	4 (5.1)	9 (11.1)		
Most times (but not all)	4 (5.1)	6 (7.4)		
Every time	27 (34.6)	19 (23.5)		
Training/Orientation	54	66	0.55	0.33
None/never, No. (%)	26 (48.1)	38 (57.6)		
Only once	2 (3.7)	2 (3.0)		
A few times	5 (9.3)	7 (10.6)		
Most times (but not all)	4 (7.4)	1(1.5)		
Every time	17 (31.5)	18 (27.3)		
Two Month (Intervention)	48	59	0.94	0.52
None/never, No. (%)	26 (54.2)	35 (59.3)		
Only once	1 (2.1)	1 (1.7)		
A few times	3 (6.3)	5 (8.5)		
Most times (but not all)	3 (6.3)	3 (5.1)		
Every time	15 (31.3)	15 (25.4)		
Four Month (Intervention)	45	56	0.36	0.47
None/never, No. (%)	22 (48.9)	33 (58.9)		
Only once	1 (2.2)	3 (5.4)		
A few times	6 (13.3)	2 (3.6)		
Most times (but not all)	3 (6.7)	2 (3.6)		
Every time	13 (28.9)	16 (28.6)		
**Needed manual/digit stimulation to move the bowels**	**EAW**	**SOC**	*P* value [1]	*P* value [2]
Baseline, No.	78	81	0.63	0.63
None/never, No. (%)	20 (25.6)	16 (19.8)		
Only once	3 (3.8)	7 (8.6)		
A few times	6 (7.7)	4 (4.9)		
Most times (but not all)	9 (11.5)	11 (13.6)		
Every time	40 (51.3)	43 (53.1)		
Training/Orientation	54	66	0.41	0.31
None/never, No. (%)	18 (33.3)	14 (21.2)		
Only once	0 (0.0)	3 (4.5)		
A few times	5 (9.3)	6 (9.1)		
Most times (but not all)	6 (11.1)	9 (13.60		
Every time	25 (46.3)	34 (51.5)		
Two Month (Intervention)	48	59	0.41	0.33
None/never, No. (%)	15 (31.3)	11 (18.6)		
Only once	2 (4.2)	2 (3.4)		
A few times	2 (4.2)	7 (11.9)		
Most times (but not all)	4 (8.3)	7 (11.9)		
Every time	25 (52.1)	32 (54.2)		

© 2024 Spungen AM et al. *JAMA Network Open.*

Four Month (Intervention)	45	56	0.92	0.72
None/never, No. (%)	11 (24.4)	11 (19.6)		
Only once	1 (2.2)	3 (5.4)		
A few times	5 (11.1)	6 (10.7)		
Most times (but not all)	4 (8.9)	4 (7.1)		
Every time	24 (53.3)	32 (57.1)		
**Frequency of bowel movements per week**	**EAW**	**SOC**	*P* value [1]	*P* value [2]
Baseline, No.	77	81	0.51	0.08
7 times or more, No., (%)	29 (37.7)	37 (45.7)		
5-6 times	14 (18.2)	16 (19.8)		
3-4 times	25 (32.5)	18 (22.2)		
1-2 times	8 (10.4)	10 (12.3)		
None	1 (1.3)	0 (0.0)		
Training/Orientation	53	66	0.49	0.13
7 times or more, No., (%)	18 (34.0)	24 (36.4)		
5-6 times	12 (22.6)	14 (21.2)		
3-4 times	15 (28.3)	24 (36.4)		
1-2 times	7 (13.2)	3 (4.5)		
None	1 (1.9)	1 (1.5)		
Two Month (Intervention)	47	59	0.42	0.09
7 times or more, No., (%)	15 (31.9)	27 (45.8)		
5-6 times	13 (27.7)	10 (16.9)		
3-4 times	13 (27.7)	16 (27.1)		
1-2 times	6 (12.8)	6 (10.2)		
None	0 (0.0)	0 (0.0)		
Four Month (Intervention)	45	55	0.96	0.73
7 times or more, No., (%)	18 (40.0)	23 (41.8)		
5-6 times	7 (15.6)	9 (16.4)		
3-4 times	16 (35.6)	16 (29.1)		
1-2 times	4 (8.9)	6 (10.9)		
None	0 (0.0)	1 (1.8)		
**Time spent to have a bowel movement per day**	**EAW**	**SOC**	*P* value [1]	*P* value [2]
Baseline, No.	78	81	0.07	0.61
5 to 15 minutes, No., (%)	29 (37.2)	19 (23.5)		
15 to 30 minutes	12 (15.4)	19 (23.5)		
30 to 60 minutes	22 (28.2)	34 (42.0)		
1 to 3 hours	14 (17.9)	9 (11.1)		
More than 3 hours	1 (1.3)	0 (0.0)		
Training/Orientation	54	64	0.15	0.64
5 to 15 minutes, No., (%)	17 (31.5)	12 (18.8)		
15 to 30 minutes	11 (20.4)	18 (28.1)		
30 to 60 minutes	14 (25.9)	25 (39.1)		
1 to 3 hours	12 (22.2)	9 (14.1)		
More than 3 hours	0 (0.0)	0 (0.0)		
Two Month (Intervention)	48	59	0.09	0.58
5 to 15 minutes, No., (%)	15 (31.3)	8 (14.3)		
15 to 30 minutes	10 (20.8)	17 (28.8)		
30 to 60 minutes	12 (25.0)	24 (40.7)		
1 to 3 hours	11 (22.9)	7 (11.9)		

© 2024 Spungen AM et al. *JAMA Network Open.*

More than 3 hours	0 (0.0)	1 (1.7)		
Four Month (Intervention)	45	56	0.18	0.24
5 to 15 minutes, No., (%)	14 (31.1)	8 (14.3)		
15 to 30 minutes	10 (22.2)	14 (25.0)		
30 to 60 minutes	10 (22.2)	22 (39.3)		
1 to 3 hours	10 (22.2)	11 (19.6)		
More than 3 hours	1 (2.2)	1 (1.8)		
**Total time per week spent to move bowels**	**EAW**	**SOC**	*P* value [1]	*P* value [2]
Baseline, No.	78	80	0.35	0.85
1 to 2 total hours, No., (%)	32 (41.0)			
2 to 4 total hours	18 (23.1)			
4 to 6 total hours	8 (10.3)			
6 to 8 total hours	15 (19.2)			
More than 8 total hours	5 (6.4)			
Training/Orientation	54	65	0.52	0.71
1 to 2 total hours, No., (%)	20 (37.0)			
2 to 4 total hours	13 (24.1)			
4 to 6 total hours	9 (16.7)			
6 to 8 total hours	8 (14.8)			
More than 8 total hours	4 (7.4)			
Two Month (Intervention)	48	59	0.21	0.42
1 to 2 total hours, No., (%)	18 (37.5)	11 (18.6)		
2 to 4 total hours	10 (20.8)	20 (33.9)		
4 to 6 total hours	8 (16.7)	14 (23.7)		
6 to 8 total hours	6 (12.5)	8 (13.6)		
More than 8 total hours	6 (12.5)	6 (10.2)		
Four Month (Intervention)	45	55	0.63	0.56
1 to 2 total hours, No., (%)	17 (37.8)	18 (32.7)		
2 to 4 total hours	14 (31.1)	14 (25.5)		
4 to 6 total hours	6 (13.3)	8 (14.5)		
6 to 8 total hours	4 (8.9)	11 (20.0)		
More than 8 total hours	4 (8.9)	4 (7.3)		
**Felt bloated, distended, other bowel-related discomfort**	**EAW**	**SOC**	*P* value [1]	*P* value [2]
Baseline	77	79	0.24	0.72
Not at all, No., (%)	40 (51.9)	36 (45.6)		
Once (1 day)	11 (14.3)	16 (20.3)		
A few times (2 or more days)	18 (23.4)	23 (29.1)		
Most of the time (but not every day)	4 (5.2)	4 (5.1)		
Every day; felt discomfort all the time	4 (5.2)	0 (0.0)		
Training/Orientation	54	64	0.98	0.68
Not at all, No., (%)	26 (48.1)	29 (45.3)		
Once (1 day)	10 (18.5)	13 (20.3)		
A few times (2 or more days)	12 (22.2)	13 (20.3)		
Most of the time (but not every day)	4 (7.4)	7 (10.9)		
Every day; felt discomfort all the time	2 (3.7)	2 (3.1)		
Two Month (Intervention)	46	59	0.49	0.09
Not at all, No., (%)	24 (52.2)	27 (25.8)		
Once (1 day)	5 (10.9)	14 (23.7)		
A few times (2 or more days)	12 (26.1)	14 (23.7)		

© 2024 Spungen AM et al. *JAMA Network Open.*

Most of the time (but not every day)	2 (4.3)	1 (1.7)		
Every day; felt discomfort all the time	3 (6.5)	3 (5.1)		
Four Month (Intervention)	44	55	0.92	0.71
Not at all, No., (%)	18 (40.9)	25 (45.5)		
Once (1 day)	9 (20.5)	12 (21.8)		
A few times (2 or more days)	14 (31.8)	13 (23.6)		
Most of the time (but not every day)	1 (2.3)	2 (3.6)		
Every day; felt discomfort all the time	2 (4.5)	3 (5.5)		
**Stool consistency from Bristol Stool Scale**	**EAW**	**SOC**	*P* value [1]	*P* value [2]
Baseline, No.	76	79	0.93	0.76
Separate hard lumps, No., (%)	5 (6.6)	5 (6.3)		
Sausage-shaped, but lumpy	15 (19.7)	13 (16.5)		
Like sausage, cracks on surface	16 (21.1)	21 (26.6)		
Like a snake, smooth and soft	27 (35.5)	26 (32.9)		
Soft blobs (passed easily)	8 (10.5)	6 (7.6)		
Fluffy pieces, a mush stool	5 (6.6)	7 (8.9)		
Watery, entirely liquid	0 (0.0)	1 (1.3)		
Training/Orientation	52	65	0.10	0.56
Separate hard lumps, No., (%)	3 (5.8)	2 (3.1)		
Sausage-shaped, but lumpy	7 (13.5)	12 (18.5)		
Like sausage, cracks on surface	12 (23.1)	16 (24.6)		
Like a snake, smooth and soft	16 (30.8)	27 (41.5)		
Soft blobs (passed easily)	10 (19.2)	2 (3.1)		
Fluffy pieces, a mush stool	4 (7.7)	5 (7.7)		
Watery, entirely liquid	0 (0.0)	1 (1.5)		
Two Month (Intervention)	47	58	0.61	0.36
Separate hard lumps, No., (%)	2 (4.3)	4 (6.9)		
Sausage-shaped, but lumpy	7 (14.9)	12 (20.7)		
Like sausage, cracks on surface	12 (25.5)	13 (22.4)		
Like a snake, smooth and soft	16 (34.0)	22 (37.9)		
Soft blobs (passed easily)	4 (8.5)	2 (3.4)		
Fluffy pieces, a mush stool	5 (10.6)	2 (3.4)		
Watery, entirely liquid	1 (2.1)	3 (5.2)		
Four Month (Intervention)	42	55	0.09	0.11
Separate hard lumps, No., (%)	1 (2.4)	4 (7.3)		
Sausage-shaped, but lumpy	5 (11.9)	10 (18.2)		
Like sausage, cracks on surface	13 (31.0)	14 (25.5)		
Like a snake, smooth and soft	11 (26.2)	21 (38.2)		
Soft blobs (passed easily)	5 (11.9)	2 (3.6)		
Fluffy pieces, a mush stool	7 (16.7)	2 (3.6)		
Watery, entirely liquid	0 (0.0)	2 (3.6)		

eTable 4. [1]*P* value from Fisher's Exact test. [2]P value from Cochran-Armitage Trend Test. Note, the overall P value from generalized linear mixed model including treatment, week, treatment by week interaction, and baseline was P = 0.490.

eTable 5. Results for Visceral Adipose Tissue Mass, Lipid Profile, and HOMA-IR for each Time Point Assessment and Group

Visceral Adipose Tissue Mass (g) [1]		EAW Group		SOC Group	*P* value
Baseline, No., median (IQR)	78	1601 (689 to 2394)	83	1496 (673 to 2227)	0.60
Training/Orientation	54	1685 (773 to 2476)	64	1598 (725 to 2322)	0.52
Two Month (Intervention)	47	1767 (699 to 2619)	57	1468 (765 to 2280)	0.30
Four Month (Intervention)	45	1631 (677 to 2590)	55	1329 (728 to 2219)	0.28
Change Training/Orientation	54	-11.5 (-173 to 99)	64	-7.5 (-151 to 107)	0.65
Change Two Month (Intervention)	47	-67 (-260 to 175)	57	10 (-138 to 128)	0.24
Change Four Month (Intervention)	45	-63 (-266 to 113)	55	-41 (-165 to 119)	0.62
**High Density Lipoprotein Cholesterol (mg/dL) [2]**		**EAW Group**		**SOC Group**	***P* value**
Baseline, No., median (IQR)	66	43.0 (38.0 to 51.0)	73	47.0 (37.0 to 52.0)	0.62
Training/Orientation	48	42.5 (39.5 to 55.0)	59	44.0 (38.0 to 52.0)	0.80
Two Month (Intervention)	44	43.5 (39.0 to 50.5)	51	47.0 (39.0 to 54.0)	0.41
Four Month (Intervention)	38	44.5 (40.0 to 52.0)	48	46.5 (41.5 to 53.0)	0.36
Change Training/Orientation	47	1.0 (-2.0 to 4.0)	59	1.0 (-4.0 to 4.0)	0.83
Change Two Month (Intervention)	42	1.0 (-2.0 to 5.0)	51	1.0 (-3.0 to 6.0)	0.63
Change Four Month (Intervention)	37	2.0 (-3.0 to 5.0)	48	-1.0 (-6.5 to 5.0)	0.46
**Low Density Lipoprotein cholesterol (mg/dL) [2]**		**EAW Group**		**SOC Group**	***P* value**
Baseline, No., median (IQR)	65	103.2 (86.8 to 120.6)	71	94.4 (70.8 to 123.0)	0.21
Training/Orientation	48	105.7 (86.8 to 130.7)	59	96.6 (77.8 to 115.8)	0.12
Two Month (Intervention)	43	105.8 (87.4 to 127.8)	51	94.0 (68.2 to 122.6)	0.03
Four Month (Intervention)	37	105.8 (77.8 to 127.2)	47	94.6 (68.4 to 116.6)	0.22
Change Training/Orientation	46	-5.9 (-17.6 to 10.0)	59	-0.2 (-11.2 to 17.2)	0.20
Change Two Month (Intervention)	40	-0.9 (-15.2 to 9.3)	51	-2.2 (-9.6 to 14.6)	0.92
Change Four Month (Intervention)	35	-3.8 (-14.4 to 14.8)	47	3.2 (-12.2 to 17.4)	0.38
**Triglycerides (mg/dL) [3]**		**EAW Group**		**SOC Group**	***P* value**
Baseline, No, median (IQR)	66	104.0 (72.0 to 153.0)	73	110.0 (82.0 to 157.0)	0.48
Training/Orientation	48	109.5 (79.0 to 147.0)	59	109.0 (80.0 to 173.0)	0.94
Two Month (Intervention)	44	104.5 (82.5 to 150.5)	51	118.0 (74.0 to 184.0)	0.95
Four Month (Intervention)	38	132.0 (85.0 to 168.0)	48	111.5 (80.5 to 153.5)	0.34
Change Training/Orientation	47	2.0 (-30.0 to 32.0)	59	3.0 (-34.0 to 38.0)	0.87
Change Two Month (Intervention)	42	2.5 (-23.0 to 24.0)	51	1.0 (-28.0 to 40.0)	0.83
Change Four Month (Intervention)	37	15.0 (-18.0 to 39.0)	48	-5.5 (-26.0 to 22.0)	0.17
**Total cholesterol (mg/dL) [2]**		**EAW Group**		**SOC Group**	***P* value**
Baseline, No., median (IQR)	66	178.5 (150.0 to 197.0)	73	167.0 (145.0 to 190.0)	0.51
Training/Orientation	48	176.5 (153.5 to 205.0)	59	168.0 (150.0 to 185.0)	0.10
Two Month (Intervention)	44	176.5 (152.0 to 205.5)	51	165.0 (141.0 to 190.0)	0.14
Four Month (Intervention)	38	177.0 (152.0 to 202.0)	48	170.5 (138.5 to 200.0)	0.29
Change Training/Orientation	47	1.0 (-19.0 to 13.0)	59	1.0 (-14.0 to 19.0)	0.84
Change Two Month (Intervention)	42	2.0 (-14.0 to 13.0)	51	0.0 (-8.0 to 16.0)	0.86
Change Four Month (Intervention)	37	-3.0 (-15.0 to 20.0)	48	-0.5 (-13.0 to 15.5)	0.90
**Fasting plasma glucose (mg/dL) [4]**		**EAW Group**		**SOC Group**	***P* value**
Baseline, No., median (IQR)	68	92.7 (87.7 to 98.6)	73	92.0 (83.7 to 102.0)	0.70
Training/Orientation	50	93.9 (88.1 to 104.0)	60	94.3 (88.3to 103.0)	0.88
Two Month (Intervention)	44	93.4 (86.4 to 99.8)	48	92.9 (86.6 to 104.0)	0.91
Four Month (Intervention)	40	94.3 (86.6 to 105.0)	49	95.0 (84.5 to 104.0)	0.88
Change Training/Orientation	50	1.8 (-3.0 to 7.3)	60	1.0 (-5.9 to 9.5)	0.98
Change Two Month (Intervention)	43	-1.1 (-7.1 to 5.0)	48	1.0 (-5.4 to 10.5)	0.55
Change Four Month (Intervention)	39	-0.4 (-5.0 to 7.2)	48	2.0 (-5.1 to 9.5)	0.77

© 2024 Spungen AM et al. *JAMA Network Open.*

Fasting plasma insulin (mlU/L) [5]	EAW Group		SOC Group		*P* value
Baseline, No., median (IQR)	67	9.1 (7.1 to 17.4)	72	8.4 (5.6 to 16.9)	0.47
Training/Orientation	50	10.0 (7.2 to 18.1)	59	9.4 (6.6 to 18.8)	0.99
Two Month (Intervention)	44	9.4 (7.2 to 18.8)	48	8.3 (6.9 to 17.1)	0.32
Four Month (Intervention)	40	11.2 (8.3 to 18.3)	49	10.2 (7.2 to 17.5)	0.48
Change Training/Orientation	50	0.5 (-0.9 to 2.5)	59	-0.1 (-2.0 to 2.6)	0.24
Change Two Month (Intervention)	43	0.2 (-0.9 to 1.7)	48	-0.3 (-1.4 to 1.8)	0.32
Change Four Month (Intervention)	39	0.7 (-0.4 to 2.4)	48	0.3 (-1.7 to 2.2)	0.28
**HOMA-IR**	**EAW Group**		**SOC Group**		***P* value**
Baseline, No., median (IQR)	67	2.2 (1.6 to 3.9)	72	1.9 (1.1 to 3.7)	0.50
Training/Orientation	50	2.4 (1.7 to 4.6)	59	2.9 (1.5 to 4.6)	0.91
Two Month (Intervention)	44	2.3 (1.5 to 4.4)	47	2.1 (1.6 to 4.0)	0.50
Four Month (Intervention)	40	2.6 (1.9 to 4.1)	48	2.5 (1.6 to 4.5)	0.81
Change Training/Orientation	50	0.2 (-0.4 to 0.7)	59	0.1 (-0.6 to 0.7)	0.52
Change Two Month (Intervention)	43	0.0 (-0.5 to 0.5)	47	0.0 (-0.4 to 0.5)	0.94
Change Four Month (Intervention)	39	0.3 (-0.1 to 0.6)	47	0.1 (-0.5 to 0.7)	0.51

eTable 5. Abbreviations: g-grams, IQR, interquartile range Q1-Q3; mg/dL=milligrams per deciliter, mlU/L=milli units per liter, and HOMA-IR=Homeostasis Model Assessment for Insulin Resistance. Continuous non-normally distributed data are presented as median, IQR using Wilcoxon tests.

[1] Visceral fat mass was determined by dual energy x-ray absorptiometry (DXA) scanning.

[2] HDL-C, LDL-C, and total cholesterol SI conversion factor 0.0259

[3] Triglyceride SI conversion factor 0.0113

[4] Glucose SI conversion factor 0.0555.

[5] Insulin SI conversion factor 6.945.

© 2024 Spungen AM et al. *JAMA Network Open.*

**eTable 6. Location, Surface, and Step Count for Exoskeletal Device Usage and Reasons for not using the Device**						
**Exoskeletal device use,** No., mean (SD), median, minimum and maximum range						
Number of weeks (of 12 weeks)		53	7.7 (5.3)	8	0	16
**Location of exoskeleton use,** No., minutes/week, mean (SD), median, minimum and maximum range						
In Home		46	27.3 (36.3)	12	0	128
Someone Else's Home		46	0.6 (1.8)	0	0	8
Shopping Mall/Restaurant/Place of Worship		46	6.2 (12.9)	0	0	60
Park		46	4.0 (15.5)	0	0	98
Outside on Sidewalk/Street		46	25.6 (33.4)	10	0	109
Hospital/Clinic/Rehab		46	9.9 (15.7)	4	0	75
Other Locations		46	12.5 (27.4)	0	0	125
Total (minutes)		46	86.0 (45.8)	81	0	248
**Exoskeletal use by surface type,** No., minutes/week, mean (SD), median, minimum and maximum range						
Carpet		46	6.8 (18.3)	0	0	79
Tile/Wood/Smooth Surface		46	35.2 (33.2)	29	0	139
Dirt/Gravel/Grass/Cobblestone		46	4.8 (13.0)	0	0	70
Concrete/Asphalt/Cement		46	37.9 (31.8)	32	0	120
**Steps and miles walked in the exoskeleton,** No., steps/month, miles/month[1], mean (SD), median, minimum and maximum range						
Month 1	(steps)	32	5926 (6120)	3,651	493	30,378
	(miles)		1.70 (1.7)	1.04	0.14	8.60
Month 2	(steps)	42	4321 (4654)	2,438	426	18,802
	(miles)		1.23 (1.32)	0.69	0.12	5.30
Month 3	(steps)	28	6192 (10757)	3,954	616	57,766
	(miles)		1.76 (3.06)	1.12	0.18	16.4
Month 4	(steps)	37	5080 (7533)	2,492	250	41,011
	(miles)		1.44 (2.14)	0.71	0.07	11.70
**Reasons for not using the exoskeleton,** No. (%)						
Companion unavailable			177 (43.9)			
Illness-medical condition			70 (17.4)			
Busy-no time			58 (14.4)			
Travel			37 (9.2)			
Inclement weather			24 (6.0)			
Not motivated			13 (3.2)			
No reason provided			9 (2.2)			
Device malfunction			9 (2.2)			
Device not charged/unavailable			4 (1.0)			
No car available			2 (0.5)			
Totals			403 (100.0)			

eTable 6. Of the 78 participants that were randomized to the EAW group, 23 were early terminators during phase 1 (orientation/training), 55 made it to the start of phase 2 (intervention), of which only 45 completed phase 2, and 46 used the device for at least 1 or more weeks in phase 2 (intervention). Steps were recorded from the exoskeleton step counter on a weekly basis.
[1] Estimated miles walked in the exoskeleton were calculated based on an average step length of 18 inches using the equation [(number of steps x 18")/12" / 5,280' = miles].

© 2024 Spungen AM et al. *JAMA Network Open.*

eTable 7. Self-Reported Record of Usual Weekly Activities During the Intervention Phase by Group											
**Activity,** No., minutes/week, mean (SD), median, minimum and maximum range											
	**Phase**	**EAW Group [1]**					**SOC Group**				
Stretching	1	76	106 (113)	68	0	588	82	134 (136)	88	0	585
	2	52	123 (140)	80	0	617	74	133 (128)	101	0	443
Weightlifting	1	76	45 (85)	10	0	494	82	57 (73)	32	0	288
	2	52	40 (89)	4	0	526	74	51 (82)	10	0	404
Push-ups, pull-ups, or dips	1	76	29 (76)	2	0	505	82	31 (52)	0	0	210
	2	52	30 (81)	1	0	528	74	25 (50)	0	0	222
Pushing Wheelchair for Exercise	1	76	119 (295)	28	0	2169	82	118 (233)	40	0	1680
	2	52	94 (195)	19	0	930	74	103 (217)	25	0	1620
Stationary Arm Cycle/Ergometer	1	76	8 (27)	0	0	194	82	19 (41)	0	0	224
	2	52	7 (26)	0	0	147	74	12 (30)	0	0	150
Wheelchair Dancing	1	76	1 (4)	0	0	28	82	21 (167)	0	0	1508
	2	52	1 (3)	0	0	14	74	16 (101)	0	0	855
Wheelchair Sports	1	76	27 (60)	0	0	293	82	23 (56)	0	0	338
	2	52	17 (42)	0	0	226	74	23 (64)	0	0	410
Non-wheelchair-based Activities	1	76	43 (130)	0	0	799	82	35 (95)	0	0	617
	2	52	54 (201)	0	0	1371	74	26 (69)	0	0	445
Rehabilitation Activities	1	76	37 (58)	4	0	296	82	49 (107)	3	0	840
	2	52	37 (70)	4	0	394	74	36 (59)	0	0	240
Household Chores	1	76	247 (325)	134	0	1805	82	279 (332)	154	0	2069
	2	52	296 (495)	96	0	2940	74	229 (309)	147	0	1756
Other1	1	76	105 (179)	33	0	1070	82	142 (205)	54	0	860
	2	52	155 (300)	54	0	1778	74	165 (294)	38	0	1867
Other2	1	76	65 (291)	0	0	2400	82	39 (89)	0	0	480
	2	52	64 (194)	0	0	964	74	43 (104)	0	0	508
Overall time by Phase in usual activities	1	76	830 (677)	594	25	3164	82	945 (728)	774	95	3653
	2	52	914 (790)	626	76	3138	74	865 (717)	611	68	3347
**Total**		**76**	**830 (666)**	**623**	**28**	**3098**	**82**	**937 (754)**	**755**	**91**	**3509**

eTable 7. Abbreviations: Phase 1 - Orientation/training phase (approximately 1 month), Phase 2 - Post randomization/intervention (4 months), EAW=exoskeletal-assisted walking group, SOC=Standard of care group, No.=number of participants by Phase and group reporting results.

[1] Note, EAW activities were in excess of using the exoskeleton.

© 2024 Spungen AM et al. *JAMA Network Open.*

eTable 8. Serious Adverse and Adverse Events During Screening and Post Randomization

**A. Screening** (No.=424), study relatedness	Not	Possibly	Definitely	Total				
**Serious Adverse Events (SAE)**								
Any [1], No. events, (No. participants)	29 (23)	2 (2)	3 (2) [1]	34 (27)				
Exoskeletal device bone fracture, No.	0	1	0	1				
Non-Exoskeletal device bone fracture	1	0	0	1				
Exoskeletal device fall	0	0	0	0				
Non-Exoskeletal device fall	0	0	0	0				
**Adverse Events (AE)**								
Any [1,2], No. events, (No. participants)	90 (62)	25 (22)	30 (22) [1]	145 (87)				
Exoskeletal device bone fracture	0	0	0	0				
Non-Exoskeletal device bone fracture	3 (3)	0	0	3 (3)				
Exoskeletal device skin issue	0	4 (4)	7 (6)	11 (10)				
Non-Exoskeletal device skin issue	14 (13)	0	2 (1) [3]	16 (13)				
Exoskeletal device fall	0	0	2 (2)	2 (2)				
Non-Exoskeletal device fall [4]	5 (5)	0	0	5 (5)				
**B. Post Randomization** (No.=161), study relatedness	**EAW Group** (N=78)				**SOC Group** (N=83)			
	Not	Possibly	Definitely	Total	Not	Possibly	Definitely	Total
**Serious Adverse Events (SAE)**								
Any [1], No. of events, (No. of participants)	11 (8)	2 (2)	5 (3) [1]	18 (12)	20 (13)	0	8 (1) [1]	28 (14)
Exoskeletal device bone fracture	0	0	1	1	0	0	0	0
Non-Exoskeletal device bone fracture	1	0	0	1	1	0	0	1
Exoskeletal device fall	0	0	1 [5]	1	0	0	0	0
Non-Exoskeletal device fall [4]	0	0	0	0	1 [4]	0	0	1
**Adverse Events (AE)**								
Any [1,2], No. events, (No. participants)	92 (41)	27 (18)	38 (24) *	157 (55)	156 (42)	4 (4)	5 (2) [1]	165 (43)
Exoskeletal device bone fracture	0	0	0	0	0	0	0	0
Non-Exoskeletal device bone fracture	3 (3)	0	0	3 (3)	0	0	0	0
Exoskeletal device skin issue	0	5 (5)	9 (7)	14 (12)	0	1 [8]	0	1
Non-Exoskeletal device skin issue	13 (10)	1 (1)	1 (1)	15 (12)	26 (14)	0	0	26 (14)
Exoskeletal device fall	0	0	14 (13) [7]	14 (13)	0	0	0	0
Non-Exoskeletal device fall [4,6]	6 (5)	2 (2) [6]	0	8 (7)	16 (7)	0	0	16 (7)

eTable 8. Screening includes Eligibility screening, baseline testing, and five-session basic skills exoskeletal-assisted walking training; Post randomization includes Phase 1 (Orientation/Training) and Phase 2 (Four months Intervention and Post Study EAW exoskeletal-assisted walking.

[1] Four events during the Screening (3 SAEs, 1 AE) and 18 events during Post Randomization (11 SAEs, 7AEs) were inpatient admissions for scheduled study evaluations and were not due to illness or injury.

[2] Adverse events meeting the SAE criteria were included only in the SAE count.

[3] Both events were caused by Loftstrand crutches.

[4] Constitutes a fall from a wheelchair, during a transfer, or other situation not in the exoskeleton.

[5] Fall in exoskeleton outside on driveway; participant reported no injury. Some sites required "any fall" to be labeled as a SAE.

[6] Falls that were possibly study-related, but not from the exoskeletal device.

[7] Not categorized as home or hospital for some falls.

[8] Occurred during the post study training sessions for SOC group.

© 2024 Spungen AM et al. *JAMA Network Open.*
