# Supplementary material for: Exoskeletal-Assisted Walking in Veterans With Paralysis: A Randomized Clinical Trial
Source: JAMA Netw Open. 2024 Sep 4;7(9):e2431501. doi: 10.1001/jamanetworkopen.2024.31501 (PMC11375472; doi:10.1001/jamanetworkopen.2024.31501)
Supplement: Supplement 2. — Trial Protocol and Statistical Analysis Plan [file jamanetwopen-e2431501-s002.pdf]

VA Cooperative Studies Program Protocol #2003

Exoskeletal-Assisted Walking in Persons with SCI: Impact on Quality of Life

July 2021

Version 7.2

Study Chairperson: Ann M. Spungen, Ed.D, JJPVAMC Bronx, NY
Study Co-Chairperson: William A. Bauman, MD, JJPVAMC Bronx, NY

-PRIVILEGED AND CONFIDENTIAL-
Not to be Disseminated Beyond Official Use

TABLE OF CONTENTS

Glossary....................i
Executive Committee....................iii
Executive Summary....................vi
I. Introduction and Background....................1
A. Measurement of Quality of Life Outcomes in SCI....................5
B. Pilot Data....................9
C. Preliminary Data Employed for the Primary Outcome Variables....................11
D. Supporting Data for the Major Secondary Outcome Variable....................13
E. Secondary Outcomes....................14
II. Objectives....................17
A. Primary Objectives....................17
B. Major Secondary Objective....................17
C. Secondary Objectives....................17
D. Hypotheses....................18
III. Experimental Design....................19
A. Screening Phase....................19
B. Intervention Phase....................21
C. Post Study Participation for the Control Group....................22
D. Study Duration and Timeline....................22
IV. Participant Recruitment....................27
A. Recruitment and Screening....................27
B. Randomization System....................27
C. Population to be Studied....................28
V. Intervention and Methods of Treatment....................28
VI. Methods of Follow-up....................29
VII. Methods of Assuring Uniformity of Intervention....................30
VIII. Determining Eligibility....................30

A. Inclusion/Eligibility Criteria 30
B. Exclusion Criteria 31
C. Exoskeleton Component (“Training the Trainers”) 33
D. ReWalk Fitting 33
E. EAW Basic Skills Test 34
F. EAW Walking Skills Progression 35
G. EAW Companion Training 36
H. EAW Advanced Skills Test 38
I. Weekly EAW Activity Log 40
IX. Outcome Measurements 40
A. Primary Outcomes. 41
B. Major Secondary Outcome 41
C. Secondary Outcomes 41
D. Additional Assessments 42
E. Quality Control (non-outcome) Measurements 42
F. Methods for Obtaining Outcome Measures Data 43
G. Schedule of Observations and Laboratory Tests 44
X. Performance Measures and Study Management 44
A. CSPCC 45
B. Office of the Chair 45
XI. Safety Monitoring 46
A. Study Group 46
B. Executive Committee 47
C. Data Monitoring Committee 47
D. VA Central Institutional Review Board (CIRB) 47
E. Definitions of Adverse Event (AE), Serious Adverse Event (SAE), and Unanticipated Adverse Device Effect (UADE) 48
F. Serious Adverse Event (SAE) Reporting 49
G. Adverse Event (AE) Reporting 49
H. Participant Safety Stopping Rules. 50

I. Site Participation Stopping Rules 51
J. Efficacy or Futility Criteria for Study Termination 51
XII. Quality Assurance Procedures 52
A. Summary of Monitoring and Auditing Plans 52
XIII. Statistical Analysis Plan (SAP) 53
A. Introduction 53
B. Sample Size 53
C. Feasibility Phase 55
D. Baseline Comparability 56
E. General Outcome Analysis Guidelines 56
F. Analysis of Primary and Major Secondary Outcome Measures 56
G. Secondary Analyses 57
H. Monitoring of Study by Study Group and Executive Committee 58
I. Study Monitoring by the Data Monitoring Committee 62
XIV. Qualifications 66
A. Site Qualifications 66
B. Site Participation 68
C. Office of the Chair Qualifications 70
XV. Plans to Assure Security and Confidentiality of Study Data 70
XVI. Data Management and Data Security Plans 71
XVII. Plans for Dissemination of Study Results 72
A. Publication of Research Results 72
B. Planned Publications 73
XVIII. Human Subjects Protection Considerations 74
A. Recruitment of Participants and Special Considerations 74
B. Inclusion of Women and Minorities in the Study 74
C. Description of the Consenting Process. 74
D. Participant Compensation 75
E. Safety Considerations 76

F. Foreseeable Risks 76
G. Risk Management and Emergency Response 77
H. Emergency Care Plan 79
I. Potential Benefits 79
XIX. Transition of Participants 80
XX. Impact of the SARS-CoV-2 (COVID-19) pandemic on screened participants 81
A. Background 81
B. Rationale. 82
C. Methods 86
D. Relevance 88
E. Limitations 90
XXI. References 90

Glossary	
**Acronym**	**Meaning**
10mWT	10 Meter Walk Test
6minWT	6 Minute Walk Test
AD	Autonomic Dysreflexia
AE	Adverse Event
AIS	American Spinal Injury International Standards Scale
ASIA	American Spinal Injury Association
BE	Bowel Evacuation
BMD	Bone Mineral Density
BP	Blood Pressure
CBC	Complete Blood Count
CBOCs	Community Based Outpatient Centers
CIRB	Central Institutional Review Board
CRF	Case Report Form
CRP	C-Reactive Protein
CSP	Cooperative Studies Program
CSPCC	Cooperative Studies Program Coordinating Center
CSPCRPCC	Cooperative Studies Program Clinical Research Pharmacy Coordinating Center
CSSEC	Cooperative Studies Scientific Evaluation Committee
DMC	Data Monitoring Committee
DVA	Department of Veterans Affairs
DXA	Dual energy X-Absorptiometry
EAW	Exoskeletal-Assisted Walking
FDA	Food and Drug Administration
FES	Functional Electrical Stimulation
FIM	Functional Independence Measurement
FPG	Fasting Plasma Glucose
FTE	Full Time Equivalent
FWA	Federal Wide Assurance
FY	Fiscal Year
GCP	Good Clinical Practice
H&PE	Health and Physical Exam
HDL-c	High Density Lipoprotein cholesterol
HIPAA	Health Insurance Portability and Accountability Act
HOMA-IR	Homeostasis Model of Assessment-Insulin Resistance
HR	Heart Rate
HRC	Human Rights Committee
HRQOL	Health-Related Quality of Life
ICH	International Conference on Harmonization
ISNCSCI	International Standards for Neurological Classification of SCI

ITTRS	Interactive Touch Tone Randomization System
JJPVAMC	James J. Peters VA Medical Center
LDL-c	Low Density Lipoprotein cholesterol
LOA	Level of Assistance
MCS	Mental Component Summary
MID	Minimally Important Difference
mL	Milliliters
NCMRR	National Center for Medical Rehabilitation Research
NIH	National Institute of Health
NINDS	National Institute of Neurological Disorders and Stroke
NODES	Network of Dedicated Enrollment Sites
ORO	Office of Research Oversight
pg	Picograms
PHI	Protected Health Information
PI	Primary Investigator
PMH	Physical-Medical Health
PPE	Personal Protective Equipment
PROMIS	Patient Reported Outcomes Measurement Information System
QC	Quality Control
QOL	Quality of Life
RPE	Rating of Perceived Exertion
RR&D	Rehabilitation Research and Development
SAE	Serious Adverse Event
SCI	Spinal Cord Injury
SCI/D	Spinal Cord Injury/Disease
SCI-FI	Spinal Cord Injury Functional Index
SD	Standard Derivative
SMART	Site Monitoring Auditing and Resource Team
SOC	Standard of Care
TC	Total Cholesterol
TG	Triglycerides
TNF	Tumor Necrosis Factor
TUG	Timed-Up-and Go
UADE	Unanticipated Adverse Device Effect
U/L	Units per liter
UTI	Urinary Tract Infection
US	United States
VHA	Veterans Health Administration
VINCI	VA Informatics and Computing Infrastructure
VR-36	Veterans Rand 36

CSP #2003

Exoskeletal-Assisted Walking in Persons with SCI: Impact on Quality of Life

Executive Committee Members

Ann M. Spungen, EdD, Chairperson — **James J Peters VA Medical Center (JJPVAMC), Bronx, NY**

Name	Ann M. Spungen, EdD
Title	Associate Director, National Center of Excellence for the Medical Consequences of SCI
Location	James J. Peters VA Medical Center<br>130 West Kingsbridge Road, 7A-13<br>Bronx, NY 10468
Phone	718.584.9000, x5814 (office)<br>914.954.3515 (cell)
VA Email	ann.spungen@va.gov
nonVA email	ann.spungen@gmail.com
Altern. email	kenlys.fajardo@va.gov (Dr. Spungen's Administrative Assistant)

William A. Bauman, MD, Co-Chairperson — **James J Peters VA Medical Center (JJPVAMC), Bronx, NY**

Name	William A. Bauman, MD
Title	Director, National Center of Excellence for the Medical Consequences of SCI
Location	James J. Peters VA Medical Center<br>130 West Kingsbridge Road, 7A-13<br>Bronx, NY 10468
Phone	718-584-9000, x5428 (office)<br>914-329-4772 (cell)
VA Email	william.bauman@va.gov
nonVA email	wabauman@earthlink.net
Altern. email	kenlys.fajardo@va.gov (Dr. Bauman's Administrative Assistant)

Karen M. Jones, MS, Study Biostatistician — **Cooperative Studies Program Coordinating Center (CSPCC), Perry Point VAMC Perry Point, MD**

Name	Karen Jones, MS
Title	Biostatistician, CSPCC
Location	Cooperative Studies Program Coordinating Center<br>Department of Veterans Affairs Medical Center<br>P.O. Box 1010

167	Perry Point, MD 21902

Phone 410-642-2411, x2/5297 (office)
VA Email Karen.jones2@va.gov
nonVA email none
Altern. email none

Sunil Sabharwal, MD, Site Investigator **VA Boston Healthcare System West Roxbury, MA**

Name Sunil Sabharwal, MD
Title Network SCI Director
Location VA Boston Healthcare System
1400 VFW Parkway, SCI-128
West Roxbury, MA 02132
Phone 857-203-6574 (office)
VA Email sunil.sabharwal2@va.gov
nonVA email none
Altern. email marilyn.kendall@va.gov (Dr. Sabharwal's Administrative Assistant)

Lance Goetz, MD, Site Investigator **Hunter Holmes McGuire VA Medical Center Richmond, VA**

Name Lance Goetz, MD
Title Staff Physician, Spinal Cord Injury and Disorders Service
Location Hunter Holmes McGuire VA Medical Center
1201 Broad Rock Blvd
Richmond, VA 23249
Phone 804-675-5455 (office)
VA Email lance.goetz@va.gov
nonVA email none
Altern. email none

Kevin T. White, MD, Site Investigator **James A. Haley Veterans Hospital Tampa, FL**

Name Kevin T. White, MD
Title Chief, Spinal Cord Injury Service
Location James A. Haley Veterans Hospital
13000 Bruce B. Downs Blvd.
Tampa, FL 33612
Phone 813-972-2000, x6400 (office)
813-695-1575 (cell)
VA Email Kevin.White2@va.gov
nonVA email none
Altern. email Gail.Hartmaier@va.gov (Dr. White's Administrative Assistant)

Peter Gorman, MD, Chief, PM&R — **Baltimore VA Medical Center, Baltimore, MD**

Name	Peter Gorman, MD MS
Title	Attending Physician, PM&R Service
Location	VA Maryland Healthcare System<br>10 North Greene Street<br>Baltimore, MD 21201
Phone	410-448-6261 (office)
VA Email	peter.gorman@va.gov
nonVA email	PGORMAN@umm.edu
Altern. email	LCasey@umm.edu (Dr. Gorman's Administrative Assistant)

Steven C. Kirshblum, MD, Medical Director — **Kessler Institute for Rehabilitation (KIR), West Orange, NJ**

Name	Steven C. Kirshblum, MD
Title	Medical Director and Director of SCI Services
Location	Kessler Institute for Rehabilitation<br>1199 Pleasant Valley Way<br>West Orange, NJ 07052
Phone	973-731-3900
VA Email	none
nonVA email	SKirshblum@KESSLER-REHAB.com
Altern. email	RBucci@KESSLER-REHAB.com (Dr. Kirshblum's Administrative Assistant)

Amanda Snodgrass, Clinical Research Pharmacist — **Cooperative Studies Program Clinical Research Pharmacy Coordinating Center (CSPCRPCC), Albuquerque, NM**

Name	Amanda Snodgrass, PharmD
Title	CSP, Adverse Event Specialist
Location	VA Cooperative Studies Program, CSPCRPCC<br>2401 Centre Ave<br>Albuquerque, NM 87106
Phone	505-248-3203 (office)<br>505-206-8449 (cell)
VA Email	Amanda.snodgrass@va.gov
nonVA email	none
Altern. email	none

EXECUTIVE SUMMARY/ABSTRACT

Background Veterans with spinal cord injury (SCI) have many adverse secondary medical and quality of life (QOL) changes as a result of immobilization. Veterans with SCI who have completed rehabilitation after injury and are unable to ambulate receive a wheelchair as standard of care (SOC) for mobility. Powered exoskeletons are a technology that has recently become available as an alternate form of mobility by providing an external framework for support and computer controlled motorized hip and knee joints to assist with overground ambulation.

Research Questions (Objectives) Will Veterans with chronic SCI of ≥six months duration, who are medically stable and who use a wheelchair as SOC plus an exoskeletal-assisted walking (EAW) device in their home and community environments have clinically meaningful net improvements in mental health, bladder, bowel, and pain patient-reported outcomes compared with those who use only the SOC? Additionally, will the use of an EAW device for four months in the homes and/or communities of the participants result in a reduction of total body fat mass?

Study Design A two-group (Intervention and Control), randomized, clinical trial will be performed with a one-year feasibility component. The Intervention group will receive SOC plus EAW. The Control group will receive SOC only. The study will require seven years in total to complete and includes fifteen VA SCI Services as study sites. A feasibility phase will be employed using a staggered start by initially starting six sites, four sites starting one year later, and 5 other sites starting two years later. These initial six sites will be used to assess the start-up activities [hiring, training, equipment procurement, Central Institutional Review Board (CIRB) paperwork, etc.], the rate of recruitment, and any other issues that may be of value for the successful completion of the study. Lessons learned will be implemented for the remaining sites.

Relevance to VA In pilot studies conducted at the James J. Peters VA Medical Center, Bronx, NY, improvements in mental-emotional health, physical health and body composition were demonstrated by providing the participants the ability to walk for 4 to 6 hours per week over the course of three to five months. As of July 2014, a Class 2 designation was established by the Food and Drug Administration (FDA) for "powered exoskeletons". To date, one device (ReWalk™) has received FDA Class 2 approval for institutional and home use and is currently available by prescription. The Department of Veterans Affairs (DVA) is the largest single provider to persons with SCI in the USA, caring for about 26,000 of the 42,000 estimated Veterans with SCI. The VA presently lacks the infrastructure to support Veterans with SCI to train to use this device in order to make this technology available for the home/community use. A controlled research study would be anticipated to be the optimal manner to demonstrate the efficacy, amount of use and safety of a powered exoskeleton in the home and community environments; findings would be immediately transferable to clinical care.

Number of Research Participants (Sample Size**)** One hundred-sixty participants (80/group) will be randomized. Each of the 15 study sites will be expected to pre-screen ≥100 potential participants, screen ≥60 participants, to reach the target of 160 randomized over 15 sites.

Participating Sites Fifteen SCI Services will be selected on the basis of potential recruitment numbers (N=13,606 total Veterans with SCI in the sites' catchment areas and N=7,022 followed annually at these sites) and geographic location, to permit an even distribution across the country. The fifteen sites include: Boston, Richmond, St. Louis, Tampa, Milwaukee, Minneapolis, Dallas, Houston, Palo Alto, Long Beach, Augusta, San Antonio, Bronx, Cleveland, and Albuquerque. Of these fifteen sites, five are VA Cooperative Studies Program (CSP) Network of Dedicated Enrollment Sites (NODES).

Duration of Participant Intake (Study Duration) The CS #2003 study duration is projected to be a total of seven years: The initial six sites will have a start-up year, followed by participant enrollment/data collection during years 1-4, and continued data collection/closeout during year 5, for a total of six years. The next four sites will begin the start-up year one year after the first six sites and follow the same enrollment, data collection and closeout schedule over the next five years. The next five sites will begin start-up one year after the four sites and follow the same enrollment, data collection and closeout schedule. All fifteen sites will be closed out after six years, and there is an additional year for the Coordinating Center and Chairperson's Office to complete data analysis and manuscript writing, thus the study total time is seven years. Participants in both groups will be asked to commit 33 weeks to this study. Participants in the Control arm will be offered an additional 8 weeks to receive EAW training in the medical centers, without outcome data being collected.

Treatment (follow-up) The intervention being tested is four months of home and/or community use of a powered exoskeleton.

Definition of Participant Samples (Study Population) One-hundred sixty male or female Veterans or military members with chronic SCI, ≥six months duration, ≥18 years of age, functional use of their hands, medically stable, and wheelchair users for indoor and outdoor mobility, will be eligible for screening. All potential participants will be Veterans or military members with SCI. Study participants will generally be outpatients with the exception of those inpatients who meet the eligibility criteria, and who are approved by the Site Investigator (e.g., some inpatients may have been admitted for a wheelchair fitting or another non-medical reason). Non-veterans with SCI will not be eligible.

Treatment Arms All participants will receive four months of treatment, randomized into two arms: SOC plus EAW or SOC only.

Endpoints Primary outcome one will be the Mental Component Summary of the Veterans Rand-36 (MCS/VR-36). Primary outcome two will be the sum T-score of the SCI-QOL bladder management difficulties, bowel management difficulties and pain interference item banks. The major secondary outcome will be total body fat mass. The two primary and the major secondary outcomes will be analyzed as the proportion of participants in each group who achieved a clinically meaningful change in score. The endpoint will be success or failure for these outcomes.

Protocol amendment due to COVID-19 pandemic: In Fiscal Year 2020 (FY20), Quarter 2, the United States (US) experienced a coronavirus pandemic. As a result, beginning in March of 2020, most of the country was placed on social distancing restrictions and/or quarantine. CSP #2003 was placed on a voluntary administrative hold on March 16, 2020. During this pandemic, the Chair's Office became aware of potential loss of services for our Veterans with SCI such as caregivers, medical appointments, wheelchair repair and others, and has proposed an amendment to CSP #2003. This amendment is to conduct an SCI-specific survey to determine the effect of the pandemic on Veterans with SCI who have been previously screened for participation in this study. A follow-up survey and blood testing for the virus antibodies will also be conducted at least 1 month after the initial survey. The administrative hold has been lifted for the COVID-19 survey and blood draw.

I. INTRODUCTION AND BACKGROUND

Veterans with spinal cord injury (SCI) have an overabundance of adverse secondary medical and quality of life (QOL) changes as a result of paralysis and immobilization. Veterans with SCI who have completed rehabilitation after injury and are unable to ambulate receive a wheelchair as standard of care (SOC) for mobility. Powered exoskeletons are a technology that has recently become available as an alternate form of mobility by providing an external framework for support and computer controlled motorized hip and knee joints to assist with overground ambulation. These exoskeletal devices have been used primarily in a hospital setting but have been approved for home/community use since 2014. Identifying efficacy and safety of a powered exoskeleton to be used in the home or community environment for Veterans with SCI is an important issue.

<u>Physical, medical, and health-related quality of life changes following SCI</u>*:* SCI results in paralysis and near permanent loss of voluntary function below the level of lesion. Those with more severe neurological lesions lose the ability to perform the weight bearing activities of standing and walking. Most become wheelchair users for indoor and outdoor mobility. The loss of the ability to walk is an obvious consequence of SCI. However, there are many less observable complications that result from loss of walking activity that serve to reduce quality of life (QOL), such as compromised bowel and bladder function, increased pain, extreme changes in body composition, and difficulty sleeping. On a daily basis, coping with loss of bowel [1-3] and bladder [4, 5] function, chronic pain [6], and difficulty sleeping [7] takes a tremendous, and often unrecognized, toll on emotional and physical well-being. Chronic constipation [8] and difficulty with evacuation are time consuming, taking hours per week, requiring prescription of multiple types of stool softeners and laxatives, and often the use of enemas and/or digital stimulation to perform a bowel evacuation, all of which are over-shadowed by the ever-present anxiety of a potential bowel accident [1, 9]. Standing alone has not been demonstrated to be effective in decreasing time to first stool for a bowel evacuation [10]. Constant sitting often leads to chronic back pain and, in those with some sensation below their lesion, leg pain is also prevalent [11]. Uncontrolled muscle spasms, which are similar to leg cramps in those who are not paralyzed, also cause discomfort and pain and may be severe at times [12]. Sleep is unremittingly interrupted due to spasms, pain and other conditions directly related to paralysis [7, 13, 14]. Immobilization from SCI has severe consequences on body composition [15-18]. Within the first two years after SCI, persons with motor-complete lesions may be expected to gain as much as 10 kg of total body fat mass and to lose a similar amount of lean tissue mass (Spungen, et.al., manuscript under review). With these

extreme changes in body composition and the forced reduction in levels of activity, there are associated carbohydrate and lipid metabolism abnormalities [19-21]. Impaired glucose tolerance or diabetes mellitus occurs in as many as 60% of the SCI population [21-24]. HDL-c levels below 35 mg/dL (an independent risk factor for heart disease) are reported in 24 to 40% of persons with SCI [25, 26].

Ambulation efforts for persons with SCI*:* Clinical therapeutic options available to assist persons with SCI to stand and walk have been, by and large, unsuccessful when extended to the home environment. For people with motor-complete SCI who have good upper body strength, or those with motor-incomplete SCI, such as those who have some voluntary movement in the legs or can even weight bear to some degree, various types of gait orthoses have had limited success in allowing the individual to stand and walk [27-29]. The high energy cost of using gait orthoses, though, limits use to those with sufficient upper body strength to ambulate for short bouts, and extended walking does not occur with the use of gait orthoses [27, 30]. Locomotor training, with either manual assistance by two therapists or robotic assistance with a Lokomat to move the legs and feet in a walking motion over a treadmill while the patient is suspended in an overhead harness to off-load body weight, has demonstrated positive benefits on cardiovascular function, self-esteem, and quality of life [31-35]. These body-weight supported treadmill training methods require significant staff commitment, equipment and space, and these approaches are not practical for home, work or community use; thus, these benefits are lost with the discontinuation of the gait training program. Ambulation has also been accomplished with functional electrical stimulation (FES), but these FES systems have the challenge of controlling the joint movements appropriate for gait and because FES is an anaerobic form of exercise, the users invariably experience rapid muscle fatigue [36]. To reduce the challenge of controlling joint movement, hybrid systems have combined FES and a passive orthotic [37, 38], and have demonstrated improvements in energy expended while walking, but the challenge of rapid muscle fatigue still persists. Several other types of hybrid systems, such as stored-energy hybrid systems which use FES to stimulate the muscle group for rotation of one joint and transfer energy to drive another joint, or controlled brake orthoses which use FES to power the movement but incorporate a computer controlled brake system to lock out the joint and control joint speeds during ambulation, have been or are being developed, but these approaches are still confined to the research setting [39-42]. As such, most of these devices for assisted FES walking have remained as concepts or prototypes and have not been commercialized, making viable options for walking outside the hospital or rehabilitation setting relatively limited.

Use of a wheelchair for mobility and daily activities remains the standard of care for Veterans who are unable to stand and walk as a result of paralysis due to SCI. When a person becomes injured, initial rehabilitation is prescribed for usually no longer than four to six months, depending on the severity of the SCI. Once discharged, there are very few rehabilitation programs or strategies for the home or as an outpatient that are being offered to Veterans with chronic SCI. As such, our Veterans with chronic SCI remain wheelchair-bound and are part of a demographic that is one of the most sedentary known to man, with resultant numerous secondary medical consequences of longstanding, severe immobilization.

Exoskeletal-assisted walking (EAW) in SCI*:* Since 2011, a relatively new technology, that permits assisted standing and walking overground, has been available for people with immobilization due to paralysis from SCI. This robot-like exoskeletal technology uses a computerized, powered exoskeleton support frame, attached to the pelvis and legs, that allows a person with paralysis to stand and walk [43, 44]. Early in 2011, Dr. Ann M. Spungen and her team at the VA Rehabilitation Research & Development (RR&D) National Center of Excellence for the Medical Consequences of Spinal Cord Injury, James J. Peters VA Medical Center (JJPVAMC), Bronx, NY, began conducting pilot studies in the safety and efficacy of exoskeletal-assisted walking in persons with SCI. They were the first Veterans Affairs (VA) investigators to acquire exoskeletal-assisted walking (EAW) technology to test the safety and efficacy and for use in persons with SCI [45-47]. This powered exoskeleton (ReWalk™) [48] allows a person with paraplegia to stand and walk overground for extended periods of time.

One distinct advantage of the powered exoskeleton over previous rehabilitation walking modalities is that it has the potential to be prescribed for use in the home and community. The exoskeletal technology has the potential to change the field of rehabilitation medicine by offering a modality that permits assisted walking not only in the rehabilitation setting, but also in the home and community environment. This opens the possibility for continuous use and an option for increasing upright physical activity throughout life. To date, several DVA SCI Services have tentatively embarked on using this technology in the clinical institutional setting.

The investigators at JJPVAMC have been studying the efficacy of using this device in persons with SCI on mobility and walking skills, quality of life, medical, and health-related outcomes. These investigators have demonstrated that people with SCI who are otherwise wheelchair-dependent are able to tolerate performing EAW for four to six hours per week without difficulty, and they report improvements in QOL outcomes after approximately two to four months of exoskeletal training. Specific benefits have been documented in body composition [49], energy expenditure [50], bowel function, sleep, and pain reduction (See Preliminary data). The reported benefits from increased activity through upright mobility are not unexpected. It has long been appreciated that increasing activity and exercise in a sedentary person has numerous health benefits. It is relevant to note that, to date, the exoskeletal-assisted walking participants with SCI at the JJPVAMC have unanimously reported that being able to walk again has markedly changed their perceptions of their lives for the better, and this beneficial appraisal of participants is even with the use of this device being highly restricted to use in the institutional setting. It remains incredibly disheartening for these participants to complete the research program and no longer have a viable option for performing overground walking, but this would be a viable option if prescribed for home use. The proposed VA Cooperative Study (CS #2003) would demonstrate the physical, mental, social, and emotional benefits of use of these robotic ambulatory devices in the home and community environments and, thus, have the potential to dramatically change the lives of our Veterans with SCI.

Of note, the ReWalk is not the only exoskeleton commercially available [51, 52], or with the potential to be commercially available [53], but this specific device is currently the only one with Food and Drug Administration (FDA) approval as a Class 2 device for rehabilitation and home use [54]. Findings from the proposed study would be readily transferable to any future powered exoskeleton devices that may become available from development from VA, academic, or the private sectors.

A. Measurement of Quality of Life Outcomes in SCI

<u>The Patient Reported Outcomes Measurement Information System (PROMIS) and the link to the SCI-QOL:</u> The National Institutes of Health (NIH) Roadmap for Medical Research in the 21st Century (now called the Common Fund) has supported major initiatives in biomedical research that no single institute could accomplish alone. One key Common Fund project was a multicenter cooperative group award to develop and validate a Patient-Reported Outcomes Measurement Information System (PROMIS U01 AR052177-02). PROMIS contributes to NIH re-engineering by building and validating common, accessible item banks to measure key symptoms and health concepts applicable to a range of chronic conditions. At the same time, the National Institute of Neurological Disorders and Stroke (NINDS) issued a request for proposals to construct a clinically relevant and useful health-related quality of life (HRQOL) measurement tool for major neurological diseases that affect the United States population. This measurement tool was developed to be consistent across the selected conditions to allow for cross-disease comparison, and yet flexible enough to capture condition-specific HRQOL issues. This contract was awarded to the same group that was developing PROMIS (Dr. David Cella and colleagues) and is called Neuro-QOL. The Neuro-QOL was a five year effort to construct a psychometrically-sound and clinically-relevant HRQOL measurement system for individuals with major neurological disorders [55]. Wherever possible, Neuro-QOL measures included relevant PROMIS items to allow for linking and cross-walking of scores between the two systems. This is valuable because a multiple sclerosis investigator who uses a PROMIS measure could also derive a Neuro-QOL score and compare results in his/her study to other multiple sclerosis studies that have used Neuro-QOL [56]. Early in the award period, a rigorous process was undertaken by investigators and outside experts to select 5 adult and 2 pediatric conditions (the budget would only allow this many) to represent the major issues and concerns faced by individuals with neurological disorders. After conducting interviews with 44 neurology professionals, an online survey with 89 members of the American Academy of Neurology and a day-long consensus meeting with an international panel of distinguished experts spanning the field of neurology, the following conditions were selected: stroke, multiple sclerosis, amyotrophic lateral sclerosis, Parkinson’s disease, epilepsy [adult and child], and muscular dystrophy. Even though there was considerable interest throughout this process to include SCI among the selected adult conditions, in the end, it was not included [57]. Nonetheless, it became clear that extending Neuro-QOL measurement development methodologies to SCI could significantly improve this field’s ability to assess important HRQOL outcomes and track clinical treatment changes. In 2008, working closely with members of the Neuro-QOL team, Dr. David Tulsky was awarded funding from two agencies of the NIH: NINDS

and the National Center for Medical Rehabilitation Research (NCMRR) to develop SCI-specific patient reported outcome measurement tools in physical-medical, emotional and social function (SCI-QOL) (NIH #5RO1HD054659). Additionally, the National Institute of Disability Rehabilitation Research (NIDRR) separately funded Drs. Tulsky and Alan Jette (from Boston University, Boston, MA) to develop a physical function index of HRQOL instruments for SCI (SCI-FI) (NIH #5RO1HD054659). The goal and a major strength of the SCI-QOL and SCI-FI projects were to embed Neuro-QOL items (which include verbatim PROMIS items) at the core of the new targeted SCI-specific item banks. The SCI-QOL utilized the PROMIS standards [58] for instrument development, incorporating both qualitative (i.e., focus groups, cognitive debriefing interviews) and quantitative (i.e., item response theory analyses and computerized adaptive testing) [59] methods to ensure that the final SCI-QOL was psychometrically sound while at the same time conceptually grounded to issues that are truly important to individuals with SCI [60, 61]. The final SCI-QOL includes 22 item response theory calibrated item banks/scales across the domains of Physical-Medical Health, Physical Functioning (SCI-FI), Social Participation, and Emotional Health. Notably, item banks that are based on PROMIS (e.g., Depression, Anxiety) or Neuro-QOL (e.g., Stigma, Ability to Participate in Social Roles and Activities) banks/items, the SCI-QOL scores have undergone a linear transformation back to the PROMIS or Neuro-QOL metric, respectively. In this way, SCI-QOL item banks are optimized for individuals with SCI (i.e., through selection and order of items administered) yet are directly comparable to individuals in the general population through this statistical transformation.

The NIH/NIDRR funded initiatives have supported the creation of innovative health-related QOL measurement approaches, specific for individuals with SCI. Two valid and useful SCI-specific,

Figure 3. SCI- QOL & SCI-FI Measurement Development

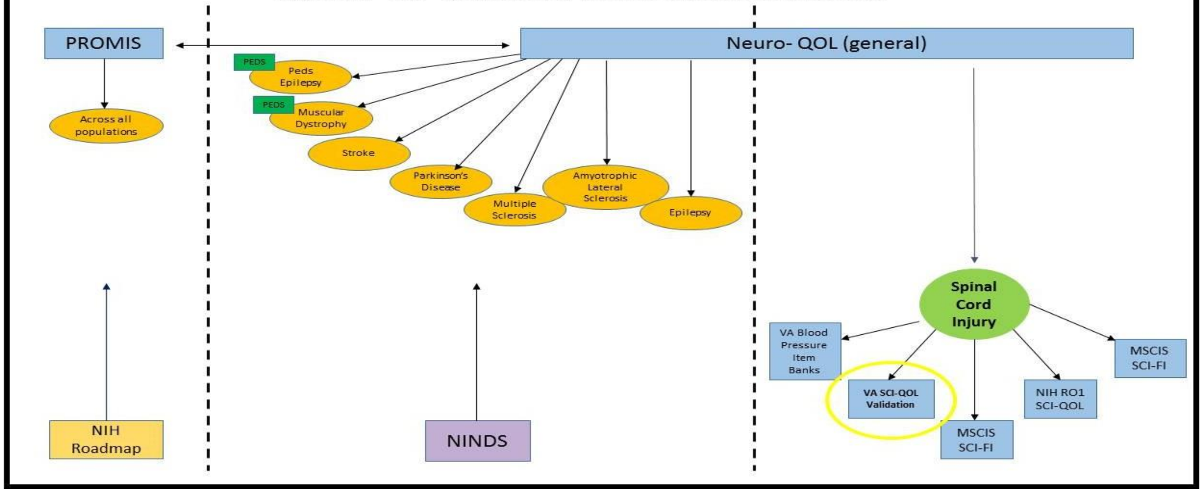

HRQOL measurement tools now enhance the efforts of clinicians and researchers who are

investigating interventions for improved function and quality of life in persons with SCI [59, 62-68]. In 2011, Dr. Spungen received VA RR&D, Merit Review funding (#B7566-R) to add Veterans with SCI to the SCI-QOL database. This VA Merit Review study is in its last year of data collection. A graphic depiction of the PROMIS, Neuro-QOL and SCI-QOL is provided (Figure 3).

Psychometric Properties of the SCI-QOL/SCI-FI: Each of the final SCI-QOL/SCI-FI item banks is a unidimensional set of items (determined by confirmatory factor analysis) that have been calibrated with item response theory (IRT). The stability of the IRT calibrations provides preliminary evidence of the SCI-QOL's validity [60]. Furthermore, all SCI-QOL banks demonstrate internal consistency (Cronbach's alpha) and 2-week test-retest reliability. Finally, the SCI-FI (physical functioning) banks have demonstrated responsiveness to the naturally occurring change following SCI at both 6 month and 1 year time points.

The PROMIS Sleep Disturbance outcome measure was validated and calibrated using classic validation techniques and IRT in more than 2,200 adults from an internet pooling sample and medical, psychiatric and sleep clinics [69, 70]. Moderate to high correlations were demonstrated between existing sleep scales and by statistically significant differences found between patients with known sleep disorders and those without any [69]. The PROMIS Sleep Disturbance outcome measurement was found to be sufficient because no unique questions that required SCI-specific content were thought to be needed [66]. The PROMIS Sleep disturbance outcome measure is a useful tool as is.

A clinically significant difference of the patient-reported outcomes for the QOL measurement determined from any of the PROMIS, Neuro-QOL, SCI-QOL, or SCI-FI assessment tools has been undergoing evaluation. In the interim, three prominent investigators in this field joined forces to determine a consensus of a clinically relevant change for any of these patient-reported outcome questionnaires [71]. These investigators support a ½ standard deviation (SD) as a conservative estimate for a clinically meaningful change. A minimally important difference (MID) is expected to be below a ½ SD. They report that, in lieu of a specifically tested effort to determine the MID in a specific population (such as SCI), use of the ½ SD is a strong and conservative approach [71].

In addition to a scientific rationale (please refer to the pilot data), there are ethical and financial considerations that support the justification for the VA to fund this initiative. It has been long recognized that a sedentary lifestyle is deleterious for one's health, self-esteem and well-being.

The earliest survivors of an amputation had at least a wooden peg to assist them with ambulation. Fortunately, prosthetic limb technology has improved such that lower limb prostheses are sophisticated, computerized devices that permit an eligible amputee to walk, run, jump, climb stairs, swim, and perform almost all functions of ambulation and lifestyle activities that they were able to do prior to limb loss. The problem of restoring ambulation to someone with paralysis from SCI has been far more challenging. However, technology has finally advanced sufficiently to address the problem, and although not on par with the prosthetic limbs yet, exoskeletons offer an upright ambulatory solution for persons who are paralyzed and potentially other wheelchair users for mobility. The exoskeletal-assisted walking technology represents a paradigm shift for Veterans with chronic SCI who only have had the option of the wheelchair for "ambulation" as standard care. This new paradigm for mobility and rehabilitation for our Veterans with chronic SCI has the obvious and distinct potential to improve their quality of life. In the absence of a medical contraindication, rehabilitation clinicians would never consider the option of not prescribing a lower limb prosthetic to an eligible amputee. Should not a similar decision-making process be applied to our eligible Veterans with SCI?

The DVA has 26 SCI Services that annually care for about approximately 26,000 of the estimated 42,000 eligible Veterans with SCI (VA Fact Sheet, 2009). What if only 1,000 of these Veterans with SCI are eligible and interested in being prescribed an exoskeleton for home/community use? The cost of purchasing 1,000 ReWalk exoskeletal units for Veterans with SCI would be about $78 million dollars, not including the clinical staff needed for training and monitoring the use of this device. However, if 10,000 Veterans with SCI wanted and were eligible for one of these devices, then including the support services, this would be approaching a billion dollar item for the DVA. Is it not prudent to study this device in the home/community environment to determine its safety and efficacy prior to being pressured to embark on this path by Veteran, public, or Congressional influence? Further developments in exoskeletal technology and new products are inevitable. Of note, however, the current state-of-the-art exoskeletal technology is not expected to change in the time-frame of this proposed study. In addition, this study is uniquely designed as a template that would support any new exoskeletal developments. It would benefit the VA to have a system in place for the assessment of this particular robotic device because the ReWalk is the first and only such device to date to receive FDA Class 2 approval for institutional and home/community use. Such an initiative could then provide the structure and generate the necessary experience to evaluate future exoskeletal technologies.

Because the ReWalk exoskeleton is a radical and exciting departure from the traditional

rehabilitation approaches, by permitting those with SCI to stand and walk overground, there has already been a fair amount of media and publicity on the exoskeletal-assisted walking technology. This technology is already highly visible in the public eye. The VA would be making a clear and unequivocal statement of its intent to improve the lives of Veterans with SCI by testing the efficacy of this device in the home and community environments.

B. Pilot Data

To date, 19 participants with SCI who were wheelchair users for indoor and outdoor mobility were consented and screened for eligibility. Seven of the 19 participants were screening failures (two for low bone mineral density (BMD), one for medical issues, and four for scheduling conflicts) and 12 completed 15 or more EAW sessions. Pre- and post-walking data were collected in all 12 participants. One of 12 participants was lost to follow-up for the QOL data after 15 sessions (the participant had been wait-listed for medical school and was suddenly accepted, necessitating abrupt withdrawal from the study); as such, walking data are reported on participants (Table 1). QOL data are reported on 11 participants (below in the section for the Primary Outcome 2). The participant that was lost to follow-up for the QOL data was treated as study failure and was included as such for the power calculations. Of the 12 participants who were trained in the exoskeletal device, compliance with attendance to the sessions was >90%. If a session was missed, make-up sessions were easily scheduled, often at the request of the participant. The demographic data is reported in 12 participants with durations of injury ranging from 1 to 19 years (Table 1). The best walking tests achieved with the level of assistance (LOA) needed for this activity are also reported (Table 1.)

Table 1. Characteristics of the Participants and Walk Test Results

Demographic Characteristics								Walk Tests (WT) and Levels of Assistance (LOA)				
SID	Age (y)	Ht (cm)	Wt (kg)	Gender	DOI (y)	LOI	AIS	10 m WT (s)	10 m WT (m/s)	6-min WT (m)	6-min WT (m/s)	6-min WT (LOA)
**1**	34	173	66.7	Male	9	T4	B	39	0.256	90.2	0.251	Min
**2**	48	168	68	Male	4	T10	A	62	0.161	50.5	0.14	Min
**3**	44	183	77.1	Male	4.5	T4	A	20	0.58	209.0	0.581	MI
**4**	58	160	64.4	Female	1.5	C8/T8	A (NT)	24	0.417	139.0	0.386	MI
**5**	61	175	72.6	Male	14	T11	A	23	0.435	137.4	0.382	MI
**6**	24	185	74.8	Male	5	T5	A	56	0.179	60.2	0.167	Min
**7**	40	183	88.5	Male	1.5	T1	B	61	0.164	50.8	0.141	S
**8**	56	175	83.9	Male	3	T9	A	22	0.459	151.0	0.419	S
**9**	50	183	99.8	Male	11	T7	A	17	0.585	208.2	0.578	MI
**10**	37	170	65.8	Male	6	T2	A	22	0.459	150.0	0.417	Min
**11**	64	173	72.8	Male	3	T2	A	78	0.128	46.3	0.129	Mod
**12**	37	152	65.8	Female	19	C8	C (NT)	14	0.714	255.9	0.711	MI

Table 1. SID=subject identification number; y=years; cm=centimeters; kg=kilograms; DOI=duration of injury; LOI=level of injury; AIS=American Spinal Injury International Standards Scale; LOA=level of assistance; s=seconds; m=meters; and NT=non-traumatic SCI. LOA was adapted from the FIM as one of the following: moderate assistance (Mod) – participant performs 50% to 74% of the task and the trainer has both hands on the participant or device at all times to provide occasional guidance or balance support; minimal assistance (Min) – the user performs 75% or more of the task and the trainer has one hand on the participant or device for infrequent guidance or balance support; supervision (S) – the trainer is not touching the participant but is close enough to reach in to provide support for balance or guidance as needed; and modified independence (MI) – the trainer does not provide any assistance, and the participant is fully independent while walking in device. Nineteen participants were consented for screening eligibility. Seven participants (SID#'s 2, 3, 4, 8, 12, 13, and 17) were screening failures: two for low BMD, one medical illness, and four for schedule conflicts.

<u>Heart rate (HR) and blood pressure (BP):</u> HR and BP responses were measured during each session in the resting seated position prior to standing and walking, immediately after walking while still standing and then again when seated, after the walking session. The results at 18±5 and 32±18 sessions are reported (Table 2). The vital sign responses during sitting and post walking were in the expected ranges, demonstrating an increase in HR from seated to walking. In the participants studied, there were no orthostatic hypotensive episodes with the postural changes from sitting to standing in the device.

Rating of perceived exertion (RPE): RPE is a self-reported indicator of how hard the person perceives that they are performing an activity. RPE correlates well with HR when a 0 is added to the rating value (for example, a RPE of 6 is equivalent to a HR of 60 bpm, a typical resting value; and a RPE of 20 is related to a maximal effort of a HR of 200 bpm [72, 73]. As the participants trained, more actual walking occurred during each session, yet their perception of exertion during walking was reduced (Table 2).

Table 2. Group Average Heart Rate, Blood Pressure and Rating of Perceived Exertion Responses to Exoskeletal-Assisted Walking across Sessions

Sessions	RPE	Heart Rate (bpm)		SBP (mmHg)		DBP (mmHg)	
	Standing (post EAW)	Seated (pre EAW)	Standing (post EAW)	Seated (pre EAW)	Standing (post EAW)	Seated (pre EAW)	Standing (post EAW)
1 to 10	14±2	79±13	102±22	127±12	130±14	72±8	73±10
11 to 25	12±3	80±9	112±22	123±15	122±20	73±13	68±20
26 to 40	10±3	75±11	113±17	122±19	123±17	68±9	70±8
> 40	8±2	79±13	114±25	128±13	127±17	72±9	72±2

Table 2. SBP=Systolic blood pressure; DBP=Diastolic blood pressure; mmHg=millimeters of mercury; EAW=exoskeletal-assisted walking

C. Preliminary Data Employed for the Primary Outcome Variables

Primary Outcome 1 (supporting data): A commonly used measure of physical and mental patient-reported health outcomes is the SF-36 [74]. The SF-36 has been reported in persons with SCI of less than 6 months post injury [75] and in those who were less than four years post injury [76]. In the Veterans Health Administration, the Veterans Rand 36 (VR-36) has been used to document health-related QOL outcomes in Veterans [77-79]. The SF-36 was administered to participants before (N=12) and after (N=11, one lost to follow-up) EAW training. Using the MCS scores to calculate a mean difference and 95% CI for the upper and lower limits for the pre- and post- values, the average value for a net change demonstrated clinically significant improvement of 9.03 ± 17.02, CI -2.4 to 20.5, p=0.1087 (Figure 4). **Four of 12 participants demonstrated a clinically relevant change of ≥4.0 on the MCS of the SF-36.** This proportion was used to calculate the power and sample size for the Primary Outcome 1 (MCS/VR-36) that is reported in the statistical section of the proposal. [Note, the MCS SF-36 was used for the preliminary study, but the MCS/VR-36 will be used in this study as the Primary Outcome 1.] In support of the average improvement in the MCS scores, comparisons were made with those of other studies where the SF-36 was administered to persons with SCI within 6 months of injury [75] and then in another group who were less than four years since injury [76]. The MCS average scores for the pre- and

post- ReWalk participants were compared to data from both studies. Lucke, et.al., who administered the SF-36 to persons with SCI of only 6 months duration [75] and Westgren et.al. who administered the SF-36 in those with SCI of <4 years duration [76] (Figure 5). Both of these studies reported MCS scores of 51.8 and 51.9 respectively; the MCS scores from these prior reports are consistent with the findings of our 11 participants at baseline (MCS of 52.3). After EAW, the ReWalk participants increased to 61.5 (Figure 5).

Figure 4. Average Pre and Post Results from the SF-36 Mental Component Summary

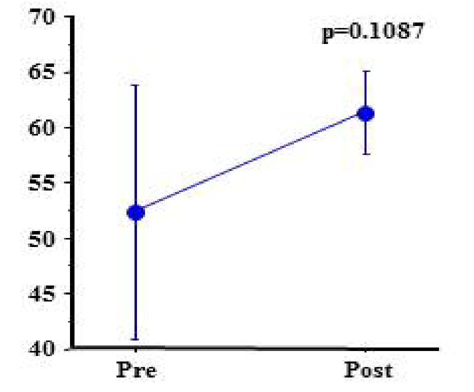

Primary Outcome 2 (supporting data): Three item banks from the SCI-QOL Physical-Medical Health domain were used to assess pre- and post- changes after 40±15 sessions of exoskeletal-assisted walking in 11 participants (one of the 12 was lost to follow-up for the QOL assessments). These item banks consisted of patient-reported outcomes for bladder management difficulties, bowel management difficulties, and pain interference. The group means for the pre-and post-values with the 95% CI are presented (Figure 6). Participants reported significant improvement (indicated as a reduction in scores) in the bladder (Figure 6A) and bowel (Figure 6B) item banks. Pain Interference also improved but failed to reach statistical significance (Figure 6C). In support of the SCI-QOL net pain improvements, analysis of the Physical Pain and Pain interference components from the SF-36 demonstrated significant net improvements from 50±24 to 76±12, p=0.0253. Using a simple sum score of the three SCI-QOL item banks (bladder, bowel and pain) from the Physical-Medical Health (PMH) domain, the mean difference from pre- to post-EAW training was -27.1±34.3; 95% CI: -50.1 to -4.0, p=0.0257. **Five of the 12 participants**

Figure 5. SF-36 Mental Component Summary

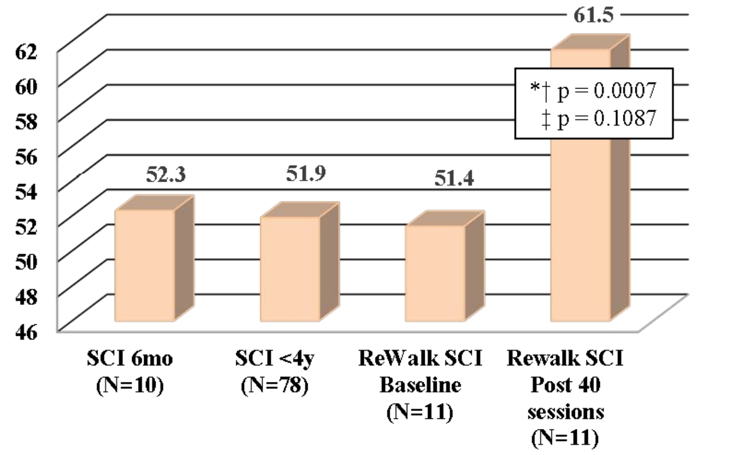

Figure 5. *SCI 6mo data from [64] vs. ReWalk Post; †SCI<4y [65] vs. ReWalk Post; ‡ReWalk Pre vs. Post.

demonstrated a clinically relevant improvement of at least 10% in the sum score of the three item banks. This proportion, 42% responders, was used to calculate the power and sample size for the Primary Outcome 2 (PMH/SCI-QOL) that is reported in the Statistical Analysis Plan (Section XIII) of the protocol.

Figure 6. Pre and Post Results for the SCI-QOL Bladder (A), Bowel (B) and Pain (C) Item Banks

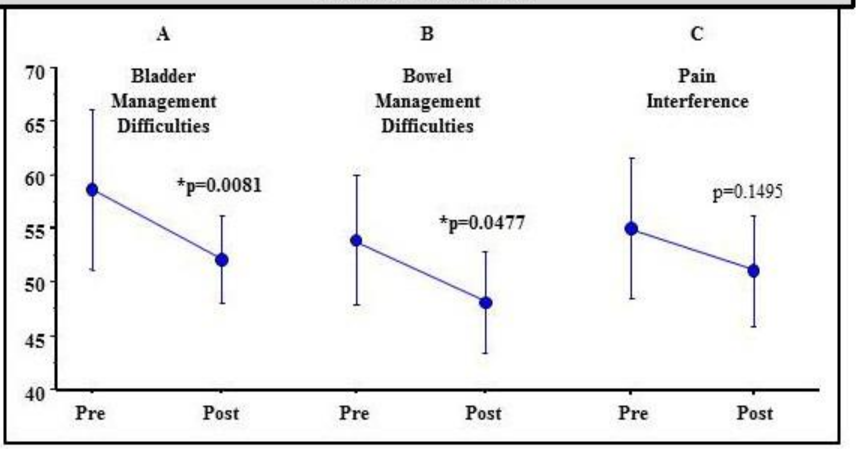

D. Supporting Data for the Major Secondary Outcome Variable

Body composition for fat and lean tissue mass was measured by the soft tissue components of a dual energy x-absorptiometry (DXA) scanner in the 12 participants of the pilot study. Six of 12 participants lost more than 1.0 kg of total body fat (-2.54±0.88 kg). Two lost less than 1.0 kg and 2 gained more than 3.0 kg of total body fat. One participant was lost to follow-up. The loss of ≥1.0 kg of total body fat mass in one half of the participants is a remarkable finding because few efforts at an intervention (i.e. physical activity or dietary) have been successful in reducing fat mass in this population (Table 3). **Six of 12 participants achieved a meaningful reduction in total body fat mass (≥1.0 kg loss). It is anticipated that during the four-month home use phase, 35% of the Intervention group will maintain a ≥1.0 kg fat loss from baseline. This proportion (35%) was used to calculate the power for the major secondary outcome.**

Table 3. Preliminary Data for Total Body Fat (kg) (For the Major Secondary Outcome)				
**S's**	**Pre (kg)**	**Post (kg)**	**Diff (kg)**	**≥1.0 kg loss**
**1**	9.71	6.98	-2.73	yes
**2**	15.43	14.13	-1.30	yes
**3**	31.77	30.78	-0.99	no
**4**	24.22	21.77	-2.45	yes
**5**	25.84	25.06	-0.78	no
**6***	16.15			no
**7**	29.35	26.21	-3.14	yes
**8**	32.98	29.23	-3.74	yes
**9**	31.75	29.89	-1.87	yes
**10**	16.20	15.82	-0.38	no
**11**	21.29	24.76	3.47	no
**12**	34.88	38.50	3.62	no
			**Responders**	**6**

* Subject 6 was loss to follow-up, but was treated as a study failure in the preliminary data.

E. Secondary Outcomes

The PROMIS Sleep Disturbance outcome measure was used to assess change in sleep disturbance. Additional measurements of sleep and fatigue problems were assessed using the Epworth Sleepiness Scale [80] and the Fatigue Severity Scale [81, 82]. These tools are measures of daytime or wake-time sleepiness and tiredness. Net improvements were noted in all three surveys, with statistically significant results in the PROMIS Sleep Disturbance outcome measure (Figure 7).

Figure 7. Pre and Post Results for the PROMIS Sleep Disturbance

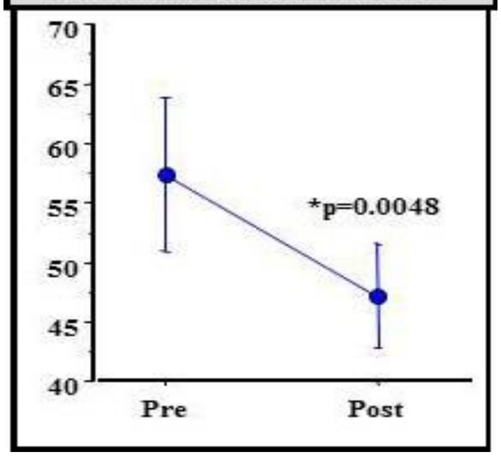

Bowel function was also measured by two self-reported assessment tools (Bristol Stool Scale and Bowel Survey) before and after 20 to 30 sessions of ReWalk training. Developed by Lewis SJ and Heaton KW in 1997, the Bristol Stool Scale (Scandinavian Journal of Gastroenterol. 1997 Sep;32(9):920-4) is an indicator of stool consistency. Participants reported an overall softening of their stool with values, on average, reaching a "desirable" stool consistency after ReWalk training, and this finding was consistent with a reduction or discontinuation of the use of bowel medications.

The Bowel Survey accounts for important items, such as daily time spent for a bowel evacuation (BE), frequency per week of BEs, monthly frequency of incontinent episodes, the amount of medication, mechanical, digit, and/or flushing extraction methods used to achieve a BE. After 2

to 3 months of EAW at 4 to 6 hours per week, the group reported reduced time spent daily for a BE, increased BE frequency per week and reduction or discontinuation of the amount of BE medication and/or mechanical methods used (Table 4).

Table 4. Bowel Function Changes Pre and Post EAW Training

	Frequency of Bowel Evacuations (#/week)		Time spent per Bowel Evacuation Day (minutes/day)		Bowel Accidents (# in past month)		Laxative, Stool Softener, Enema, and/or Digital Stimulation Use
S's	Pre	Post	Pre	Post	Pre	Post	Change
1	1 to 2	3 to 4	60 to 180	15 to 30	1 to 2	0	Reduction in enema and digit stimulation
2	1 to 2	3 to 4	15 to 30	15 to 30	0	0	Discontinuation of laxative, reduction in enema and digit stimulation
3	2 to 3	3 to 4	60 to 180	30 to 60	5 to 6	0	Discontinuation of laxative and stool softener
4	1 to 2	3 to 4	60 to 180	30 to 60	≥ 7	0	Reduction in use of laxative, stool softener, enema, and digit stimulation
5	5 to 6	5 to 6	60 to 180	30 to 60	1 to 2	0	Discontinuation of laxative and supplemental fiber
6	3 to 4		30 to 60		3 to 4		Lost to follow-up
7	≥ 7	5 to 6	60 to 180	30 to 60	1 to 2	0	Reduction in use of laxative, stool softener, enema, and digit stimulation
8	3 to 4	3 to 4	15 to 30	15 to 30	3 to 4	1 to 2	Reduction in use of laxative, stool softener, enema, and digit stimulation
9	3 to 4	≥7	15 to 30	15 to 30	0	0	Reduction in use of laxative and stool softener
10	≥ 7	5 to 6	60 to 180	60 to 180	3 to 4	3 to 4	No change
11	3 to 4	3 to 4	30 to 60	30 to 60	0	1 to 2	Laxative and digital stimulation use increased, No change in enema use
12	≥ 7	5 to 6	5 to 15	5 to 15	0	0	No change

Summary of Pilot Study: The pilot EAW study using the ReWalk performed at the JJPVAMC trained 12 participants in the ReWalk exoskeleton for a combined total time of >1200 hours. EAW of four to six hours per week was well-tolerated by the participants. Time in the exoskeleton resulted in improved skill level. One-half of the participants reported clinically significant improvements in bladder and bowel function and reduction in pain outcome measurements. There were no study-related serious adverse events (SAEs). Skin abrasions at the points of contact with the device occurred across 9 of the 12 participants; these were reported as "study-related adverse events." All skin abrasions were resolved with adjustments in padding and fit of the device to the participants. One participant fell due to slipping on a wet floor but was not injured. These adverse events were consistent with those reported by others for exoskeletal-assisted walking [43, 44, 83].

II. OBJECTIVES

The short-term aims are to demonstrate in Veterans with SCI who are wheelchair users for indoor and outdoor mobility the efficacy for changes in QOL, improvements in body composition and the safety of home/community use of a powered exoskeleton for ambulation.

The long-term aims are to share the knowledge gained from this research study with the Department of Veterans Affairs (DVA) Veterans Health Administration (VHA) Spinal Cord Injury Patient Care Centers' clinical staff about the training procedures, education of and the development of guidelines for the clinical prescription of the exoskeletal-assisted walking device for home/community use in Veterans with SCI.

The study sample size is powered from preliminary data for the two primary outcomes [Mental Component Summary (MCS) of the Veterans Rand-36 (VR-36) and Spinal Cord Injury Quality of Life (SCI-QOL) assessment tool] and the major secondary outcome (total body fat mass). The secondary outcomes are exploratory and will not be used to power the study sample size.

A. The **Primary Objectives** are to demonstrate that Veterans with chronic SCI of ≥six months duration who are medically stable and are wheelchair users for indoor and outdoor mobility as their standard of care (SOC) plus use of an exoskeletal-assisted walking device in their home and community environments will have clinically meaningful net improvements in the MCS/VR-36 and in patient-reported outcomes for the SCI-QOL bladder, bowel, and pain item banks compared with those who use only SOC for home and community mobility. The primary outcomes to be assessed will be the MCS value and the sum T-score of the SCI-QOL bladder management difficulties, bowel management difficulties and pain interference item banks.

B. The **Major Secondary Objective** is to demonstrate that participants who use the exoskeleton in addition to SOC will have at least a 1.0 kg loss in total body fat mass by the end of the four-month Intervention phase.

C. The **Secondary Objectives** are to demonstrate that participants who use the exoskeleton in addition to SOC will have greater net improvements than the participants who receive only SOC on the following outcomes:

1. Global Impression of Change Scale (Participant- and Companion-rated).

2. Disturbed sleep as measured by the T-score of the Patient Reported Outcomes Measurement Information System (PROMIS) Sleep Disturbance (short form).
3. Self-reported methods and measures of bowel function for:
    a. frequency of bowel evacuation episodes, time per episode, number of self-reported "natural" bowel movements, the amount of bowel evacuation medications used (e.g., laxatives and/or stool softeners), frequency of enemas used, frequency of digital stimulation needed per week, stool consistency (by the Bristol Stool Scale), and frequency of bowel incontinence/accident episodes;
4. Sum T-score of the SCI Functional Index (SCI-FI) physical function short forms (basic mobility, self-care, fine motor, ambulation, wheelchair mobility, and assistive technology);
5. Sum T-scores of the SCI-QOL Emotional domain (Separate T-scores for: 1) anxiety, depression, grief-loss, trauma, and stigma item banks, and 2) positive affect and well-being, self-evaluation, and resilience item banks) (short form);
6. Sum T-score of the SCI-QOL Social Participation domain (ability to participate in social roles and activities, satisfaction with social roles and activities, and independence) (short form);
7. Lipid Profile for high density lipoprotein cholesterol (HDL-c), low density lipoprotein cholesterol (LDL-c), triglycerides (TG), and total cholesterol (TC);
8. Fasting plasma glucose (FPG) and insulin (FPI) levels for calculation of Homeostasis Model of Assessment-Insulin Resistance (HOMA-IR).

D. Hypotheses

Hypotheses are described for the Primary and Major Secondary Outcomes. In Veterans with chronic SCI:

1. <u>Primary Hypotheses (a and b):</u>
    a. 33% of the Intervention group compared with 10% of the Control group will demonstrate a clinically relevant change of ≥4.0 point improvement for the MCS/VR-36 for greater vitality and social functioning, and improved role-emotional and mental health from baseline to the end of the Intervention phase.
    b. 42% of the Intervention group compared with 10% of the Control group will demonstrate a clinically significant change of 10% improvement from baseline to the end of the intervention phase on the sum T-score of the patient-reported

outcomes from the combined SCI-QOL item banks for bladder management difficulties, bowel management difficulties and pain interference.

2. Major Secondary Hypothesis: It is anticipated that during the Training phase, at least 50% of the Intervention group will experience a total body fat mass loss of ≥1.0 kg from baseline to the end of the 25±5 sessions. During the home/community use portion of the Intervention phase it is hypothesized that 35% of the total Intervention group will demonstrate at least maintenance of the ≥1.0 kg loss after four months of in-home use of the exoskeleton. As such, 35% of the Intervention group will demonstrate a loss of ≥1.0kg of total body fat mass by the end of the Intervention phase relative to baseline values compared with 10% of the Control group.

III. EXPERIMENTAL DESIGN

Research Design: A two-group (Intervention and Control), randomized, clinical trial will be performed with a one-year feasibility component. The Intervention group will consist of SOC plus exoskeletal-assisted walking (EAW). The Control group will consist of SOC only. The study will have two phases: Screening and Intervention. Fifteen VA SCI Services will participate as study sites. The study will require seven years in total to complete. A feasibility phase will be employed using a staggered start with six sites starting initially, four sites starting one year later, and five sites starting two years later. These initial six sites will be used to assess the start-up activities (hiring, training, equipment procurement, CIRB paperwork, etc.), the rate of recruitment and any other issues that may be of value for the successful completion of the study. Lessons learned will be implemented for the remaining sites.

A. Screening Phase

The Screening phase consists of pre-screening, consenting process, screening evaluations, baseline evaluations of the study outcomes, basic EAW training with a skills test, and randomization. During *pre-screening*, the potential participants will be first contacted by the Site Investigator, who is a SCI staff physician, through chart reviews, other physician referrals, pre-existing knowledge about their patients, access to contact information in a VA Informatics and Computing Infrastructure (VINCI) database, physician referrals at other VA hospitals, and/or through study flyers and study invitation letters. Study flyers may also be given to potential participants in the SCI Clinic, posted in local area hospitals, or distributed at local and national SCI events. Study

flyers may also be posted generally in areas where potential participants may see the information (i.e. local chapters of Veterans Organizations, SCI sporting events) and included in newsletters or journals with a focus on the SCI population. Each site is expected to prescreen at least 100 potential participants. Pre-screened eligible and interested potential participants will be referred by the Site ReWalk Trainers and/or Site Coordinators for initiation of the consenting process and scheduling of the screening evaluations. The *screening evaluations* will be performed only in consented participants by the Site Investigator, other Site Physician(s) and/or the site study team. These screening evaluations are for the inclusion/exclusion criteria for medical, physical and health-related eligibility. The screening evaluations include a history and physical examination (H&PE), a specific history of fragility fractures, unhealed traumatic fractures, swelling, bruising, redness, or other abnormalities of the lower extremities, International Standards for Neurological Classification of SCI (ISNCSCI) examination for neurological level and function [formerly called American Spinal Injury Association (ASIA) or American Spinal Injury International Standards Scale (AIS) examination], a BMD scan and a lateral foot x-ray of each calcaneus with potential for a clinical referral for a CT scan for further evaluation, if recommended. Participants who pass the inclusion/exclusion criteria will then undergo a home evaluation and companion identification for continued eligibility. The potential participant may have up to three companions who share in the EAW training with the participant. All companions will be provided a separate consenting process and will either sign an informed consent form or assent over the phone to be enrolled. Baseline evaluations will be performed only in those participants who have passed all screening criteria to this point. Once the baseline evaluations are completed, all participants will be fitted for the ReWalk and begin a five-session basic EAW training course. In order to avoid a biased group selection, all participants will be trained for five sessions of EAW basic skills. The EAW basic skills training will be conducted at the VA hospitals at fifteen sites, in the Community Based Outpatient Centers (CBOCs) under the same Federal Wide Assurance (FWA) as the sites, or in the participant's home/community. Only those who pass the EAW basic skills test (and all other screening criteria) will be eligible to be randomized to either the Intervention or Control group. Each site is expected to consent and screen at least 60 potential participants and randomize 4-24 participants for an overall 160 randomized across 15 sites.

Once randomized, the Intervention group will participate in 25±5 sessions of EAW Training and the Control group will participate in Orientation. The EAW training will be conducted at the VA hospitals at fifteen sites, in the CBOCs under the same FWA as the sites, or in the participant's home/community. During Orientation (while the Intervention group is being trained on the ReWalk), the Control group will attend weekly meetings at the study site or be contacted by phone

interview or secure messaging using My HealtheVet for review of their regular activities, which will serve as an “attention” balance to the Intervention group. Participants in the Intervention group will be required to pass the EAW Advanced Skills Test at the end of the 25±5 sessions of EAW Training prior to taking the device home. The companion is also required to pass part of this Advanced Skills Test. Participants (and their companions) who fail the EAW Advanced Skills Test will not be able to take the ReWalk home and will be counted as study “failures”. (Note: the PI and her team have observed that persons with SCI who are able to learn the basic standing and weight shift balance skills within five sessions, generally, also learn the advanced walking skills for home use within 20 to 30 sessions. As such, in order to screen out those participants who are likely to fail the EAW Advanced Skills Test for home use, the five-session testing criteria will be used for all participants before randomization.)

A study form listing the number of participants for Pre-screening, Screening evaluations, five-session EAW training, and continuation (or not) to randomization will be recorded. The reasons for pre-screening and screening failures will be recorded.

B. Intervention Phase

After passing the EAW Advanced Skills Test at the end of the 25±5 sessions of EAW Training, the Intervention group will take the ReWalk home for four months. The Intervention group will continue to participate in wheelchair or other non-exoskeletal, non-wheelchair SOC activities as usual, but will also use the ReWalk in their home/community environment as per the specific recommendation by the study team during the home set-up. The Control group will participate in four months of SOC only, defined as usual wheelchair use (or other non-exoskeletal, non-wheelchair activities). Both groups will be required to complete a weekly Usual Activity Log. Participants in the Intervention group will additionally have the number of steps taken recorded from the step counter that is built-in to the ReWalk device, and the location and time of the EAW activities in an EAW weekly log. Site team members will contact participants in both groups on a weekly basis, either over the phone or through secure messaging using My HealtheVet to review the Usual and EAW Activity Logs and to identify problems or issues that may present. Participants in both groups will be encouraged to contact site team members with any questions pertaining to the study at any time. The Intervention and Control groups will receive the same pre- and post-outcome assessments and similar amounts of contact and attention from the study team during the Intervention phase. Participants in both groups will return to the site for an Intervention phase assessment of the outcome tests at month 2 and again at the end of month 4 (primary outcome

assessment time point). Across all study sites, 160 participants are to be randomized. Each site is expected to randomize between 4 and 24 participants. Adverse events will be recorded in both groups.

C. Post Study Participation for the Control Group

In order to avoid potential participants not being interested in the study because of the 50% chance of not receiving the intervention, the Control group will be offered a two-month EAW training program after completion of the study protocol as an outpatient. Outcome data will not be collected during this outpatient, EAW post-study training for the Control group. The study site team will provide the EAW training to the former Control group participants during this phase and adverse events will be recorded during this post study component for the Control group. After participation in the study, participants may be considered for home use prescription of the exoskeleton by the clinical SCI Service.

D. Study Duration and Timeline

CS #2003 is projected to be a seven-year study. A staggered start approach will be employed with six sites starting during the first year of the study, four sites starting one year later, and five sites starting two years later. The six sites will have a one-year start-up, four years of enrollment and an additional one year of data collection and study closeout. The four sites will follow the same plan, but starting one year later with three years of enrollment. The remaining five sites will start two years later and have two years of enrollment and one year of data collection and closeout. All 15 sites will complete data collection and study closeout in the sixth year of the study. An additional year for the Coordinating Center and Chair's Office is planned for data analyses and write-up (seven years in total). During the 6-week pre-start-up period, the Chair's Office will begin staff hiring procedures by providing position description (PD) postings for the sites and submit the protocol to the Central Institutional Review Board (CIRB) and local Research & Development (R&D) for review. The CSPCRPCC will submit the investigational device exemption (IDE) application to the FDA. A year will be needed for study start-up activities for the sites CIRB and local R&D submissions, ordering of the equipment (ReWalks and iDXA scanners), site staff hiring, and the ReWalk and study protocol training. During the enrollment years 1-4, participants will be prescreened, screened, and randomized. Year five will be used for continuation of data collection; no new enrollment will occur in year six. Data edits will be on-going throughout the five years of data collection. Year six will be needed for general wrap-up and

closeout at the study sites. During Year 7, data analyses and manuscript writing will be performed. A detailed description may be found in the “Study Timeline” (Table 5).

Participants in the Intervention group will require eight to nine months to complete the study. Participants in the Control group will require seven to nine months for study completion. The extra time is for the optional Control EAW in-hospital training sessions after the study is completed. This amount of time breaks out as follows. Once consented, potential participants will need one week for the screening evaluations, one week for the baseline outcome evaluations, and two weeks for the device fitting and five-session basic skills training and skills test. The Intervention group will go through a three-month Training phase, which includes the additional 25±5-sessions of supervised EAW training with their companion. After randomization, the Control group will participate in a two-month Orientation phase. The Intervention phase will require four months for both groups. The post evaluations will require one week. Those in the Control group will have the opportunity to participate in an additional two-month optional EAW outpatient training without data collection. A detailed description may be found in the “Training and Testing Schedule” (Table 6).

Table 5. Study Timeline	Funding notification (8/ 6-week Pre Start-up (no study costs)	Start-up				Y1				Y2				Y3				Y4				Y5				Y6			
						Enrollment & Participant Data Collection (160 Participants to be enrolled by end of Q4 Y4)																Study Closeout				Data Analyses and Manuscript Writing			
		FY16				FY17				FY18				FY19				FY20				FY21				FY22			
		Start-up Year (6 Sites)				Enrollment Year 1 (6 Sites) Start-up year (4 Sites)				Enrollment Year 2 (6 Sites) Enrollment Year 1 (4 Sites)				Enrollment Year 3 (6 Sites) Enrollment Year 2 (4 Sites) Start-up & Enrollment Year 1 (5 Sites)				Enrollment Year 4 (6 Sites) Enrollment Year 3 (4 Sites) Enrollment Year 2 (5 Sites)				Participant Carry-over and Study Close-out (All 15 sites)				Study analyses and write-up			
		Q1	Q2	Q3	Q4	Q1	Q2	Q3	Q4	Q1	Q2	Q3	Q4	Q1	Q2	Q3	Q4	Q1	Q2	Q3	Q4	Q1	Q2	Q3	Q4	Q1	Q2	Q3	Q4
Administration start up	x	x																											
Chair's Office CIRB	x	x																											
CSPCRPCC IDE to FDA	x	x																											
Chair/Sites' PDs	x	x																											
Chair/Sites' R&D	x	x																											
Chair's funding received		x	x																										
Chair/Sites' staff hiring		x				x								x															
Sites' CIRB		x	x																										
Equipment contracts		x	x																										
Equipment ships to sites			x	x										x															
iDXA Site training			x	x	x	x	x							x	x														
ReWalk onsite training[1]			x	x	x	x	x							x	x														
ReWalk training follow-up[2]					x	x		x		x		x		x		x		x		x									
Study meetings				x				x				x				x				x			x						
Pre Screening				x	x	x	x	x	x	x	x	x	x	x	x	x	x	x	x	x	x								
Screening					x	x	x	x	x	x	x	x	x	x	x	x	x	x	x	x	x								
Randomization						x	x	x	x	x	x	x	x	x	x	x	x	x	x	x	x								
Data collection/edits						x	x	x	x	x	x	x	x	x	x	x	x	x	x	x	x	x	x						
Study close out																				x	x	x	x	x	x				
Data analyses/writing																								x	x	x	x	x	x

Table 5 Legend. A total of 160 (80/group) participants will be enrolled and randomized across all 15 study sites. CIRB=Central Institutional Review Board (VA); CSPCRPCC =Cooperative Studies Program Clinical Research PharmacyCoordinating Center; IDE=Investigational Device Exemption; FDA=Food and Drug Administration; PD=Position descriptions for hiring staff; R&D=Research and Development Committee (at Sites and Chair's Office); iDXA=DXA scanner for bone mineral density and total body composition. [1]ReWalk Site training is performed by ReWalk Robotics, Inc. as onsite training. [2]ReWalk Site trainig follow-up is performed by the CS #2003 National Coordinator as a follow-up training at the sites.

Table 6. Testing and Training Schedules

Phases:	Screening Phase					Orientation/Training Phase		Intervention Phase					Control EAW
Months:	Month 1					Mo 2 to 4		Mo 5	Mo 6	Mo 7	Mo 8	Mo 9	Mo 9 & 10
Weeks:	1 to 4					5 to 16		17 to 20	21 to 24	25 to 28	29 to 32	33	33 to 40
Pre Screening	Consent Obtained	Screening Evaluations	Pre Evaluations (Baseline for both groups)	5 Session EAW Training	Randomization	Orientation/Training<br>Control Group (Standard of Care Review)<br>Intervention Group (EAW Training)	Orientation/Training Phase Evaluations (Both Groups)	In-Home/Community<br>Control Group (Standard of Care)<br>Intervention Group (Standard of Care plus Home EAW)				Post Intervention Phase Evaluations (Both Groups)	(no Data)
**SCREENING Evaluations**													
**History & Physical Exam**		x											
**ISNCSCI Exam**		x					x					x	
**BMD Scan**		x											
**Bi-lateral foot x-ray**		x											
**CT scan**, if indicated		x											
**Home Evaluation**		x											
**Companion Evaluation**		x											
**OUTCOME Assessments**													
**iDXA Body Composition**			x				x		x			x	
**VR-36**			x				x		x			x	
**SCI-QOL**			x				x		x			x	
**Global Impression of Change Scale**			x				x		x			x	
**PROMIS Sleep Disturbance**			x				x		x			x	
**SCI-FI**			x				x		x			x	
**Bowel Function**			x				x		x			x	
**Blood draw (Lipids/Glu/Ins)**			x				x		x			x	
**QUALITY CONTROL Data**													
**EAW Basic Skills Test**				x			x					x	
**Usual Activity Log**				x		1 x Weekly		1 x Weekly					
**Step Counter** (Intervention group only)				x		Each Session		1 x Weekly				x	
**EAW Mobility Tests** (Intervention group only)				x		Each Session			x		x	x	
**EAW Activity Log** (Intervention group only)				x				1 x Weekly				x	
**EAW Advanced Skills Test**							x					x	
**Home set-up**							x	PRN Home Interim visits					
**Exit survey**												x[1]	x[1]

Table 6. Legend	
**Home and Companion Evaluations**	Home and companion evaluations will be performed on all participants who have passed the medical screening criteria and will be performed prior to randomization to avoid unbalanced or biased groups.
**OUTCOME Assessments**	Outcome assessments will only be performed on those potential participants who have passed all screening criteria. Screening failures and reasons will be recorded on study forms.
**SOC**	Standard of care is defined as wheelchair use and usual lifestyle activities.
**Standard of Care Review**	Participants complete an interview with a study team member to review usual lifestyle activities and Usual activity weekly log.
**EAW Training**	Intervention group attends the exoskeletal-assisted walking training for 25±5 sessions.
**EAW Advanced Skills Test**	EAW Advanced Skills Test is a combined participant/user and companion test. NOTE: Participants and companions must pass the EAW Advanced Skills Test to continue to home use; those who do not are treated as "study failures".
**Home set-up**	Only those participants and their companions who pass the EAW Advanced Skills Test will continue on to home/community use. For these participants, a second home visit for set-up will be performed by a study team member from the Site.
**Exit survey**	Exit surveys may be completed with the Post Intervention Phase Evaluations for both groups or after the optional EAW in-hospital training for the control group
**Abbreviations**	EAW = Exoskeletal-assisted walking; Intervention = Standard of care wheelchair use (i.e. usual lifestyle activities) plus Exoskeletal-assisted walking; Control = Standard of care, wheelchair use (i.e. usual lifestyle activities); ISNCSCI Exam = International Standards for Neurological Classifications for SCI (formerly, ASIA and AIS); BMD Scan = bone mineral density scan; iDXA = Dual energy x-ray absorptiometry scan for fat and lean tissue masses; CT = Computed tomography; SCI-QOL = Spinal Cord Injury Quality of Life patient-reported outcomes tool; PROMIS Sleep Disturbance = Patient Reported Outcomes Measurement Information System component for Sleep Disturbance; VR-36 = Veterans Rand-36; and SCI-FI = SCI Functional Index of patient-reported outcomes for physical function.

IV. PARTICIPANT RECRUITMENT

A. Recruitment and Screening

The Site Investigator (a SCI staff physician) will be responsible for initially approaching each potential participant to be recruited and screened for eligibility. S/he will pre-screen from the medical records, referrals from other SCI physicians, known SCI patients for potential participants, access to contact information in a VINCI database (VA hubs and spokes and VAs geographically near participating local sites), and physician referrals at other VA hospitals. The Site Investigator may send study flyers and study invitation letters to potential participants. Additionally, the study flyers may be given to potential participants in the SCI Clinic, posted in local area hospitals and on local site FaceBook pages, or distributed at local and national SCI events. Study flyers may also be posted generally in areas where potential participants may see the information (i.e. local chapters of Veterans Organizations, SCI sporting events) and included in newsletters or journals with a focus on the SCI population. After the initial contact, by the Site Investigator, the Site Coordinator and/or Site ReWalk Trainers will participate and assist in the recruitment and consenting process. If the individual provides informed consent to participate, s/he will be fully evaluated by the Site Investigator and other appropriate members of the site's study team. Screening evaluations will be performed in all participants. In order to assure that by completion of EAW training each participant is competent to use the exoskeleton in the home/community environment, a five-session basic EAW training with a Basic Skills Test will be conducted on all participants to determine the eligibility of the participant to be randomized. Please note that the inclusion/exclusion criteria are also designed to exclude participants who have weak or absent arm, hand, and/or trunk stability and control, and thus wean out those who are not likely to achieve the necessary skills for home/community use. A pre-approved home evaluation and companion interview will be established and evaluated early in the screening process for all participants. Those who have provided written informed consent and have passed all Screening criteria including the EAW Basic Skills Test will be eligible to be randomized. One hundred-sixty participants (N=160) will be randomized and assigned to either the SOC plus EAW Intervention group (n=80) or the SOC Control group (n=80) across all 15 study sites. Each site is expected to randomize between 4 and 24 participants. Once a participant has been randomized, s/he may not be randomized again.

B. Randomization System

An Interactive Touch Tone Randomization System (ITTRS) will be used for randomization within

each site. This system will be accessed through the Cooperative Studies Program Coordinating Center at the Perry Point VA Medical Center, Perry Point, MD.

C. Population to be Studied

One hundred-sixty male or female Veteran or active duty military personnel with chronic SCI, of ≥six months duration, who are ≥18 years of age, and who are medically stable and wheelchair users for indoor and outdoor mobility, will be eligible for screening to be enrolled in the study. All potential participants will be Veterans or active duty military personnel with SCI who are affiliated with or transferred to one of the fifteen VA SCI Services that will be participating in this study. Study participants will generally be outpatients with the exception of those inpatients who meet the eligibility criteria and are approved by the Site Investigator (e.g., some inpatients may have been admitted for a wheelchair fitting or another non-medical reason). Non-veterans, other than active duty military personnel getting treatment at the VA, will <u>not</u> be eligible to participate. Eighty participants will receive SOC plus EAW (Intervention group) and 80 will receive SOC (Control group). It is expected that no more than 15% of the participants will withdraw early (dropout rate) and this attrition rate is expected to be evenly distributed between the two groups (12 withdrawals/group). One hundred-thirty-six participants are expected to complete the study (68/group).

V. INTERVENTION AND METHODS OF TREATMENT

Standard of care (SOC) is defined as wheelchair use for indoor and outdoor mobility and any other non-wheelchair activities (such as, standing frame use, swimming, arm bikes for cycling, etc.) that the participant may engage in during their usual lifestyle activities. The Intervention group receives SOC activities plus EAW training (25±5 sessions) to develop a skill set to use the exoskeleton, accompanied by their companion, during a four-month trial with the exoskeleton in the participant's home/community environment. The Control group will receive SOC alone. Accurate recording of SOC and EAW activities is an important component of this study. Two activity logs will be obtained: 1) Usual Activity Log and 2) EAW Activity Log. The Usual Activity Log is designed to capture usual lifestyle physical and recreational activities (non-EAW) that the Veteran or member of the military with SCI participates in weekly. Both groups will be instructed to record any type and amount of time of physical or recreational wheelchair or non-wheelchair activity (i.e., standing frame use, swimming, etc.) that is being performed during the course of the two-three month Orientation/Training and four-month Intervention phase of the study. EAW and

Usual Activity Logs with check-off categorical grouping answers for the type and duration of the activity will be provided to reduce participant burden and to provide standardized answers for analysis. The EAW Activity Log will be used by the Intervention group only to record weekly use for location and time spent in the ReWalk device during the training (hospital halls, SCI service, grounds, CBOCs, home/community etc.) and home/community (e.g., sidewalks, local mall, in home, parks, etc.) phases of the study. The number of steps taken in the ReWalk during training and home use will be recorded on a weekly basis from the built-in step counter. Detailed descriptions of the Intervention and Control schedules of testing are provided (Table 6). Information from the activity logs may be given to the Coordinator over the phone, in-person, or through secure messaging, using My HealtheVet.

VI. METHODS OF FOLLOW UP

The Orientation/Training phase is performed in the hospital or in the participant's home/community. Participants in the Intervention group will be required to meet with the study team for 3 to 5 sessions per week for 20 to 30 sessions. Participants who miss sessions will be contacted by the Site's Study Coordinator or ReWalk Trainers for rescheduling. During the Intervention phase, the participants will be using the exoskeleton in their home/community. During this phase, follow-up contact by the study team members with the participants on a weekly basis will be critical for compliance and troubleshooting of any problems. This contact will be necessary to assist the participants with compliance of completion of the Usual Activity Log and the EAW Activity Log and identification of adverse events in the Intervention group. Contact will be made in person, over the phone, or through secure messaging, using My HealtheVet.

During the Orientation/Training phase, the Control group will make weekly outpatient visits and/or receive contact from members of the site's study team on a weekly basis. During the Intervention phase, follow-up contact by the study team members with the participants in the Control group will be at a minimum of one time weekly. This contact will be critical for compliance of completion of the Usual Activity Log and identification of adverse events in the Control group. Site team members will be instructed in the weekly and monthly participant contact procedures for both groups to collect data, encourage compliance, and identify issues. Contact will be made in person, over the phone, or through secure messaging, using My HealtheVet.

Primary and Secondary outcome assessments will be completed at the SCI Centers at the end of month 2 and month 4 of the Intervention phase in both groups.

VII. METHODS OF ASSURING UNIFORMITY OF INTERVENTION

Uniformity of the intervention within and across the sites is critical. Standardization of staff training procedures has been identified for two main areas: 1) EAW components for the home evaluation, companion interview, ReWalk fitting, EAW Basic Skills Test, EAW walking skill progression, EAW walking test evaluations, EAW Advanced Skills Test, and EAW Activity Log; and 2) administration of the outcome assessments for the VR-36, SCI-QOL, Global Impression of Change Scale, PROMIS Sleep Disturbance, Bowel function, and SCI-FI. Onsite training of procedures will be conducted at each site in order to standardize EAW training techniques across the sites. The ReWalk Trainers will be required to demonstrate knowledge and capability to perform the required procedures related to the EAW training and assessments. The Study Coordinator at each site will be responsible for the QOL outcome assessments. This strengthens the study because the staff persons providing the intervention are not the ones performing the outcome assessments.

VIII. DETERMINING ELIGIBILITY

The determination of eligibility for this study has several levels. First, the Site Physician will pre-screen potential participants from the medical records and knowledge of the person to include those with traumatic or non-traumatic, complete or incomplete SCI duration ≥6 months who are ≥18 years old and are wheelchair-dependent for indoor and outdoor mobility.

Second are the Screening evaluations; those potential participants who pass pre-screening and are interested to be consented will begin the consenting process with the Site Physician and site study team. Those who complete the consenting process will undergo a series of screening evaluations that include: a BMD scan, fracture history, lateral foot x-rays of each calcaneus, history and physical examination and ISNCSCI assessment to determine medical wellness, anthropometric compatibility with the device, level and completeness of injury, hand function, ability to have a companion, and a home evaluation for suitability. Female participants will also receive pregnancy testing. The specific inclusion and exclusion criteria are itemized below.

A. Inclusion/Eligibility Criteria

Veteran/Active Duty Participants:

1. Veterans or active duty military personnel who are at least 18 years of age;
2. Traumatic or non-traumatic SCI ≥6 months duration of SCI;

3. Wheelchair-user for indoor and outdoor mobility;
4. Anthropometric compatibility with the device:
    a. Weight <220 lb. (100 kg),
    b. Thigh length between 14 and 19 in (36 and 48 cm),
    c. Shank length between 17 and 22 in (43 and 55 cm);
5. Able to hold the crutches in hands without modifications;
6. Able to have a companion who can attend approximately one-third of the training sessions who will assist them at home and in the community; and
7. Able to provide informed consent.

Companion Participants:

1. Male/female greater than or equal to 18 years of age;
2. Demonstrates understanding of the time commitment to be a companion;
3. The companion and the user are willing to receive training on how to assist the user with learning to use the device;
4. The companion agrees to ensure that the exoskeleton is used with the crutches at all times;
5. Site Physician and study team members must deem the companion physically able to assist the participant with tasks outlined in the exoskeleton skills inventory (i.e. the companion is able-bodied and is physically able to bend, stoop, squat, kneel, etc.)

B. Exclusion criteria

Veteran/Active Duty Participants:

1. Diagnosis of neurological injury other than SCI;
2. Progressive condition that would be expected to result in changing neurological status;
3. Severe concurrent medical disease, illness or condition judged to be contraindicated by the Site Physician;
4. Unhealed or unstable traumatic or high impact lower extremity fracture (definition below) of any duration that is in the clinical judgement of the study physician to be exclusionary for standing and walking;
5. Knee BMD < 0.60 gm/cm^2;
6. Total hip BMD T-scores < -3.5;
7. Fragility, minimal trauma or low impact fracture of the lower extremity since spinal cord injury (definition below);
8. Untreatable severe spasticity judged to be contraindicated by the Site Physician;
9. Flexion contracture > 15º at the hip and/or > 10º at the knee;

10. Limitations in ankle range of motion that cannot be adapted with an orthotic device (plantar flexion > 0^0);
11. Untreated or uncontrolled hypertension (systolic blood pressure >140 mmHg; diastolic blood pressure >90 mmHg);
12. Unresolved orthostatic hypotension (systolic blood pressure <90 mmHg; diastolic blood pressure <60 mmHg) as judged to be contraindicated by the Site Physician;
13. Current pressure ulcer of the arms, trunk, pelvic area, or lower extremities;
14. Psychopathology documentation in the medical record or history that may conflict with study objectives; and/or
15. Pregnancy or women who plan to become pregnant during the study period.

Definition of a traumatic or high impact fracture: Fracture from a forceful event, such as seen in any or all of the following, but not limited to these circumstances:

- ✓ Fracture from a motor vehicle accident
- ✓ Fracture from a fall from a height greater than adult height standing (i.e. down steps or stairs)
- ✓ Fracture from a heavy object falling on any lower extremity body part

Definition of a fragility, minimal trauma, or low impact fracture in the nonSCI population by the National Osteoporosis Foundation (NOF) is: Any fall from a standing height or less, that results in a fracture." Normal bones should be able to sustain a fall from this height, without a fracture, unless there is some underlying cause to suspect a bone disorder, such as osteoporosis or osteopenia that weakens bone structure. In SCI, a fragility fracture may include any, or all, but are not limited to the following conditions and/or circumstances:

- ✓ Fracture that occurred without the person having knowledge of the occurrence or cause
- ✓ Fracture that resulted from a fall from a wheelchair, bed, toilet, etc.
- ✓ Fracture that occurred while performing stretching
- ✓ Fracture that resulted from, or during, a transfer
- ✓ Fracture from bumping or banging the lower extremity
- ✓ Fracture from dropping the foot to the ground or wheelchair footplate
- ✓ Fracture from a light object falling on any lower extremity body part
- ✓ Fracture from carrying something or someone in their lap

And thirdly, potential participants who pass the Screening criteria (i.e., the inclusion and exclusion criteria) will be eligible for the five-session EAW basic training. The final part in the eligibility

process is the EAW Basic Skills Test after the five-session initial training; those potential participants who pass the EAW Basic Skills Test are then eligible to be randomized.

C. Exoskeleton Component ("Training the Trainers")

Basic and Advanced ReWalk training courses will be provided by ReWalk Robotics, Inc. at each of the sites for the designated ReWalk Trainers. Trainers will be taught how the device functions to accomplish walking. Initially, the Trainers will practice using the exoskeleton with another able-bodied member of the research team. The Trainers will practice fitting the device and teaching the able-bodied person how the device functions and how to use the computer settings. Once the Trainers are competent with the device in an able-bodied person, they will be able to fit the device and train the enrolled Veterans with SCI in the exoskeleton. Trainers will demonstrate an understanding of the system components and be able to teach the participants and their walking companions the system components. Trainers will demonstrate understanding of how the communicator functions and what the icons represent. Trainers will know where the different wire connections should be placed and understand the different error codes if a connection were to become loose, or if the battery power is low. They will be required to identify any defect or damage to any system components to ensure continued safe use. They will demonstrate knowledge of the strapping system and locations of required strapping. They will need to have working knowledge of all the screw locations to be able to make adjustments in the limb lengths. Trainers will need to have a working knowledge of the exoskeleton software and what each component does and how changing the values will change the walking parameters. The Trainers will need to be able to follow the standard operating procedures (SOP) manual in the event that there is a defective system.

D. ReWalk Fitting

The goals of the fitting are to properly measure the hip and lower limb segments of the participant and to adjust the exoskeleton to those measurements. The proper fit to the exoskeleton consists of: pelvic width, determined according to the width of the user's waist; thigh length, by measuring from the most prominent point of the greater trochanter to the joint line of the knee; and shank length, determined by measuring the knee joint line center to the bottom of the foot. Each Trainer will be required to observe areas of contact points with the device as these may be prone to skin breakdown. Additional padding, as applicable will be added in appropriate locations in order to avoid any skin breakdown. If skin breakdown occurs, Trainers and/or the Site Physician will need

to determine if training should be discontinued, or if adjustments to the padding can be put in place to avoid farther breakdown and allow the wound to heal with continued training. Skin breakdowns are to be reported as adverse events (AEs), unless otherwise indicated as an SAE according to the SAE criteria.

E. EAW Basic Skills Test

Initially, the participants will be required to learn to transfer in and out of the device, and how to don and doff the device. They will be provided an explanation of how the system works and what to expect when using the exoskeleton for ambulation. Videos may be used to assist with the verbal explanations. Each participant will be required to demonstrate the ability to perform a set of basic skills at the level of 4 (Minimal Assist) according to the following Functional Independence Measurement (FIM) scale rating:

<u>FIM Scale for level of assistance (LOA) during EAW</u>

- 7 Complete Independence (CI) (timely, safely, no assistive device used) (Note, while CI is a part of the FIM scale, it is not applicable for this study as all participants will require the use of the exoskeleton, thus negating the ability of complete independence.);
- 6 Modified Independence (MI) (extra time, patient requires an assistive device, no assistance);
- 5 Supervision (S) (cuing, coaxing, prompting);
- 4 Minimal Assist (Min) (performs 75% or more of task);
- 3 Moderate Assist (Mod) (performs 50%-74% of task);
- 2 Maximal Assist (Max) (performs 25% to 49% of task);
- 1 Total Assist (TA) (performs less than 25% of task); or
- 0 Activity does not occur.

The EAW Basic Skills (with the Trainer using the ReWalk mode selector wrist band), assessed after five sessions, are as follows:

1. Standing up;
2. Sitting down;
3. Two-handed balance for 30 seconds;
4. One-handed balance with the left arm, maintaining balance for 30 seconds;
5. One-handed balance with the right arm, maintaining balance for 30 seconds;

6. Perform a weight shift where crutch support is needed behind the participant, followed by a weight shift where crutch support is needed to either side, followed by a weight shift where crutch support is needed in front of the participant;
7. Shift their center of mass so that their weight is on the right leg;
8. Shift their center of mass so that their weight is on the left leg;
9. Able to adjust their weight so that it is evenly distributed between both feet; and
10. Able to take two consecutive steps on each leg.

These skills are needed for progression to walking and are expected to be achieved by session five or the participant will not be permitted to continue with further training. In our experience, most participants learn these skills in one or two sessions, but those who took longer than five sessions were likely not to achieve the needed skill set for the recommendation for home/community use of the exoskeleton by 20 to 30 sessions. Once the participant is able to perform the one-handed static balance for a minimum of 30 seconds, the standing up and sitting down tasks with a FIM score of 5 (supervision), they will be taught to use the mode selector wrist band and asked to wear it throughout the remaining training sessions. Participants who are screening failures due to an inability to pass the five-session Basic Skills Test may be re-screened at a future time, provided no new potentially eligible candidates are available. However, once a participant is randomized, they may not be re-randomized at a later date.

F. EAW Walking Skill Progression

Each training session will have predetermined goals that will be communicated to the participant. Initial goals will be to coordinate crutch timing with the end of swing phase, followed by practicing appropriate weight shifting for walking. Once these basic concepts are learned and the participant is stable when balancing with one crutch, then the use of the mode selector will be incorporated. As a participant progresses, system parameters should be adjusted according to the SOP manual for walking progression in order to obtain faster walking speeds.

The initial computer settings will be the same for all participants as follows:

1. Normal walking mode;
2. Hip flexion of 18 degrees;
3. Knee flexion of 39 degrees;
4. Step time of 1200 ms;
5. Delay between steps of 300 ms;
6. Tilt angle of 9 degrees;

7. Walking resistance of 7; and
8. Standing resistance of 10.

Settings are expected to change as the participant becomes more comfortable with using the exoskeleton. Hip flexion should be changed by 1 or 2 degrees in order to increase the step length as a participant can perform longer weight shifts. Knee flexion should only be adjusted if the foot catches during terminal stance and the initiation of the swing phase of the gait. The step time should be decreased by 100-200 ms increments as the participant becomes more comfortable with the frequency of weight shifting. Delay between steps should be lowered by 50-100 ms as participants become better with weight shifting from one foot to the other during EAW. The tilt angle should remain within a range of 7-10 degrees but could be decreased or increased slightly depending on the participant's stability during standing. Walking resistance should be increased by 1 if the system stops too easily, or the participant has more tone in the legs. Walking resistance should be decreased when walking on smoother surfaces such as tile, or if the participant has difficulty stopping the unit on command or when desired. Standing resistance should remain the same.

G. EAW Companion Training

The FDA has designated this class of device to be used in conjunction with a trained walking companion. The study calls for the use of a "walking companion", not a caregiver *per se*. The companion can be anyone who has met the companion criteria (below) and has undergone formal training with the participant on the use of the exoskeleton. Companions may be a family member, friend, neighbor, volunteer, or other such individual. Therefore, the companions are not likely to be the typical caregiver. That being appreciated, if a "caregiver" is available, eligible and agreeable, they may be an EAW companion.

Each participant will be required to have an EAW companion that will accompany them when the participant is using the device. There may be multiple people trained to be the EAW companion. An EAW companion will always need to be in close proximity to the participant when the system is in use while in the home or community environments. The companion's job is to be there to assist the participant with tasks such as navigating doors, bring them a chair, picking up items needed from the floor, countertops, furniture, tabletops, etc., provide close guard or contact assist when necessary, moving the device for donning or after donning, and to assist the participant with device checking, battery charging and storage. If a participant does not have a suitable companion, then they will not be eligible to participate in the study and are considered to be a screening failure.

Training of more than one companion is strongly encouraged. The potential participant may have up to three companions who share in the EAW training with the participant. All companions will be provided a separate consenting process and will either sign an informed consent form and HIPAA authorization or assent over the phone to be enrolled. If a companion withdraws during the Intervention Phase and no other companion was trained or available, the participant must complete additional companion(s) training. The new companion must pass the EAW Advanced Skills Test with the participant to be given clearance to return to the home/community use environment with the exoskeleton.

The companion screening criteria are meant to be general guidelines, but it is ultimately in the clinical judgment of the Site Physician to permit an acceptable companion.

Companion Criteria:

1. Male or female, age $\geq$18 years;
2. Demonstrates understanding of the time commitment to be a companion (to be available for approximately one-third of the training sessions, and walking at home or in the community with the participant);
3. The companion and the user are willing to receive training on how to assist the user with donning/doffing the device, use of the controller, and performing the standing, sitting, walking, and stopping skills while in the device;
4. The companion and the user are able to understand how to operate, charge, and maintain the device;
5. Demonstrate proper care and storage of device; knowledge of the major components of device (battery, waistpack, computer, right and left leg connection checks in computer waistpack);
6. Demonstrate understanding that the companion plus the user are examined as one team for the exoskeleton skills assessments;
7. The companion provides close supervision to minimal physical assistance to the participant when s/he is in the exoskeleton and provides assistance with tasks that may be difficult for the participant to perform while in the exoskeleton (i.e. opening manual doors and holding elevator doors);
8. The companion remains alert and on the lookout for potentially dangerous obstacles. If the companion sees a situation which could be dangerous, s/he should feel comfortable expressing their concerns to the user in the exoskeleton;

9. The companion ensures that the exoskeleton is used with the crutches at all times and in the manner in which both were taught;
10. In the event of equipment malfunction, the companion will be able to assist the participant down to a flat surface and be able to direct an additional individual in assisting them, if necessary, for a low impact descent to the floor;
11. In case of a medical emergency, the companion will be able to get help for the participant including activating an emergency response and be able to direct an additional individual in assisting them, if necessary; and
12. The Site Physician and study team members must deem the companion physically able to assist the participant with tasks outlined in the exoskeleton skills inventory (i.e. the companion is able-bodied and is physically able to bend, stoop, squat, kneel, etc.).

During the weekly contact with the participant, the study staff will inquire about the frequency of walking and the availability of the companion. If the companion has not been able to walk with the participant in a past week, the study staff will try to resolve the problems. Additional companions will be suggested. Availability of the EAW companion(s) is an important consideration and a close eye on this process will be necessary. Since these exoskeletal devices are going to require walking companions as per the FDA, it will be important to know the feasibility of our Veterans and military members with SCI to have viable walking companions for use of an exoskeleton in the home/community environment. The number of eligible companions and the actual amount of walking time that the companions are available for the participants during the home-use phase can be determined in the Feasibility component of the study. If the companion requirement proves to be an obstacle during the feasibility, it can be addressed with each case and solutions can be made available for all study participants. If a companion is lost or withdraws from the study, then the participant will be obligated to re-train another companion, or the participant will be terminated from the study, an outcome that will be strenuously avoided. Of note, as a consequence of this clinical trial, it will also be determined if identifying a companion is a major obstacle to the use of the exoskeleton in our Veteran or military member population with SCI because, as previously stated, these devices cannot be prescribed for home use without a companion in attendance when this device is being used.

H. EAW Advanced Skills Test

The EAW Advanced Skills are in addition to the EAW Basic Skills. The EAW Advanced Skills Test will be passed by the participant and their companion(s) prior to being sent home with an

exoskeleton and as soon during the training course as the ReWalk Trainers and the participants (with their companions) feel they can achieve the skills (at a minimum of 20 sessions). However, if by 30 sessions the participants are unable to achieve these skills, they will not be sent home with the device and will be treated as a study “failure”. Participants (with their companions) will need to demonstrate the following skills according to the SOP manual:

- Transfer into and out of the exoskeleton;
- Don and doff the device with all the straps and any additional padding provided by the Trainer in the correct places;
- Standing balance using both crutches for a minimum of one minute, showing good vertical position;
- Standing balance with one arm for 30 seconds with the ability to reach for the remote wrist band communicator;
- Perform sit-to-stand and stand-to-sit;
- Demonstrate full understanding of the sit, stand, walk, and manual mode functions of the remote communicator;
- Perform a 10 meter walk test without any stops 5 times at a speed greater than 0.40 m/s (10m EAW in ≤25 seconds);
- EAW ≥110 meters in a 6-minute walk test;
- EAW 10m on asphalt in ≤25 seconds;
- EAW timed-up-and-go (TUG) in ≤90 seconds;
- EAW ≥20m over surfaces such as tile, carpet, grass, and concrete in ≤60 seconds;
- EAW up and down a ramp that meets or exceeds Americans with Disabilities Act (ADA) specifications;
- EAW while talking and answering questions;
- EAW in a noisy environment;
- Reach for and remove an object from an overhead cabinet to a countertop level;
- Turn 180 degree while EAW;
- EAW through a door threshold;
- EAW to a wall, turn and lean (“wall rest”);
- Explain purpose of the “graceful collapse” function in case it of emergency;
- Demonstrate with companion the graceful collapse function;
- Demonstrate ability to check skin integrity;
- Demonstrate knowledge equipment components; and
- Able to charge the system at the end of the day

I. Weekly EAW Activity Log

ReWalk Trainers will be required to follow-up with each participant on a weekly basis. There will be a standard list of questions as per the SOP manual to ask in order to ensure the system is fully functional. If there have been any adverse events, or if the participant has any questions, s/he will be informed to contact a member of the site's study team. The participant will be required to complete a weekly EAW Activity Log of the location of where the device was used and for how long.

Intervention Group: Training for the Intervention group will consist of 25±5 sessions of EAW training followed by an EAW Advanced Skills Test that will determine the eligibility of the participant to take the device home. Training will take place at the VA hospitals, in the CBOCs under the same FWA as the sites, or in the participant's home/community. Participants in the Intervention arm will take the device home to use. During the training and home/community use components of the study, the built-in step counter on the device will record the weekly total number of steps taken. The EAW Activity Log will be completed by each participant on a weekly basis to record the location, duration and frequency of device use. The Trainers will follow up weekly with the participants for completion of this task.

Control Group: In order to provide a similar level of attention to the Control group during the Orientation/Training phase, the Control group will receive interviews to identify types of physical activities in which they usually participate. Control participants will be instructed to complete the Usual Activity Log on a weekly basis during the Orientation/Training course and during the four-month Intervention phase. Weekly contact by the study team will be provided to the Control group during the Orientation and Intervention phases, in person, over the phone, or through secure messaging using My HealtheVet.

IX. OUTCOME MEASURMENTS

The frequency and specific time points of each of the outcome measurements can be found in the "Training and Testing Schedules" (Table 6).

The two primary, major secondary and secondary outcomes are measured at baseline, post Orientation/Training phase, post 2-month Intervention phase, and post 4-month Intervention phase. The main comparison for the two primary and major secondary outcomes is the change in

the outcome measures from baseline to post 4-month Intervention phase.

A. Primary Outcomes

1. Mental Health Component Summary (MCS) of the Veterans Rand-36 (VR-36) for Vitality, Social Functioning, Role-Emotional, and Mental Health.
2. The sum T-score for the SCI-QOL Physical Medical Health domain (three item banks of Bladder Management Difficulties, Bowel Management Difficulties, and Pain Interference).

B. Major Secondary Outcome

1. Total body fat mass (by DXA scan) (results will be blinded)

C. Secondary Outcomes

1. Global Impression of Change Scale (Participant- and Companion-rated);
2. PROMIS Sleep Disturbance T score;
3. Assessment of bowel function for:
    a. Bowel evacuation (BE) time per episode in the past week,
    b. Frequency of BE episodes,
    c. Number of self-reported “natural” bowel movements in the past week,
    d. Frequency of digit stimulation in the past week,
    e. Stool consistency by the Bristol Stool Scale in the past week, and
    f. Frequency of enema use and amount of laxatives and/or stool softener used in the past month, and
    g. Frequency of bowel incontinence/accidents in the past month;
4. SCI-QOL Physical-Medical Health for bladder complications (scale) and pain behavior (scale);
5. SCI-QOL Emotional Health for positive affect and well-being, depression, anxiety, stigma, resilience, grief-loss, self-evaluation, and trauma;
6. SCI-QOL Social Participation for ability to participate in social roles and activities, satisfaction with social roles and activities, and independence;
7. SCI Functional Index (SCI-FI) for Physical Function for basic mobility, ambulation, fine motor, self-care, wheelchair mobility, and assistive technologies;
8. Abdominal fat mass by DXA scan (this is acquired as part of the total body scan);

9. Lipid profile analysis for HDL-c, LDL-C, TG, and TC will be shipped to the Chair's Office for batch analysis by the JJPVAMC laboratory (results will be blinded); and
10. FPG and FPI for HOMA-IR calculation. FPG and FPI will be shipped to the Chair's Office for batch analysis by their Core research laboratory.

D. Additional Assessments

1. EAW Basic Skills Test will be performed:
    a. During the five-session basic EAW Training (both groups),
    b. Post Training phase (Intervention group only), and
    c. Post 4-month Intervention phase (Intervention group only);
2. EAW Advanced Skills Test performed:
    a. Post Training phase (Intervention group only) and
    b. Post 4-month Intervention Phase (Intervention group only).

E. Quality Control (non-outcome) Measurements

1. Weekly SOC activities (Usual Activity Log) will be recorded for wheelchair activities and other, non-wheelchair activities (e.g., standing frame, swimming, or other non-wheelchair activities). This is measured weekly in both groups.
2. In the Intervention group only;
    a. Weekly EAW Activity Log in the Intervention group only,
    b. Weekly number of steps taken recorded on the step counter, and
    c. Walk tests and mobility assessments are recorded during each session once the participant is able to perform them and for the duration of the Training phase for the:
        i. 10 meter walk test for time (10mWT),
        ii. 6 minute walk test for distance (6minWT), and
        iii. Timed-up-and go (TUG).
    d. Walk tests and mobility assessments are recorded at months 2 and 4 during the Intervention phase as an outpatient visit.
3. Exit survey: participants and companions will be asked questions about their satisfaction with the study overall, and specifically about the device and training.
    a. Previously randomized participants and their companions will be called to complete the "Exit Survey".

F. Methods for Obtaining Outcome Measures Data

The Site ReWalk Trainers and Assistant ReWalk Trainers will perform the mobility skills and walking tests and be responsible for recording the EAW data. Exoskeletal-assisted mobility walking test assessments are measured in distance and/or time. Other mobility skills such as arresting gait on command, maneuvering to a wall rest, navigating doors, etc., will be scored one session per week as “able” or “unable” to perform the task; in addition, the level of assistance needed to complete the task will be recorded with each assessment.

The Site Coordinator will be responsible for administering the QOL outcome assessments (VR-36, SCI-QOL, Global Impression of Change Scale (Participant- and Companion-rated), PROMIS Sleep Disturbance, SCI-FI, and bowel function surveys) through in-person or phone interviews. The Site Coordinators will record all answers on the fixed forms. The site Trainers may assist as back-up QOL assessors only if necessary. In order to have continuity of assessment, the Site Coordinators will administer the surveys at all of the time points. Each SCI-QOL, PROMIS Sleep Disturbance and SCI-FI survey is reported as a T-score (mean=50, SD=10). The VR-36 is in a fixed form and administered sequentially.

The Site Coordinator will be responsible for administering the Global Impression of Change Scale. This scale is a self-assessment completed by the participant (and separately by the companion) with questions about feeling better from “No change (or condition has become worse); Almost the same (hardly any change at all); A little better (slightly noticeable, but has not made any real difference); Somewhat better (the improvement is small, slightly noticeable difference); Moderately better (the improvement is a noticeable change); Significantly better (a definite improvement, a real and worthwhile difference) to Extremely better (a comprehensive improvement that has made all the difference)”. Each question is scored from 1 to 7, with 1 being no change or worse and 7 being the best change in improvement. The change reported on participant’s self-assessment on the global scale will be examined with the change reported on the SCI-QOL for correlation. The companion’s impression of the participant’s global change will be used as a proxy validation of the participant’s self-assessment. Analysis of the mean change scores for both tools would help to confirm the clinical relevance of the hypothesized 10% improvement from baseline in the SCI-QOL as a clinically relevant improvement.

The Site Coordinator will be responsible for administering the bowel function surveys and will explain to each participant to select the response that best represents what is asked by the

question. These set of questions ask about the participants views about their bowel function. The Site Coordinator will ensure that each participant answers every question, by marking the answer as indicated. If the participant is unsure about how to answer a question they will be instructed to provide the best answer they can. The Site Investigator will be responsible for the history and physical examination and administering the International Standards for Neurological Classification of SCI (ISNCSCI) Examinations for level and completeness of SCI. A radiology technician or designated DXA technician at each site will perform the DXA scans. The cost of this service has been added to the budget.

G. Schedule of Observations and Laboratory Tests

A detailed description of the schedule of tests is provided (Table 6). Five DXA Scans will be performed over the course of the study, one for screening BMD and four for total body fat mass (includes abdominal fat mass) measurements. Four fasting blood draws (15 ml each time) will be performed over the course of the study for the lipid profile and FPG and FPI. Bilateral calcaneus x-rays will be obtained during screening. Other data collection will be in the form of Case report forms (CRFs), sequential fixed forms, and Microsoft Access database or potentially other electronic databases behind the VA firewall. Data collection may also take place through secure messaging using My HealtheVet.

X. PERFORMANCE MEASURES AND STUDY MANAGEMENT

The primary performance measure for the sites is enrollment. Performance measures will be established early in the start-up year by review of the prescreening and screening forms. Sites that do not meet the goals of 100 participants prescreened and 60 participants screened will be notified for consideration of probation. Across all study sites, 160 participants are to be randomized. Each site is expected to randomize between 4 and 24 participants.

The overall management plan is for the sites' study team (Site Investigator, Site ReWalk Trainer, Site Assistant ReWalk Trainers, and Site Coordinator) at the participating medical centers to conduct the daily activities of the study. The Chair's Office and the CSPCC at the Perry Point, MD VAMC will provide leadership and guidance to the local sites as specifically described below.

A. CSPCC

The Perry Point Cooperative Studies Program Coordinating Center (CSPCC), located in Perry Point, Maryland, will provide administrative, data processing and statistical support for the study. Data forms will be submitted by the local site study team members to the CSPCC for processing. The CSPCC will edit the data and create the study database. CSPCC staff will provide guidance on completion of forms. All reports during the ongoing phases of the study and the final statistical analyses will be the responsibility of the CSPCC. CSPCC staff will also monitor study progress to ensure that the study is proceeding as scheduled. A CSPCC study team dedicated to this study will be established. This team will be headed by the study biostatistician and will include a CSPCC project manager, a statistical programmer, a database programmer and two computer assistants.

B. Office of the Chair

Ann M. Spungen, EdD, study Chair and William A. Bauman, MD, Co-Chair will provide leadership for the study. The Chair's Office is located at the James J. Peters VAMC, Bronx, New York. In order to facilitate organizing and coordinating this ten-site multi-center study, the Chairs have elected not to include the Bronx VA as a study site. The Chair's Office personnel will be in routine contact with the participating sites to ensure that the study is performed in accordance with the protocol and that each local site team is able to meet the enrollment goals and follow-up activities on schedule. The Chair and Co-Chair will preside over all meetings and will represent the study, along with the study biostatistician at any meetings of outside review committees. The Chair's Office will be funded with a full-time National Study Coordinator (1.0 FTE), a Co-National Coordinator (1.0 FTE), a Staff Assistant (1.0 FTE) and a part-time iDXA Analysis Technician. Dr. Spungen will be responsible for all study executive decisions. She will serve as the Chair of the CS #2003 Executive Committee. Dr. Bauman, Co-Chair, will provide medical advice and direction for the National Coordinators and administrative staff as needed. Dr. Spungen will be a resource for protocol details and compliance issues. She will serve as a liaison with other study personnel for the Chair's Office. Dr. Spungen is a member of both the Executive and Planning Committees.

Overall, the National Study Coordinators are responsible for maintaining enthusiasm, quality standards across the sites, discussing problems, problem solving, and identifying any procedural/definitional modifications that might be required. The CS #2003 National Study Coordinators will be responsible for overseeing all study activities in the Chair's Office on a day-

to-day operational basis. In addition to the specific job responsibilities outlined in the "Description of Responsibilities" section of this submission, the National Coordinator and Co-National Coordinator will:

1) Assist the Chairpersons in coordinating and administering all aspects of the study;
2) Assist the Chairpersons in monitoring the progress of the study;
3) Maintain close contact with the participating Site Investigators, Site ReWalk Trainers, and Site Coordinators to assist them in any procedural details of the study;
4) Maintain close contact with the study's supervisory committees; and
5) Work collaboratively with the Perry Point CSP Coordinating Center team to organize and plan periodic meetings of participating Site Investigators and other study team members for the purposes of reporting progress of the study.

XI. SAFETY MONITORING

Timely and complete reporting of safety information assists study management in identifying any untoward medical occurrence, thereby allowing: a) protection of safety of study participants, b) a greater understanding of the overall safety profile of the study treatments and therapeutic modalities, c) improvements in study design or procedures, and d) compliance with regulatory requirements. The Site Investigator will be responsible for reviewing the accuracy and completeness of all reported events, compliance with VA CIRB policies for reporting adverse events (AE), serious adverse events (SAE), Unanticipated Adverse Device Effects (UADE), and closely monitoring research participants at each study visit for any new SAEs.

A number of groups will be charged with monitoring the study. These groups include the Study Group, the Executive Committee, the Data Monitoring Committee (DMC), , the VA Central Institutional Review Board (CIRB), the CSPCC Human Rights Committee and the Cooperative Studies Scientific Evaluation Committee (CSSEC). In addition, the CSP Site Monitoring Auditing and Resource Team (SMART) will routinely visit the participating centers. This monitoring will not preclude the yearly monitoring that the local R&D Committee and CIRB must also perform.

A. The **Study Group** consists of all participating Site Investigators, Site ReWalk Trainers and Site Coordinators, as well as staff from the Chair's Office, CSPCC, and CSPCRPCC. It meets prior to the start of participant intake and annually to discuss the plans/progress of the study, as

well as to identify any problems encountered during the conduct of the trial. No outcome data are presented to this group.

B. The **Executive Committee** is the management and decision-making body for the operational aspects of the study. Chaired by Dr. Spungen, the committee consists of the Chair and Co-Chair, the study biostatistician, the Adverse Event Specialist, a minimum of three Site Investigators, and outside consultants, if necessary. This committee monitors the performance of participating medical centers and quality of data collected. The Executive Committee formulates plans for publications and oversees the publication and presentation of all data from the study. Permission from this committee must be granted before any study data may be used for presentation or publication. This committee typically meets on the same schedule as the Study Group. This group also does not receive outcome data during the course of the study.

C. The **Data Monitoring Committee** (DMC) is a group of outside experts in the area of spinal cord injury, clinical trials and biostatistics that reviews the progress of the study and monitors participant enrollment, outcomes, adverse events, and other issues related to patient safety. The DMC makes recommendations to the Director of the Cooperative Studies Program (CSP) as to whether the study should continue or be modified or stopped. The DMC can consider patient safety or other circumstances as grounds for early termination, including either compelling internal or external evidence of treatment differences or lack of feasibility of addressing the study hypotheses (e.g., poor patient intake, poor adherence to the protocol). The DMC will meet annually to review data reports prepared at the Perry Point CSPCC. At the six-month interval between the annual meetings, the DMC will receive another data report for review without a meeting. Any member of the DMC can ask for a meeting of the group if s/he feels that it is necessary, based upon the data.

D. The **VA Central IRB** (CIRB) will be the study's primary IRB and the IRB of record for the study. It will be responsible for the initial and continuing IRB reviews of the study. The CIRB must review and approve amendments (changes to inclusion/exclusion criteria, protocols, informed consents, etc), deviations, and review reports about adverse events and problems, complaints, terminations, etc.. The CIRB approves the original informed consent template and any requested changes to the informed consent forms. The CSPCC Human Rights Committee (HRC) may be asked to convene if there is any serious adverse event requiring its attention.

E. Definitions of Adverse Event (AE), Serious Adverse Event (SAE), and Unanticipated Adverse Device Effect (UADE)

An Adverse Event (AE) is defined by the International Conference on Harmonization (ICH) for Clinical Safety Data Management (ICH-E2A) as “any untoward medical occurrence in a clinical investigation participant that is subjected to one of the study treatments that does not necessarily have to have a causal relationship with the treatments. An AE, therefore, can be any unfavorable or unintended sign (including an abnormal laboratory finding), symptom, or disease temporally associated with the study interventions.” In this study, all AEs will be collected by the Sponsor. The reporting period for AEs begins when the participant signs the informed consent form and continues until the participant’s completion or early termination of participation or the end of the study.

A Serious Adverse Event (SAE) is defined by the ICH for Clinical Safety Data Management and CSP Global SOP 3.6.2, as any untoward medical occurrence that: results in death, is life threatening, requires inpatient hospitalization or prolongation of existing hospitalization, results in persistent or significant disability or incapacity, a congenital anomaly/birth defect, or any other condition that, based upon medical judgment, may jeopardize the subject and require medical, surgical, behavioral, social or other intervention to prevent such an outcome. Participants will be monitored for SAEs at each study visit. All SAEs will be reported on an SAE Form, regardless of relationship to the intervention. Active monitoring for SAEs begins at the time the Informed Consent Form is signed and continues until the earlier of the 30 days after the participant’s completion or early termination of study participation or the end of the study. All SAEs require prompt reporting to the CSP Clinical Research Pharmacy Coordinating Center (CSPCRPCC) within 72 hours of the Site Investigator becoming aware of the event. All **unresolved** SAEs must be followed up at least every 30 days until resolved or when no further change is expected (i.e., event is ongoing recovering/resolving or not recovered/not resolved. No changes to the event will occur in the future.) All SAEs are followed until no changes are expected or the study is discontinued.

An unanticipated adverse device effect (UADE) is defined in 21 CFR 812.3(s) as any serious adverse effect on health or safety or any life-threatening problem or death caused by, or associated with, a device, if that effect, problem, or death was not previously identified in nature, severity, or degree of incidence in the investigational plan or application (including a supplementary plan or

application), or any other unanticipated serious problem associated with a device that relates to the rights, safety, or welfare of subjects.

Starting with each site, at the local level, the site study team members will be responsible for recording adverse and serious adverse events, as well as unanticipated adverse device effects and submitting them to the appropriate authorities in the time frames listed below.

F. Serious Adverse Event (SAE) Reporting

Any Serious Adverse Event (SAE) is reported on both the SAE and AE case report forms. The Site Investigator will be responsible for reporting a SAE, ascertaining the participant's study identification number, date of event, and will determine if the SAE is related, possibly related or not related to the protocol. A description of any outcome or response to the SAE will also be included in this report. As more information about a SAE becomes available, updates to this initial report must be made on the SAE Follow-Up form. It is the responsibility of each Site Investigator to report SAEs from their site to the CIRB within the timelines they require. SAE data are tabulated by the CSPCC at Perry Point, MD and the CSPCRPCC at Albuquerque, NM, and summarized and in the report to the DMC. ReWalk Robotics, Inc. will be provided a summary of SAEs annually.

G. Adverse Event (AE) and Unanticipated Adverse Device Effect (UADE) Reporting

Any mild (awareness of symptom, but easily tolerated), moderate (discomfort enough to cause interference with usual activity) or severe (incapacitating with inability to work or do usual activity) AE must be reported on the AE case report form. Any AE which also meets the criteria for a SAE must also be reported on the SAE form. It is the responsibility of each Site Investigator to report AEs from their site annually to the CIRB. Participants will be monitored at a minimum of one time per week for AEs and recorded on the appropriate form should any occur. AE data are tabulated by the CSPCC at Perry Point, MD and the CSPCRPCC at Albuquerque, NM and summarized in the report to the DMC.

UADEs must be reported by the clinical investigator to the sponsor and the CIRB, Investigators are required to submit a report of a UADE to the sponsor and the reviewing IRB as soon as possible, but in no event later than 10 working days to the sponsor and no later than 5 days to the CIRB after the investigator first learns of the event. This reporting will be to the CSP Clinical Research Pharmacy

Coordinating Center on an AE or SAE form, as appropriate, and to the CIRB on the relevant IRB form. The reporting period for UADEs begins when the participant signs the informed consent form and continues until the participant's completion or early termination of participation or the end of the study

As part of the orientation and training for each participant on the device, the expected occurrence of routine AEs will be described to the participants, including that they may experience skin abrasions, and hand, shoulder or any other joint discomfort. During the five-session Basic Training and in the Screening Phase and the Orientation/Training Phase, participants will be asked during each session if they are experiencing any discomfort or pain. Hand or shoulder discomfort, if they occur, are expected to occur early on in training when the participant is learning to use the device. Participants who report any pain or other medical problem will be referred to the Site physician. The Site physician will be responsible for taking the appropriate course of action for the AE or SAE. The study staff will be responsible for recording the occurrence of AEs or SAEs appropriately, as they occur. Additionally, the SMART unit will be charged with monitoring the informed consent documentations, source documents, AE and SAE forms, as well as assessing if the Site study team is carrying out the study correctly.

The Site Investigator will be responsible for monitoring these events on a regular basis at his/her site. The immediate and ongoing safety review of study data to monitor for evidence of adverse events will be conducted by the Cooperative Studies Program Clinical Research Pharmacy Coordinating Center (CSPCRPCC) in Albuquerque, NM. The Data Monitoring Committee (DMC) will review AEs and SAEs on a regular basis.

H. Participant Safety Stopping Rules

The Site Physician will make the decision for participants who have a safety or medical concern such as: skin breakdown, edema, joint discomfort, muscle soreness, extreme fatigue, or any other medical condition as to whether they are to be continued as is, continued with modifications to the equipment, temporarily discontinued, or withdrawn from the study. Re-entry to the Training or Intervention phases of the study will be based on the Site Physician's medical clearance and the participant's safety to begin again.

I. Site Participation Stopping Rules

Each site must enroll at least 8 participants in the first year of enrollment. Sites that do not meet this enrollment goal will be considered for probation. Rapid improvement is expected to avoid termination from the study. Sites that continue to fail to meet their recruitment goals will be discontinued and replaced with another site, if time permits during the study. The Chair's Office and the CSPCC will work with any site that is below recruitment goals to assist in overcoming obstacles which may have contributed to their falling behind, thus allowing them the opportunity to catch up. If this is not feasible, then resources will be shifted to either allow the other sites to recruit more participants, or phase out one site and start another.

Pre-screening and screening logs will be closely monitored by CSPCC and the Chair's Office. Study sites that have not met their recruitment goals will be closely reviewed for reasons why. An indicator for unmet enrollment, which can be assessed early in the start of the study, is lack of prescreened and screened potential candidates. Sites will be expected to prescreen 100 and screen 60 potential participants over the years of study enrollment. Viewing the sites' prescreening and screening histories early can help to identify sites that are not performing as expected. The feasibility component will be used to formally assess the enrollment rates for the first six sites.

J. Efficacy or Futility Criteria for Study Termination

The DMC will be responsible for review of the SAEs and AEs for each site and across the study as a whole. The DMC will make all decisions for stopping the study based on safety concerns, should any present. Early analyses before the study is completed may result in stopping of the study. For example, if it is statistically demonstrated that the results for either efficacy or futility to date could not be changed with further enrollment, the study may be stopped. When repeated significance tests are performed on accumulating data as part of a routine monitoring function, the overall type-I error rate is inflated and the probability of a false positive finding is also increased. A number of methods have been developed to provide guidance on study termination rules based on multiple looks at the primary outcome measures for the review committees while keeping the overall type-I error rate maintained at 5%. For this study, the O'Brien-Fleming method for alpha spending and rejection/acceptance boundaries will be suggested for Type-I error rate control (see Statistical Analysis Plan (SAP – Section XIII) for further details) but the DMC will make the final decision on the type of stopping rule that will be used for the study.

XII. QUALITY ASSURANCE PROCEDURES

Centralized and on-site reviews and monitoring of clinical site practices will be conducted at several levels. The National Coordinator will visit each site two times per year during the enrollment years to monitor the EAW Training, patient safety, and compliance with the study protocol. The Co-National Coordinator will visit each site one time annually during the enrollment years to monitor standardization of the QOL assessments and protocol compliance. Participant's medical records will be made available to the CSP Site visitors as a requirement for participation in this study.

This trial will be conducted in compliance with Good Clinical Practice (GCP) regulations. The intent of these regulations is to safeguard participants' welfare and assure the validity of data resulting from the clinical research. The VA Cooperative Studies Program will assist local site Investigators in complying with GCP requirements through its Site Monitoring, Auditing and Resource Team (SMART) based in Albuquerque, NM. SMART serves as the Quality Assurance arm of CSP for GCP compliance. SMART will provide training, manuals and materials to assist study personnel in organizing study files and will be available throughout the trial to advise and assist Site Investigators regarding GCP issues.

A. Summary of Monitoring and Auditing Plans

1. Monitoring Visits
   - One routine monitoring visit per site
   - Additional monitoring visits may be conducted as deemed necessary by study leadership or SMART.
2. Audits
   - Routine audits – independent site visits to one or more sites per year as determined by SMART.
   - For-Cause audits –independent audit of a site as requested by study leadership or CSP Central Office.
   - Audits may be scheduled or unannounced.

XIII. STATISTICAL ANALYSIS PLAN (SAP)

A. Introduction

There are two primary outcome measures in this study: the first is the score on the Mental Component Summary of the Veterans Rand-36 (MCS/VR-36) and the second is the sum T-score for the SCI-QOL Physical Medical Health domain (three item banks for bladder management difficulties, bowel management difficulties and pain interference). The primary study hypotheses are: 1) 33% of the intervention group (EAW+SOC) compared with 10% of the control group (SOC) will demonstrate a clinically relevant change (improvement) of ≥ 4.0 points in the MCS/VR-36, for greater vitality and social functioning, and improved role-emotional and mental health, from baseline to the end of the intervention phase, and 2) 42% of the intervention group compared with 10% of the control group will demonstrate a clinically significant improvement from baseline of ≥ 10% in the sum T score, indicating improvement in patient reported outcomes for SCI-QOL bladder management, bowel management and pain interference.

A major secondary objective of the study is to demonstrate that participants who use the exoskeleton in addition to SOC (EAW+SOC) will have at least a 1.0 kg loss in total body fat mass by the end of the four-month intervention compared to those who receive SOC only. Other secondary objectives are to demonstrate that participants in the EAW+SOC group will have greater net improvements than participants in the SOC only group in the following outcomes: 1) Global Impression of Change (participant- and companion-rated), 2) disturbed sleep as measured by the T-score of the Patient Reported Outcomes Measurement Information System (PROMIS) Sleep Disturbance, 3) self-reported methods and measures of bowel function, 4) sum T scores of the SCI Functional Index (SCI-FI) physical function short forms, 5) sum T scores of the SCI-QOL Emotional domain, 6) sum T scores of the SCI-QOL Social Participation domain, 7) lipid profile for high density lipoprotein cholesterol (HDL-c), low density lipoprotein cholesterol (LDL-c), triglycerides (TG), and total cholesterol (TC), and 8) fasting plasma glucose (FPG) and insulin (FPI) levels for calculation of Homeostasis Model of Assessment-Insulin Resistance (HOMA-IR).

B. Sample Size

Using preliminary data generated from the Exoskeletal-assisted Walking Program at the James J. Peters VA Medical Center, three separate power calculations were performed, one for each of the primary outcome measures, the MCS/VR-36 and the sum t-score of the SCI-QOL bladder, bowel and pain item banks, and one for the major secondary measure, total body fat mass. The sample

size estimates are based on the preliminary data from these three outcome measures as a proportion of participants who achieved a clinically significant change score (MCS/VR-36 and Total Body Fat Mass) or a clinically significant percent change (SCI-QOL PMH).

Sample size/power calculation for the Primary Outcome (1) MCS/VR-36: A minimally important difference for the MCS/VR-36 is 4.0. It is hypothesized that 33% of the Intervention group and 10% of the Control group will achieve a 4.0 or greater change on the MCS/VR-36 (Table 7).

Sample size/power calculation for the Primary Outcome (2) SCI-QOL for Bladder, Bowel and Pain: An improvement of 10% in the sum T-score of the patient-reported outcomes from the combined SCI-QOL item banks for bladder management difficulties, bowel management difficulties and pain interference is clinically significant. As such, it is hypothesized that 42% of the Intervention group compared with 10% of the Control group will demonstrate a clinically significant change of 10% improvement from baseline to the end of the intervention phase on the sum T-score in the combined SCI-QOL item banks for bladder management difficulties, bowel management difficulties and pain interference. (Table 7).

Sample size/power calculation for the Major Secondary Outcome for Total Body Fat Mass: Patients with a new SCI can be expected to gain an average of 6.0±8.9 kg of fat mass in the first 14 months after injury (manuscript in preparation from our Center). This gain in fat mass appears to continue throughout the duration of their life after SCI. As demonstrated in the preliminary data, an intervention that supports a 1.0 kg (2.2 lb.) loss of fat mass is a substantial amount of fat mass to lose in three months for a person with SCI. Very few interventions have demonstrated changes in body composition of this magnitude in the SCI population. It is expected that 35% of the Intervention group compared with 10% of the Control group will maintain total body fat mass loss during the home/community use phase of the intervention.

Assuming the proportion in the control group achieving a clinically meaningful change to be 10% (or 0.100) for all three outcome measures, and estimating the attrition rate at 15%, the sample sizes range from a low of 71 per group (95% power) for the SCI-QOL PMH outcome to a high of 79 per group (80% power) for the MCS/VR-36 group. As a note about Table 7, to adjust for multiple outcome measures, a significance level of 0.025 was used in the sample size calculations for the co-primaries (MCS/VR-36 and SCI-QOL) and also for the major secondary outcome measure (Total Body Fat Mass). Based on the sample size and power calculations in Table 7, the study will enroll a total sample size of 160 participants, 80 per group.

Table 7: Sample Size/Power Calculations			
Two group continuity corrected χ^2 test of equal proportions (odds ratio = 1) (equal n's)	**MCS/VR-36**	**SCI-QOL (PMH)**	**Total Body Fat Mass**
**Test significance level, α**	0.025	0.025	0.025
**1 or 2 sided test?**	2	2	2
**Control proportion, π_1**	0.100	0.100	0.100
**Intervention proportion, π_2**	0.333	0.420	0.350
**Odds ratio, $\psi = \pi_2 (1 - \pi_1) / [\pi_1 (1 - \pi_2)]$**	4.493	6.517	4.846
**Power (%)**	80	95	85
**n per group**	67	60	66
**n per group with 15% attrition**	79	71	78

C. Feasibility Phase

In order to confirm the hypothesized control proportion for the two primary and major secondary outcomes, to determine the feasibility of recruiting participants at each site, and to assess other operational aspects of the study, a feasibility phase has been built into this study. The planned duration of the feasibility phase of the study is one year. Six out of fifteen planned sites (including three sites that are designated CSP NODES) will be brought up in the first year.

During the feasibility phase, several site start-up activities and operational aspects of the study will be examined. These activities will include the hiring of site personnel (including classification of position descriptions, position announcements and recruitment), the processes for procuring and distributing the ReWalk and iDXA devices to the sites, the methods by which site staff are trained on the use of the devices and on data collection and management activities, the number of sessions needed for participants in the intervention group to pass the Advanced Skills Test, the practicality of Veterans or members of the military with SCI to find motivated and eligible walking companions, and attrition (drop-out) rates. The lessons learned during this phase will be implemented during activation of the remaining participating sites.

Recruitment rates at the six active sites will be closely monitored during this phase. The initial six sites are expected to enroll a total of 48 participants by the end of the first year. The actual recruitment experience of the six sites in the first year will be used to revise the projected recruitment rate for the remainder of the study and may lead to a lengthening or shortening of the total recruitment period, as appropriate.

Besides the verification of the assumed recruitment rate and other site start-up activities, the feasibility phase also provides an opportunity to assess the assumed success rates in the control (SOC) group. As stated above in section II, we have hypothesized that 10% (0.100) of the control

group will have a clinically meaningful change in the outcome measures for sample size/power calculations. Ten percent is thought to be a conservative estimate as there is no reason to expect the control group to have clinically meaningful changes in quality of life measures or in total body fat mass while continuing to receive SOC for their spinal cord injury. However, as a means of verifying the control proportion of 10% (or 0.100), the proportion of participants considered successful in the control group for all three outcomes and 95% confidence intervals for the proportions will be reviewed upon the completion of the feasibility phase. Based on the extensive experience with this population it is highly unlikely that the assumed success rates in the control group (SOC) will be any higher than the assumed 10%.

At the completion of the feasibility phase, the study will be moved to a continuation phase where the remaining sites (9 remaining sites) will be activated, and the remaining participants will be recruited.

D. Baseline Comparability

Baseline comparability between the treatment groups will be evaluated with respect to such variables as demographics (e.g., age, gender, and race) and baseline values of outcome measures (MCS/VR-36, SCI-QOL, total body fat mass, etc.). Chi-square and analysis of variance techniques, as appropriate, will be used to determine any differences in distribution of the variables across the treatment groups. Any variable that appears to be different between the groups ($p<0.10$) will be considered as a potential covariate in statistical analyses.

E. General Outcome Analysis Guidelines

All statistical tests will be 2-sided. The two primary and major secondary outcome measures will be tested at a 0.025 level of significance. Because of the large number of secondary outcomes to be analyzed, all other secondary outcomes will be tested at a significance level of 0.01 to maintain control over Type I error. SAS 9.4 will be used to conduct all the statistical analyses. A variety of analytic methods will be used for the primary endpoints, secondary endpoints and other analyses.

F. Analysis of Primary and Major Secondary Outcome Measures

The primary analysis will be based on an intent-to-treat (ITT) model. All participants who are randomized will be included for both groups, whether they are study completers (participants who complete the study through the four-month intervention phase) or early terminators (drop-outs). In order to test the hypotheses for the primary and major secondary outcome measures, each randomized participant will be deemed a success or failure at attaining: 1) $\geq$ 4.0 point improvement in the MCS of the VR-36, 2) $\geq$ 10% improvement in the SCI-QOL bladder management difficulties, bowel management difficulties and pain interference sum T score, and

3) ≥ 1 kg. total body fat mass loss. For each of the outcomes, the proportion of participants successful at achieving a clinically significant improvement will be compared between the two groups (Intervention vs. Control) using a chi-square analysis. These will constitute the primary and major secondary efficacy analyses. Participants who drop out will be treated as failures. As such, by design, there will be no missing data: participants either meet the outcome criteria (successes), do not meet these criteria (failures) or they drop out (also failures).

G. Secondary Analyses

In addition to the primary analysis, several secondary analyses will be performed and are described below.

1. Analyses will be performed that include only those participants who complete the study (excluding drop-outs):

    a) Chi-square analyses of the primary and major secondary outcome measures (proportion of successes) will be repeated for only those participants who complete the 4-month intervention phase.

    b) Analyses of the MCS/VR-36, SCI-QOL, total body fat mass loss, Global Impression of Change and other secondary outcome measures will include comparisons of the mean difference scores (change from baseline to the end of the intervention phase) of these outcome variables using t-tests.

2. Interim time points of data collection for all outcome variables have been incorporated into the study design. These time points are: baseline, after training/orientation phase, two months into the intervention phase, and then again after four months of the intervention phase (primary and major secondary outcomes main time point). Secondary analyses of the MCS/VR-36, SCI-QOL, total body fat mass loss, Global Impression of Change and other secondary outcome measures will include comparisons of the mean difference scores (change from baseline to the end of each time point) of these outcome variables using t-tests. Other secondary analyses of outcome measures will be performed that use all data available. For these continuous variables, repeated measures analysis of covariance will be used to analyze them, with baseline scores, plus any variables determined from the baseline comparisons, being used as covariates in the analyses. SAS MIXED and GENMOD procedures, which allow for the use of incomplete data sets, will be used if it can be safely assumed that missing data are missing at random. If this assumption is not valid, a multiple imputation strategy of the missing responses will be implemented which does not rely on the assumption that missing data occur at random. This will allow an intent-to-treat approach for the statistical analyses, where complete cases are not required.

3. An analysis to determine the characterization of the drop-outs will be performed by using descriptive and correlation statistics. The reasons for drop-out and the number of sessions/time points completed will be described. A correlation analysis will be performed with the reasons for drop-out and the demographic variables and other potential variables to identify characteristics of persons who drop out of the study.

H. Monitoring of Study by Study Group and Executive Committee

The Study Group (all of the Site Investigators) and Executive Committee will meet 6 to 9 months after patient recruitment begins and at annual intervals thereafter until the end of the study. Three weeks prior to these meetings and at 6-month intervals between the meetings, these groups will be provided a report that will allow them to assess study progress. Since both groups are composed of study participants, no outcome data will be provided in these reports. The information provided will include data on:

1. Screening, enrollment and retention
2. Patient background characteristics at entry
3. Data quality and protocol adherence.

1. Screening, Enrollment and Retention

During the pre-screening phase of the study, potential participants will be first contacted by the Site Investigator through chart reviews, other physician referrals, pre-existing knowledge about their patients, access to contact information in a VINCI database, physician referrals at other VA hospitals, and/or through study flyers and invitation letters. Study flyers may also be given to potential participants in the SCI Clinics, posted in local area hospitals, or distributed at local and national SCI events. Study flyers may also be posted generally in areas where potential participants may see the information (i.e. local chapters of Veterans Organizations, SCI sporting events) and included in newsletters or journals with a focus on the SCI population. Eligible and interested potential participants will be contacted by the Site ReWalk Trainers and/or Site Study Coordinators for initiation of the consenting process and the screening and baseline evaluations. Any participant who consents to the study and passes all screening and baseline evaluations is eligible to be randomized into the study.

The progress of patient accrual will be presented to the monitoring groups in two formats. Table 8 gives the first format, which presents, by site and month, the actual number of patients entered into the study. This table will indicate if recruitment is improving or worsening over time at the various sites.

Table 8. CS #2003 Number of Participants Entered Each Month by Site

Month	Site 1	Site2	…	Site 15	Total
September 2016					
October 2016					
November 2016					
September 2016					
.					
.					
.					
**Total**					

Intake data will also be plotted over time for the total number of participants recruited, as in Figure 9. An expected intake line is given for comparison purposes, assuming a staggered start of sites: 6 sites expected to enroll 48 participants in the first year of recruitment, 10 sites projected to enroll 80 participants during the second year of recruitment and 15 sites enrolling 32 participants during the third and fourth years of recruitment, for a total of 160 participants randomized.

Figure 9. CS #2003 Observed vs. Expected Recruitment

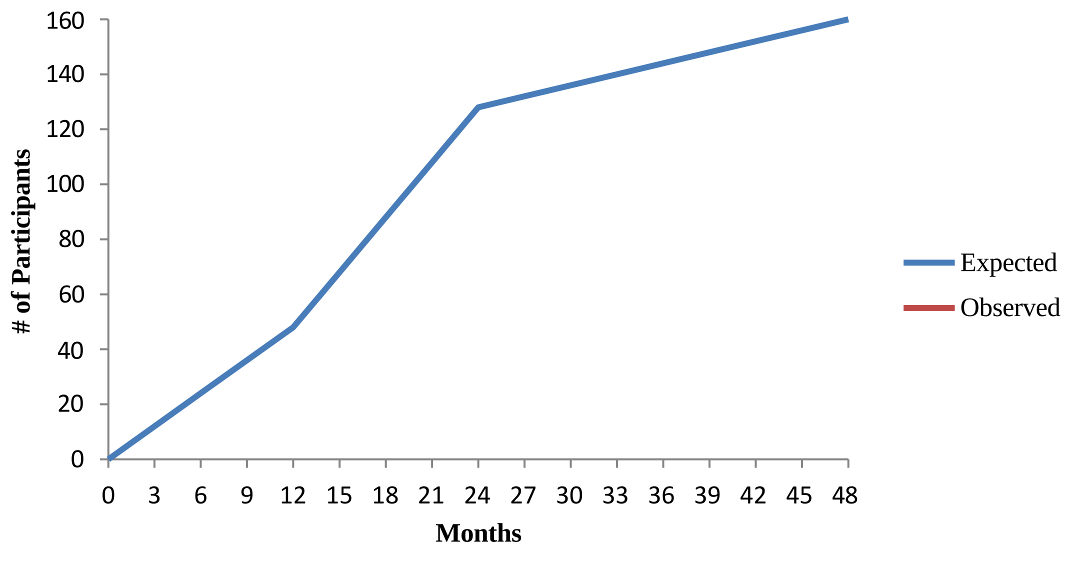

The number of participants screened and the number of those that randomize in the study will be presented in Table 9. The reasons for the exclusion of screened participants will be presented in Table 10.

Table 9. CS #2003 Cumulative Screening Summary: All Participants by Site

Site	Screened	Excluded	Randomized	% Excluded
1				
2				
3				
.				
.				
15				
**Total**				

Table 10. CS #2003 Summary of Ineligibility: Primary Reason for Exclusion, All Sites

Primary Reason for Exclusion	# Screened	# Excluded	% of Screened
Less than 18 years of age			
Duration of SCI less than 6 months			
.			
.			
.			
No companion to assist at home			
Inability to provide informed consent			
Diagnosis of neurological injury other than SCI			
Progressive condition likely to result in changing neurological status			
.			
.			
.			
Psychopathology documentation that may conflict with study objectives			
Pregnancy or women planning to become pregnant during study			
**Total**			

2. Background Characteristics at Entry

Background characteristics of study participants are collected on the Screening/Baseline and History & Physical Forms. Tables summarizing the important background characteristics by site will be prepared and submitted to the Study Group so they will have an idea of the population being studied and comparisons of enrollment among the sites can be made. This information will be presented as means and medians for continuous variables and as frequency tables for discrete variables. Table 11 shows how the continuous variable age will be presented. Other variables that will routinely be presented will include gender, race, education and smoking history. Analysis of variance and chi-square techniques will be used to identify any statistically significant differences that may exist among the sites.

Table 11. CS #2003 Age Statistics by Site

Site	N	Mean (Years)	Standard Deviation	Median
1				
2				
.				
.				
.				
15				
**Total**				

3. Data Quality and Protocol Adherence

The final type of information that will be given to the Site Investigators is data that will allow them to assess the quality of the data being submitted as well as how well the sites are adhering to the protocol. These data will be given by site, so sites performing substantially below average can be identified and remedial action taken to improve their performance.

One piece of information that will be routinely provided is the number of forms that are missing according to the participant's testing and training schedule. Table 12 indicates how this information will be displayed.

In addition to the tables for the reports, the computer auditing system produces Quality Control (QC) reports that indicate the number of errors that were found on the individual forms. QC reports that are overly large will identify those sites requiring additional training on forms completion. A

monthly report summarizing data submission and problem identification for each site will be sent to the Study Chairperson so that she can monitor how the participating sites are doing.

Table 12. CS #2003 Number of Missing Forms by Site

# of Participants		Site				Total
		1	2	…	15	
Form 01	N					
	%					
Form 02	N					
	%					
.						
.						
.						
Form X	N					
	%					

I. Study Monitoring by the Data Monitoring Committee (DMC)

An independent oversight committee called the Data Monitoring Committee (DMC) will monitor study progress. This committee meets on the same basic schedule as the Study Group and Executive Committee, i.e., they will meet at 6 to 9 months after the start of patient recruitment and yearly thereafter. This committee does not usually meet during the last six months of a study. They will also meet once prior to the study startup to acquaint themselves with the study and to establish their procedures for reviewing and monitoring the study.

The major responsibility for the DMC members when they meet is to make a recommendation to the Director of the Cooperative Studies Program as to whether or not the study should continue. The study could be recommended for termination due to poor recruitment, treatment differences so large that it is possible to reach a final decision or treatment differences so small that continuation would be irresponsible. The DMC also reviews the participating sites' performance and makes recommendations concerning them. Data collected during the feasibility phase of the study, including site performance, verification of success rates in the control group and other operational measures, will be presented to the committee for its review and recommendations. Their final responsibility is to review all proposed protocol changes and sub-protocols and to make recommendations about their acceptability.

For the DMC to carry out its responsibilities, the CSPCC Study Team will provide the committee with a report approximately three weeks prior to their meetings. The report will consist of the tables described previously for the Study Group and Executive Committee reports as well as those

presenting baseline participant characteristics and outcome measures by treatment group. Differences between treatment groups on participant characteristics may indicate a need to use any significantly different characteristics as covariates for the outcome measures. Formal testing of the differences between treatment groups will be done at the study's conclusion. Analyses of variance techniques will be used to test characteristics that are continuous in nature, while chi-square techniques will be used for the discrete variable characteristics. The analyses of the primary and major secondary outcome measures are described above in section F. The results of these analyses will be provided in table format and will include the p-value from the chi-square analysis. Table 13 indicates how the MCS/VR-36 outcome measure will be displayed. Analyses of the SCI-QOL Physical Medical Health domain (three item banks for bladder management difficulties, bowel management difficulties and pain interference) and total body fat mass outcome measures will be presented in a similar fashion.

Table 13. CS #2003 Primary Outcome Measure: Mental Component Summary (MCS) of the VR-36

≥ 4-point improvement in MCS/VR-36 from baseline to end of 4-month intervention	SOC		EAW + SOC		Total		p-value
	n	%	n	%	n	%	
Success							
Failure							
Total							

Secondary outcome measures and analyses (as outlined in section G above) will also be provided in tabular format. These tables will present statistics for the outcome variables at all the time points at which the outcome measures are assessed: baseline, after the training/orientation Phase, two months into the 4-month intervention phase, and then again at the end of the intervention phase. Again using the MCS/VR-36 as an example, Table 14 indicates how these data may be presented. The p-values from the comparison of the difference scores (difference from baseline to the end of each time point) and from the mixed models analyses, as appropriate, will also be presented in these tables.

Table 14. CS #2003: VR-36 Mental Component Summary (MCS) Scores

	SOC				EAW + SOC			
Time Point	N	Mean	Std. Dev.	Median	N	Mean	Std. Dev.	Median
Baseline								
Post-Training/Orientation								
Two Months into Intervention Phase								
Post-Intervention Phase								

For the DMC to make its recommendation for continuation of the study, it will be necessary for them to see the analysis of the primary and major secondary outcome measures every time they are provided a report of study progress and it is possible to calculate these measures. Periodic monitoring of interim results can significantly affect the probability of making an incorrect decision (Type-I error rate). One interim look at the primary outcome measures (in addition to the final “look” at study’s end) will be proposed to the DMC for making the recommendation about whether or not to continue the trial or to stop for early efficacy. It is proposed that the one look occur when 80 (50%) patients have completed participation in the study. The O’Brien-Fleming method for alpha spending and rejection/acceptance boundaries will be suggested for Type-I error rate control. The O’Brien-Fleming boundaries for rejection (α) and acceptance (β) as well as the Type-I error rates for the two looks are given in Tables 15 and 16 for the MCS/VR-36 and SCI-QOL/PMH outcomes, respectively. The boundaries are presented graphically in Figures 10 and 11 for the two primary outcome measures.

Table 15. CS #2003: Two-sided O’Brien-Fleming Boundaries for Rejection (α) and Acceptance (β) – MCS/VR-36

Interim Look (Stage)	No. of Randomized Participants	Std. z (Rejection)		Std. z (Acceptance)		Type-I error
		Lower	Upper	Lower	Upper	
1	80	-3.12376	3.12376	-0.88945	0.88945	0.00089
2	160	-2.20883	2.20883	-2.20883	2.20883	0.01250

Figure 10. CS #2003 Combined Boundary Plot for MCS/VR-36

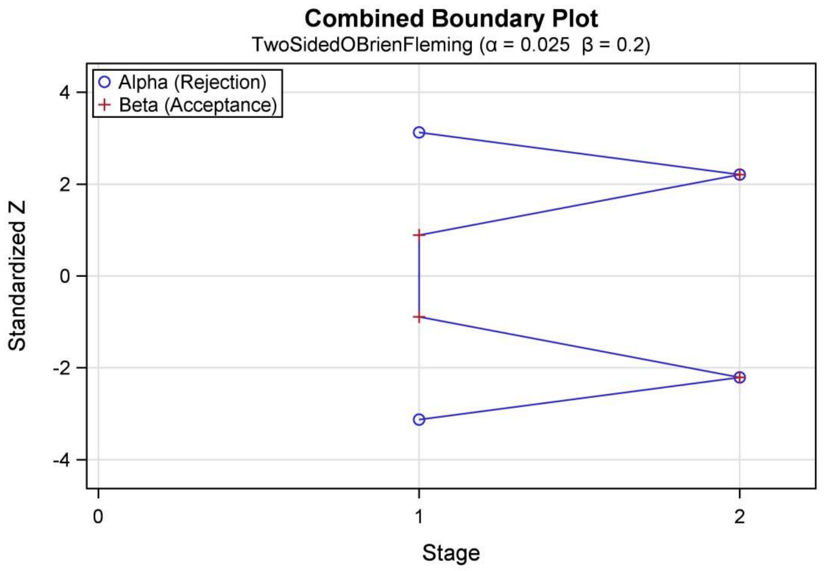

Table 16. CS #2003: Two-sided O'Brien-Fleming Boundaries for Rejection (α) and Acceptance (β) – SCI-QOL Physical Medical Health (PMH) domain

Interim Look (Stage)	No. of Randomized Participants	Std. z (Rejection)		Std. z (Acceptance)		Type-I error
		Lower	Upper	Lower	Upper	
1	80	-3.16956	3.16956	-0.40071	0.40071	0.00076
2	160	-2.24122	2.24122	-2.24122	2.24122	0.01250

Figure 11. CS #2003 Combined Boundary Plot for SCI-QOL Physical Medical Health (PMH) Domain

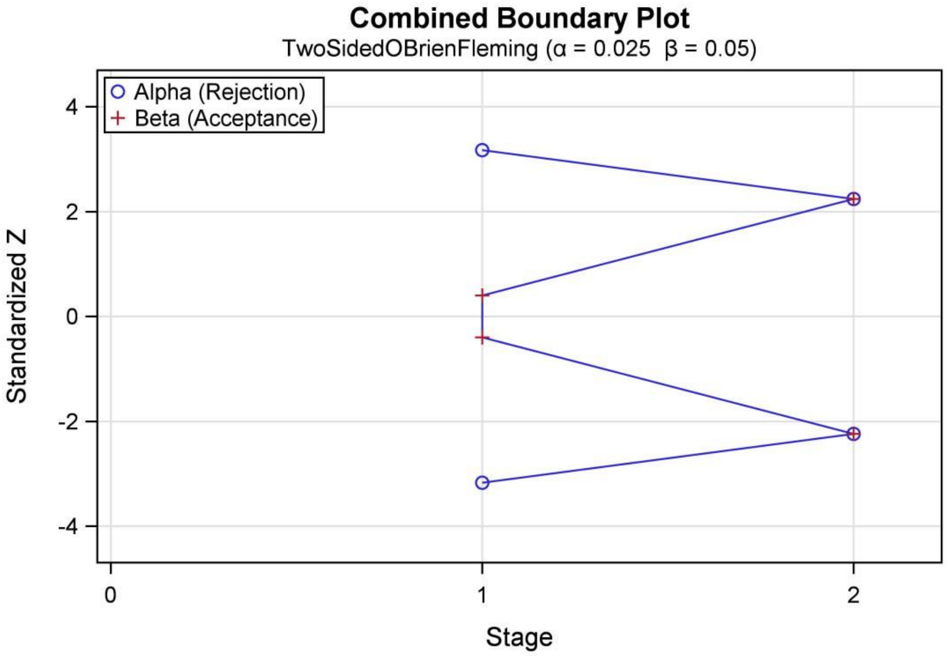

As with any clinical trial, the safety of the participants will be of utmost concern. Safety will be monitored closely and data will be collected on adverse events (AEs) and serious adverse events (SAEs) throughout the course of the study. All AEs and SAEs will be systematically recorded on case report forms and coded by the CSP Clinical Research Pharmacy Coordinating Center (CSPCRPCC) at Albuquerque using the MedDRA system. The MedDRA system employs standard terminology to present adverse events and to organize them by body system. The incidence of adverse events will be summarized for each treatment group and overall, by body system and MedDRA preferred term. The incidence differences between the control and intervention groups for each event will be tested using the Pearson chi-square test or Fisher's Exact test. Table 17 illustrates how AE data may be presented for this study.

Table 17. CS #2003 Cumulative Incidence of Adverse Events by Body System and MedDRA Term

Body System and Preferred Term	SOC (n = ?) n (%)	EAW + SOC (n = ?) n (%)	All Participants (N = ?) n (%)	p-value
All Adverse Events				
Subjects with at least one Adverse Event				
Body System 1				
Event 1				
Event 2				
Event 3				
.				
.				
.				
Body System 2				
Event 7				
Event 8				
Event 9				
Event 10				
.				
.				
.				
Body System N				
Event 14				
Event 15				
Event 16				
.				
.				
.				
Event N				

It is the responsibility of the CSPCC Study Team to provide the DMC with whatever information they feel they need to successfully monitor the study. Thus, additional tables will be added as required. In addition to the reports for the yearly meetings, the DMC will also be provided with reports between meetings at 6-month intervals.

XIV. QUALIFICATIONS

A. Site Qualifications

The Site Investigator is required to be a SCI staff physician. S/he will be responsible for the initial contact with any potential participants. Once a potential participant expresses verbal interest, additional study team members may continue the informed consent and eligibility screening process. The Site Investigator will perform the History and Physical (H&PE) and INSCSCI examinations. If there is skin breakdown or an abrasion due to contact friction with the exoskeleton, the Site Investigator/Physician works with the Trainers and participant to determine if the participant needs to take time off to allow it to heal or if the addition of padding or equipment adjustments is sufficient to prevent further breakdown and the participant may continue in the training sessions. Additional, co-Investigators who are physicians are welcome to help defray some of the workload from the Site Investigator; however, the Site Investigator will ultimately be responsible for overseeing all conduct related to the CS #2003 at their site. Co-Investigators from other disciplines are also welcome to be part of this study. Each site will need to provide a clear description of additional co-Investigator roles in the study.

The proposed study will require fifteen sites. Each site will have four full time staff who would be solely dedicated to the conduct of the study (1 ReWalk Trainer, 2 Assistant ReWalk Trainers and 1 Study Coordinator). Two staff persons are needed during the initial EAW Training phase for each of the participants. The ReWalk Trainer will have a clinically relevant professional background such as a biomedical, electrical or mechanical engineer, physical therapist, exercise physiologist, occupational therapist, nurse, athletic trainer, or equivalent. The ReWalk Trainer will be responsible for the screening of participants, scheduling, fitting and training of the exoskeleton, and mobility measurement aspects of the protocol. The ReWalk Trainer and Assistant ReWalk Trainers will be responsible for the administrative paperwork, assist with eligibility screening, assist with the informed consent process, scheduling of participants, the home evaluation when needed, the EAW training, weekly and monthly participant contact during the orientation and home use phases of the study, ensuring the Usual Activity log and EAW Activity Log are completed, reporting adverse events, and all other aspects of the study protocol. The Assistant ReWalk Trainers will assist the trainer in all EAW aspects of the study. All of the Trainers must be capable of assisting with the EAW training and as such, are expected to be healthy and free of back or other medical problems that would be contraindicated for lifting and carrying 60 pounds across a room. The Trainers must be able to lift the exoskeleton from one chair to another, or to place one in or take it out of the trunk of a car or van.

CS #2003 study will also support a Site Coordinator. The Site Coordinator will be responsible for the administration of the QOL outcome surveys, local Research & Development and CIRB

application paperwork and assisting with scheduling and data entry. The study Coordinators may be physical therapists, PT assistants, exercise physiologists, nurses, nurse assistants, research health science specialists or technicians, or other comparable disciplines that have experience with research methods and procedures and experience and knowledge about persons with SCI. Approved and classified PDs will be provided to the sites for recruitment of study personnel if requested.

Each Site will need the following:

1. Access to a bone mineral densitometer or space for the ones to be provided by CS #2003;
2. Secure space for storage of the ReWalk units;
3. Space for overnight battery charging of the ReWalk units;
4. Space for training participants to walk in the units (e.g., hallways, sidewalks, etc.);
5. Space for desk or office for study personnel (x4);
6. Access to the VA car pool;
7. Secure space for study files;
8. Dedicated study VA computer;
9. Evidence of medical center support; and
10. Access to a fax machine.

B. Site Participation

An invitation letter and brief description of the CS #2003 was sent to each of the SCI Service Chiefs to establish level of interest and available resources. Eighteen potential study sites responded with interest in participating in this study. Fifteen sites have been selected as the primary sites (Table 19). Criteria for site selection included: interest in being part of the study, the number of SCI patients seen in the catchment area, the number who use the site for outpatient visits, CSP NODE status, geographic location, availability of a bone mineral density (BMD) scanner for the BMD criteria, hospital space for the ReWalk training, secure storage of the devices, study files, and availability of staff desk and computer space. Additionally, in an effort to place sites at VAMCs in the geographic sections of the whole US, geographic location was used as a selection factor. Having more than 400 SCI patients seen annually in the outpatient clinic and being a Node were the two highest priority selection criteria, followed by geographic location.

Table 19. CSP #2003 Study Sites

	VA Medical Center and NODE Site	Site Investigator	Email	Type of Scanner	iDXA Status	# SCI Veterans in catchment	# SCI Veteran outpatient visits/y
1	**Boston**	Sunil Sabharwal, MD	sunil.sabharwal@va.gov	Hologic (Medical Center)	iDXA needed	1200	500
2	**Richmond**	Lance L. Goetz, MD	lance.goetz@va.gov	DXA (SCI Service)	iDXA needed	1400	501
3	**Tampa**	Kevin T. White, MD	kevin.white2@va.gov	Hologic (Medical Center)	iDXA needed	1200	820
4	**Houston (Node)**	Sally Ann Holmes, MD	SallyA.Holmes@va.gov	iDXA (Medical Center)	upgrade only	610	400
5	**Palo Alto (Node)**	Doug Ota, MD	Doug.Ota@va.gov	iDXA (SCI Serevice)	upgrade only	1028	400
6	**Long Beach (Node)**	Alice Jennifer Hon, MD	alice.hon3@va.gov	Hologic (Medical Center)	iDXA needed	773	275
7	**Minneapolis (Node)**	Byron Eddy, MD	Byron.Eddy@va.gov	Hologic (Medical Center)	iDXA needed	700	175
8	**Dallas (Node)**	Bridget R. Bennett, MD	Bridget.Bennett@va.gov	Hologic (Medical Center)	iDXA needed	900	700
9	**Milwaukee**	Denis F. Castillo, MD	Denis.Castillo@va.gov	Hologic (Medical Center)	iDXA needed	433	394
10	**St. Louis**	Katherine C. Stenson, MD	Katherine.Stenson@va.gov	iDXA (Medical Center)	upgrade only	433	394
11	**Augusta**	Michael Priebe, MD	Michael.Priebe@va.gov	iDXA (Medical Center)	iDXA needed	1200	900
12	**San Antonio**	Michelle Trbovich, MD	Michelle.Trbovich@va.gov	GE Lunar (Medical Center)	iDXA needed	451	240
13	**Bronx**	Stephen Kornfeld, DO	Stephen.Kornfeld@va.gov	iDXA (SCI Serevice)	iDXA needed	711	333
14	**Albuquerque**	Nancy Cutter, MD	Nancy.Cutter@va.gov	iDXA (Medical Center)	iDXA needed	500	400
15	**Cleveland**	Mary Kristi Henzel, MD, PhD	Mary.henzel@va.gov	iDXA (Medical Center)	iDXA needed	1200	400
					**Sub Total**	**13,606**	**7,022**

C. Office of the Chair Qualifications

CS #2003 has two study principal proponents, Dr. Ann M. Spungen (Chair) and Dr. William A. Bauman (Co-Chair). The Chair's Office is requesting two National Coordinators and a Staff Assistant. The two National Coordinators will perform separate and distinct functions to ensure successful completion of the clinical trial. One FTE would be for a biomedical engineer at the GS-13 level, and s/he will have significant experience and be an expert in conducting ReWalk fitting, training and troubleshooting; this person would oversee all training and standardization with the exoskeletal device. The Co-National Coordinator (1.0 FTE) would be at the GS-12 level, and s/he must have significant experience in performing and teaching the administration of the SCI-QOL and other participant-reported measures of quality of life. This person would oversee the standardization across sites for all of the QOL assessment tools proposed to be administered and ensure that the Site Coordinators are trained in the administration of these assessment tools. The Staff Assistant (GS-9, 1.0 FTE) will assist the Chair's Office with administrative paperwork, travel paperwork, and scheduling of site training. Complete descriptions of the Chair's Office staff responsibilities may be found in the position descriptions in the Budget Justification section.

XV. PLANS TO ASSURE SECURITY AND CONFIDENTIALITY OF STUDY DATA

Each participant will be assigned a participant number and unique alpha code for the case report forms and source documents. A code key (screening log) will be maintained at each investigational site. The participant number will be assigned as they are consented in the study. The site number along with their participant number will uniquely identify that person; however, it will not contain any personal identifiable information.

Data collection which contains any protected health information (PHI) such as informed consent forms, Health Insurance Portability and Accountability Act (HIPAA) forms, and contact information will be stored within each individual site and will remain either in locked cabinets or electronically behind the VA firewall in a password-protected file. Only approved study personnel will be permitted access to view study related folders. In addition, access to the study folders stored on the VA network will be restricted to personnel listed on this study. This will further limit access to files stored on the VA network that contain PHI and outcome data related to this study.

SharePoint will be utilized within this study as it has been considered an approved collaboration environment and access to the SharePoint site can be restricted by the study team as needed.

SharePoint will be utilized as an intranet portal to maintain documents, manage files and other collaborative efforts securely. All data will be sent to the Coordinating Center at Perry Point using DataFax which is a system to send case report forms using a fax machine to Perry Point which will maintain the data repository for all sites. To ensure that all questions are answered on the QOL outcome assessments (VR-36, SCI-QOL, Global Impression of Change Scale (Participant- and Companion-rated), PROMIS Sleep Disturbance, SCI-FI, and bowel function surveys), the site's study staff will conduct in-person or telephone interviews. Testing and survey procedures will be conducted in private rooms and training will be performed in rooms and hallways of the VA SCI Services, on the medical center grounds when weather conditions permit, at Community Based Outpatient Centers (CBOCs) under the same FWA number as the VA facility conducting the research, and/or in the participant's home or community.

XVI. DATA MANAGEMENT AND DATA SECURITY PLANS

Most data collection will be non-sensitive data that will be coded using a subject number that will link the data to a specific subject. Data collection may also take place through secure messaging using My HealtheVet. The code to the subject number will be stored in a separate secure location within the VA Network at each site. Access to sensitive data will be restricted to authorized users; access will be related to VA-approved research, data users and custodians. VA and non-VA staff will be aware that they must immediately report theft or loss of VA sensitive data or media containing VA sensitive data to the site VA Information Security Officer (ISO). Electronic VA sensitive data will be collected, used and stored on VA computers on the VA network (behind the VA firewall), on restricted access drives, and in password protected files. Hard copies of VA sensitive data will be collected and used and stored in locked cabinets in locked rooms. Any code linking identities to data will be stored separately from the data, electronically behind the VA firewall or hard copy in a locked cabinet in a locked room. Special access will be granted to study-related data to the sponsor and regulatory boards upon request. This will include representatives of the Food and Drug Administration (FDA), the Central Institutional Review Board (CIRB), Office of Research Oversight (ORO), and compliance officers, etc. Written documentation of any review of study related files will be required. This documentation will be stored with the study related paperwork. All subjects that are enrolled in the CS #2003 study will be informed of any potential review by these regulating bodies during the consent process.

Training for data collection and management will be conducted for all sites prior to initiating the study in order to ensure that the data is collected in the same manner and all study staff understand

the source documents and case report forms. Data from the sites will be sent to the Coordinating Center, Perry Point, MD. All hard copies of case report forms collected will be stored within locked cabinets at the sites. Electronic data will be stored on servers protected by the VA firewall. This study will utilize SharePoint as a method of maintaining documents and collaborating across the other VA sites. Restricted and selective access can be granted administratively to the SharePoint environment in order to prevent unauthorized access.

QOL outcome assessments (VR-36, SCI-QOL, Global Impression of Change Scale (Participant- and Companion-rated), PROMIS Sleep Disturbance, SCI-FI, and bowel function surveys) will be completed by each participant during an in-person or phone interview by a member of the study site staff. The interviewer will record the answers, making sure that all questions are answered. This data will be sent to the CSPCC via a Datafax system.

XVII. PLANS FOR DISSEMINATION OF STUDY RESULTS

A. Publication of Research Results

It is the policy of the Cooperative Studies Program not to reveal outcome data to Site Investigators until the data collection period of the study is complete. This policy is meant to prevent possible biases that might affect data collection. Members of the DMC will be reviewing outcome results to ensure that the study will be terminated early if a treatment is identified as prohibitively dangerous or if a definitive answer is reached prior to the scheduled study termination date.

All presentations and publications resulting from this study will follow CSP policy as specified by the CSP guidelines. The presentation or publication of any or all data collected by Site Investigators on patients entered into a Department of Veterans Affairs Cooperative Study is under the direct control of the study's Executive Committee. No individual Site Investigator has the right to use the study's data to perform analyses or interpretations, or to make public presentations or seek publication of any or all of the data without specific approval of the Executive Committee.

The Executive Committee has the authority to establish any number of publication committees, which usually will comprise of subgroups of Site Investigators and some members of the Executive Committee, for the purpose of producing manuscripts for presentation and publication. Any presentation or publication related to this study should be circulated to the Executive Committee for review, comments and suggestions at least four weeks prior to submission of the manuscript to

the presenting or publishing body.

All publications must give proper recognition to the funding source and should list all study participating site personnel (not necessarily as authors of the manuscript). If an investigator's major salary support and/or commitment is from the VA, it is obligatory that the investigator lists the VA as his/her primary institutional affiliation. Submission of manuscripts or abstracts must follow the usual VA policy; ideally, a subtitle states, "A Department of Veterans Affairs Cooperative Study." The CSP also requires that every manuscript be reviewed and approved by the CSPCC Director prior to submission as a final quality control step. Mechanisms for appeal by an investigator will follow procedures defined by the VA Office of Research and Development. Participation in a Department of Veterans Affairs Cooperative Studies Program clinical trial is voluntary. Any investigator who cannot accept these operational guidelines regarding publication policy should not volunteer to participate in the study.

B. Planned Publications

The Executive Committee, led by the study Chair, Dr. Spungen, on behalf of the study group, will prepare the primary outcome manuscript and investigators with special interest will prepare the secondary outcome manuscripts in each of these areas, as to be determined by the Executive Committee. The Executive Committee will consider additional ideas for sub-study manuscripts from members participating in the study. Sub-study activities (within the scope of this original protocol) may be authorized by the Executive Committee to address other important research questions raised.

<u>Primary publication:</u> Upon completion of the study, a manuscript will be prepared that focuses on the primary outcome, i.e. the proportion of subjects demonstrating a clinically significant change in MCS/VR-36 and the patient-reported outcomes for SCI-QOL bladder management difficulties, bowel management difficulties and pain interference, as compared between the Intervention and Control groups. This proportion of participants who achieved the major secondary outcome for total body fat mass changes will also be reported in the primary manuscript. Any adverse events will be reported as well.

<u>Other publications:</u> Upon completion of the study and the acceptance of the primary manuscript for publication, the secondary outcomes will be analyzed for the PROMIS sleep disturbance, bowel function survey, the remaining items in the SCI-QOL that were not included in the primary

outcomes, the physical function domains from the SCI-FI, and lipid profile results. The amount of time spent in the device and location of use will be reported in conjunction with any of the above manuscripts.

XVIII. HUMAN SUBJECTS PROTECTION CONSIDERATIONS

A. Recruitment of Participants and Special Considerations

The targeted study population to be studied is Veterans or military members with SCI (>6 months) who are non-ambulatory and a primary wheelchair user for mobility. The Site Investigator is required to be a SCI staff physician. S/he will be responsible for the initial contact with any potential participants. Once a potential participant expresses interest and gives verbal permission to learn more, additional study team members will assist the Site Investigator with the informed consent and eligibility screening process. There are no race, ethnicity or gender limitations for enrollment. The age range is limited to who are at least 18 years of age. There is no upper age limit because if someone passes all the inclusion and exclusion criteria, being of an older age, but in good health should not be exclusionary in of itself. Special classes of subjects such as pregnant women, prisoners, institutionalized individuals, or other populations that may be considered vulnerable will be excluded from this study with the exception of economically and educationally disadvantaged persons. There are Veterans and military members who are economically, educationally or socially disadvantaged individuals and they will be given the opportunity to participate in this research project. These disadvantaged persons will be given the same treatment as individuals who are not considered to be vulnerable.

B. Inclusion of Women and Minorities in Study

Consistent with the Belmont Report, "Ethical Principles and Guidelines for the Protection of Human Subjects," and Congressional legislation, eligible women and minorities will be included in this study.

C. Description of the Consenting Process

Consent will be obtained prior to study enrollment by the Site Investigators or a member of the study research team. Study team members will be trained to obtain consent. They will be responsible for explaining the proposed clinical trial and answering questions before obtaining

informed consent. The consent will be explained in its entirety by the study team. The terms of the consent form will be explained prior to obtaining the individual's signature. Individuals will be given the opportunity to take the consent home for review with family members (if they so choose), to think about it overnight, and to ask questions prior to signing the consent form. Participants will be asked to explain back to the study team member what they believe the study entails, thus giving the opportunity for the study team member to correct any misunderstandings or add further detail. Potential participants will be informed of their right to ask questions at any time during the trial and/or to withdraw at any time. The potential participants will be informed that choosing to not participate or to withdraw will not infringe on any of their regular VA benefits or medical care.

The timing of the consent process will take place prior to any screening assessments. The location of the consent process will primarily be in the site's area where the research will be conducted but is not limited to these areas. Additional locations where the consent process could take place include a physician's office, the investigator's offices, and/or designated rooms where privacy can be maintained (to include Community Based Outpatient Centers (CBOCs) under the same FWA number as the VA facility conducting the research). Additional locations could include the participant's home or community space where privacy can be maintained.

During the consent process, the participant's privacy will be maintained by using a private room. Participants who wish to have a family member or a friend in attendance during the consent process may do so. Ample time for decision making will be provided and the potential participant will be allowed and encouraged to discuss the proposed clinical trial with anyone before making a decision.

There will not be a plan for the consent of the individual's Legally Authorized Representative because an inability to sign consent is exclusionary to this clinical trial.

*Consenting of the companions may be obtained at the time the participant consents or during the Screening Phase for the enrolled participants. Consenting of the companions may take place in person or assent may be obtained over the phone.

D. Participant Compensation

A potential barrier to recruitment is the travel and time commitment for persons with SCI to enroll

in this study. A small stipend of $30 per visit to the SCI Centers has been included in the budget to help defray some of the expense and inconvenience of travel for participants in this study. During Screening, stipends will be given for up to 3 visits for screening/baseline evaluations (max. $90) and up to 5 days for EAW basic training (max. $150). At JJPVAMC, during the pilot study, it was found that participants were highly motivated to attend the 3 times weekly exoskeletal-assisted walking sessions and compliance was greater than 90%. Reimbursement for transportation and lodging may be made available to participants and companions who live more than 45 miles away from the SCI Centers. The amount will vary based on distance and will be arranged in advance with the study team.

E. Safety Considerations

Trainers will be able to identify symptoms and signs of autonomic dysreflexia (AD), such as rapid increase in blood pressure, flushing in the face, sweating above the level of injury; or symptoms of orthostatic hypotension, such as paling of the face and participant reporting lightheadedness. Study Trainers will have a policy in place at their facility in order to address these types of emergencies. These policies should include a place where the participant can be brought to a sitting position or safely laid down on the floor. Once they are in a safe position, the cause of the problem can be assessed, and appropriate steps can be taken. If there is an “error” in the ReWalk device, the Trainers will assist the participant to a sitting position using the graceful collapse feature. It will be strongly recommended that participants receiving anticoagulation therapy wear a helmet during use of the exoskeleton.

F. Foreseeable Risks

The potential risks associated with having a person with SCI dynamically load in an upright position and walk overground with a powered exoskeleton include: hypotension, low-impact fracture, falls resulting in bone fracture, muscle soreness or tear, joint damage, or other musculoskeletal injury, skin breakdown, and/or AD.

To date the investigative staff at the JJPVAMC has trained 12 participants to use the ReWalk system. In these initial 12 participants, there were no study related SAEs. One participant fell as a result of undetected water on the floor, but was not injured from the fall. There were minor skin abrasions (reported as study-related adverse events), which were resolved with additional padding and alterations in the fitting of the device. The group who initially studied the ReWalk at Moss

Rehabilitation Hospital, Philadelphia, PA reported their adverse events [43]. Esquenazi et. al., reported the following adverse reactions during his phase 1 clinical trial performed at Moss Rehabilitation Hospital, Philadelphia, PA: "(1) skin abrasions in areas of contact with the device, (2) lightheadedness, and (3) edema of the lower limbs [43, 83]. They were all managed by the appropriate use of (1) foam and padding, (2) caffeinated beverage intake and adjustment of blood pressure medication, (3) elastic stockings and rest, respectively. There were no detrimental changes in vital signs or complaints of lightheadedness with prolonged standing." Additional hazards to walking overground using a robotic exoskeleton include the risk of equipment malfunction, battery dying, or any unknown or unpredictable problem.

There are also some risks associated with the measurements obtained by the dual x-ray absorptiometry (DXA) scanner and the bilateral calcaneus x-rays. These medical diagnostic devices emit small amounts of radiation, approximately 8µSv (DXA) and 2µSv (bilateral x-rays). This amount of radiation is similar to normal background radiation amounts from radon, food, water, and cosmic radiation. There are risks associated with phlebotomy which include pain, swelling, bruising or skin irritation at the site of the venous puncture and rarely, syncope in persons who are susceptible to a vasovagal response. There are risks associated with anticoagulation therapy if a participant were to fall and hit their head. It will be strongly recommended that participants receiving anticoagulation therapy wear a helmet during use of the exoskeleton.

G. Risk Management and Emergency Response

All potential participants will be examined, and their medical charts will be reviewed by the study team physician (who is also a member of the study site's medical center staff) before being cleared for participation. Study personnel will be in attendance at all times while a participant is using the exoskeleton and while all testing is being performed. Blood pressure and heart rate will be monitored before, during, and after the training sessions, primarily to identify changes in blood pressure. The study staff will query the participants throughout each session for early signs or symptoms of hypotension such as feeling light-headed or queasiness. During the sessions, persons with a fall in blood pressure from their pre-session seated BP of >20/10 mmHg will be seated or brought to a supine position. The staff involved with this study will be trained to look for signs and symptoms of AD. Most persons with SCI are also knowledgeable about AD, but all participants will also be made aware of these signs and symptoms of AD. The legs where the straps are fastened will be examined for skin breakdown during (as needed) and after training sessions to ensure that there are no areas that may be abrading. If evidence of skin redness,

irritation or breakdown exists, alternate placements for straps and/or padding will be used to protect the area. Participants who have continued breakdown will be temporarily discontinued. Participants will be checked after each session for signs of edema. If edema is present, participants will be instructed to elevate their feet, use compression socks for the next session, and/or reduce the upright time or training frequency. If edema persists more than one day, the participant will be referred to the Site Physician. Training sessions will be discontinued if need be.

The ReWalk powered exoskeleton has safeguards built within the device to prevent or minimize injury to the user. The system can detect if there is a mechanical or electrical problem and automatically causes the device to remain in the standing position, preventing the user to walk in the system. The only movement the device will be able to do is sit down so that the user can be removed from the system safely. If there is a mechanical error while in the sitting position, the device will not allow the user to stand in the system, and there will be a sound alerting the person that there is a problem. If there is a sudden loss of power while standing or walking, there is a safety mechanism that will stiffen the joints of the exoskeleton passively and cause the joints to rotate slowly so that the person can have a "graceful" collapse to the floor without causing them harm. The "graceful" collapse function allows time for a protective response with the arms to reach for the ground. The ReWalk exoskeleton is instrumented with two power sources; one main battery which has power for up to 4 hours of continuous walking and an additional back up battery which has power for up to an additional 30 min of walking. The participant, companion and study team members will be instructed to ensure that the power source of the system is fully charged prior to any use of the ReWalk.

To prevent tripping over obstacles, the areas used for the sit-to-stand, stand-to-sit, walking, and stepping will be checked before and during each session to be free of any obstacles and water on the floor. There are risks associated with anticoagulation therapy if a participant were to fall and hit their head. It will be strongly recommended that participants receiving anticoagulation therapy wear a helmet during use of the exoskeleton. To prevent a potential fall, participants will not be progressed to walking until they have mastered the basic skills set. Additionally, all participants will be spotted by at least one study team member (and/or their companion once trained) while using the ReWalk and never left unattended.

Additional efforts to minimize the risk of fracture will be done by excluding those veterans with BMD in the extremely low range. Measurement of the BMD is part of the screening and eligibility criteria for participation in this study. Those potential participants who have low bone mass will

be excluded from the study (please see inclusion/exclusion criteria for details). There is risk associated with radiation exposure. The BMD assessment has a small amount of radiation exposure from the DXA scan. Measurements of two areas for BMD (hips and knees) will be performed one time for screening. The total body scan will be performed at baseline and will be repeated three additional times during the study. The following is a list of radiation exposure associated with each scan area obtained in this study.

Scan Type	Entrance Dose	Effective Dose	Scan Time
Hips	329 μGy	12.3 μSv	212 sec
Knee	34 μGy	unavailable	54 sec
Total Body	6 μGy	8.62 μSv	739 sec
Foot x-ray	unavailable	2.0 μSv	<1 sec

Values of radiation absorption (i.e. Effective Dose) for knee scans do not exist at this time. However, considering both the amount of radiation emitted and the scan time is lower than that of the other scans, the absorbed amount should also be lower than that of the hips (GE Medical Systems, Madison WI). The estimated sum quantity of radiation exposure from all of these DXA measurements combined is <35 μSv of absorbed radiation. This measurement is minimal even when compared to a routine chest x-ray which has an approximate dose of 60 μSv. In addition, the average person in New York City receives approximately 3000 μSv/year. Therefore, these tests would need to be repeated 85 times to receive an equivalent dose.

H. Emergency Care Plan

This study will be conducted in the VA hospitals at fifteen sites, in the CBOCs under the same FWA as the sites, or in the participant's home/community. During site visits and when training is conducted in the VA hospitals, the VA Hospital's emergency department would be called in the event of a medical emergency. If a participant is injured as a result of this study, the hospital site will be responsible for their medical care and the costs of this care. If a participant is seriously injured in their home or community environment during training or when using the device in their home, the study team and the participant (or their companion) will be instructed to call 911.

I. Potential Benefits

The information gained from this study may be scientifically useful to the field of SCI medicine.

The individual may experience improvements in QOL, but this is not guaranteed. An additional benefit of this study is to share the knowledge gained with the SCI Clinical Services in the VA system.

The participant will have both the participant version of the Informed Consent and HIPAA forms to sign. The Companion will have the option to sign the companion version of the Informed Consent and HIPAA authorization or provide assent over the phone. Only demographic information and AE / SAE information will be collected on the companions. A sample version of these five documents may be viewed in the Human Rights section.

XIX. TRANSITION OF PARTICIPANTS

The long-term aim of this study is to acquire information and share the newfound knowledge and insights with the clinical SCI Services. This information will assist the SCI Services to gain the necessary skills to develop and provide exoskeletal-assisted walking programs in-house with the anticipation of providing home prescription to interested and eligible Veterans and military members with SCI. It should be noted that before clinical staff can train patients at their facilities in the use of the exoskeleton, they will receive specific instruction/training in the use of the device.

In addition to having a truly immediate translation to clinical care, our study will actually make possible the clinical use of the exoskeleton by training local professional staff to become sufficiently skilled in the training of Veterans and military members with SCI who wish to use this technology for upright ambulation. In summary, clinical staff at the study sites will have the opportunity to directly participate in exoskeletal training, which will provide hands-on experience that will be readily transferrable to clinical care. This study will provide important clinical information, such as who are the best candidates for home use of an exoskeleton, the average number of sessions needed to pass the advanced skills test, the level and completeness of SCI that is related to successful use of the device, how often and in what locations the exoskeletons should be used, and any unexpected adverse events or serious adverse events, if they should occur, associated with home use. The prosthetics and orthotics departments will gain the opportunity to learn about the system and how to fabricate additional padding, if needed, for participants in addition to setting up contracting for purchasing these types of devices. There are other exoskeletons which will become available. The experience that the facilities gain from participation in this study will be a basis to identify the important features these new systems may offer to improve clinical care provided to veterans with SCI. Perhaps most importantly for the

clinical services, is the question of there being unexpected adverse events or serious adverse events associated with home use as well as how much is this device actually being used in the home/community environment.

XX. IMPACT OF THE SARS-COV-2 (COVID-19) PANDEMIC ON VETERANS WITH SPINAL CORD INJURY

This section has been added to the protocol to identify the impact of the COVID-19 pandemic on the screened participants from CS #2003. The questions to be asked are unrelated to the original research design and outcomes of CS #2003. A baseline questionnaire will be completed with screened participants as well as a follow-up questionnaire and a blood draw. These additions are at no additional cost to the study. All costs will remain budget neutral within each site. Although a budget for each site will be provided, it is understood that each site will cover these costs within the approved budget for their site.

A. Background

In the United States (US), the first case of the 2019 novel coronavirus disease (COVID-19), caused by the severe acute respiratory syndrome SARS-CoV-2 virus, was reported on January 19, 2020 (Holshue, et.al., NEJM March 2020). As of May 26, 2020, the US had 1,697,361 confirmed cases with 99,462 deaths and in the Veteran's Affairs Health system, there had been 13,327 positive cases and over 1,133 deaths. The numbers of positive cases and deaths in the cities where the VA CS #2003 study sites are located is provided (Table 20).

Table 20. Number of COVID-19 Positive Cases and Related Deaths by City as of May 26, 2020

VA Site Location		# of Positive Cases	# of Deaths
1	Boston	12,511	618
2	Houston	10,995	221
3	Long Beach	1,582	73
	Los Angeles	46,018	2,116
4	Palo Alto	2,602	139
	San Francisco	2,386	40
5	Richmond	1,546	118
6	Tampa	1,939	72
7	St. Louis	4,690	387
8	Dallas	8,998	211
9	Minneapolis	7,168	539
10	Milwaukee	6,535	257
11	Albuquerque	1,347	68
12	Augusta	529	18
13	Bronx	43,252	3,473
	New York City	203,569	20,740
14	Cleveland	3,963	217
15	San Antonio	2,449	69

There is limited information available on how Veterans with SCI have been affected by COVID-19. The most severe and direct negative impact for Veterans with SCI would be to contract COVID-19. Indirect negative impacts may also stem from the loss of caregiver or home attendant services, difficulty accessing routine healthcare, maintaining

wheelchairs, assistive and/or adaptive equipment, and other inconvenient and potentially harmful disruption of services that may be occurring. Positive indirect benefits may also be occurring, such as increased access to internet-based activities.

B. Rationale

Veterans with SCI are often immunosuppressed and have chronic health conditions (e.g. cardiovascular disease, respiratory compromise, metabolic disease including diabetes mellitus) that place them at a potentially higher risk of complications and more severe symptoms should they contract COVID-19. Additionally, individuals with SCI may be debilitated from poor nutrition, experience chronic pressure injuries with or without osteomyelitis, have concurrent respiratory infections, and experience frequent urinary tract infections (UTI). Of note, individuals with higher cord lesions who have difficulty clearing secretions from their lungs because of an impaired or absent cough due to respiratory muscle weakness from paralysis are at a higher risk of developing pneumonia and its complications. The signs and symptoms of COVID-19 may be different in individuals with SCI than the general population. For example, the high fevers characteristic of COVID-19 in the general population may not be observed in persons with tetraplegia, who experience lower core body temperatures, thereby requiring a different definition of fever for symptom surveillance and clinical diagnosis. Also, the common feeling reported of chest pressure may be absent in those who are insensate in this area. Persons with respiratory compromise, may experience shortness of breath earlier in the course and decline more rapidly, an indication for earlier admission and closer surveillance.

In the Veterans Health Administration (VHA) and the Spinal Cord Injury and Disease (SCI/D) National Registry, there are 17,806 Veterans with SCI. It is not known how many Veterans with SCI are, or will be, directly infected by this virus, nor the severity of their symptoms and survival rates. However, it is anticipated that, due to their high risk of immunodeficiency and other co-morbidities, the occurrence may be higher, with the potential for more severe symptoms and greater mortality than in the general population.

Additionally, of great concern, is the anecdotal report of loss of services, including routine medical care for non-COVID-related conditions (i.e. pressure injuries, urinary tract infections, etc.), loss or interruption of caregiver services, and wheelchair or assistive devices repair options, that has been an unfortunate consequence of the pandemic. Mr. James Weisman, President and CEO of

United Spinal Association, Inc. (a membership of 58,000 nationwide which includes about 1500 Veterans with SCI) stated on March 30, 2020: "*Homecare workers, always in short supply, are staying home watching their kids, self-isolating because of potential exposure--or exposure, and traveling via public transportation to patients and potentially exposing them. More than ever, it has become obvious that adequate homecare services are a fundamental necessity of the SCI community in order to stay out of nursing homes and hospitals…. And many are replacing caregivers with family members out of fear they will become infected from the caregiver*".

The VA Cooperative Study Program's CS #2003 is able to use the existing study staff to conduct a baseline survey/medical chart review, a follow-up survey and a blood draw. The survey would be used to determine the participant-reported direct medical effects of COVID-19 (with the use of the medical record when and if information is available) and the indirect psychosocial and environmental impacts of COVID-19 on Veterans with SCI who have been screen-consented for CS #2003. This knowledge would provide a "snapshot" of what is occurring across the nation in Veterans with SCI and will enable us to plan to meet the immediate needs of the Veteran SCI community as the course of the pandemic becomes known across the US. This information will be important to the VHA and the SCI/D National Service to be able to better prepare for future pandemics and other natural disasters. Immediate benefit may be the identification of Veterans with SCI who are in urgent need of services. This information would be relayed back to the medical center providers. As of the date of the administrative hold for CS #2003 on March 16, 2020, more than 400 Veterans with SCI had been screened for participation across 15 VA sites. A list of the number screened by site is provided (Table 21).

Table 21. Number of Veterans with SCI Screened by Site

	Site	Count
1	Boston	34
2	Houston	28
3	Long Beach	48
4	Palo Alto	37
5	Richmond	35
6	Tampa	41
7	St. Louis	34
8	Dallas	31
9	Minneapolis	34
10	Milwaukee	36
11	Albuquerque	10
12	Augusta	16
13	Bronx	11
14	Cleveland	15
15	San Antonio	13
	**Total Screened**	**423**

The infrastructure for VA CS #2003 is in place with funding on stations through September 30, 2020 and a full budget (4 staff per site at 14 sites) is requested for fiscal year (FY) 21 (Houston was withdrawn as a site in 2018, but those screened participants can be contacted by the San Antonio site staff). VA CS #2003 was put on a voluntary administrative hold on March 16, 2020. CS #2003 study staff will be trained to conduct the surveys and medical chart reviews while other

clinical research is on hold and to continue to conduct the surveys once the administrative hold has been lifted. The administrative hold has been lifted for the COVID-19 survey and blood draw.

The initial phone or in-person survey serves to capture COVID-19 information from the Veterans with SCI themselves that may not be in the medical record. This survey includes SCI-specific questions related to demographics, neurological characteristics and any loss of services during the pandemic. For example, information will be recorded such as the amount of assistance provided before the pandemic, the loss or reduction in home care services since the pandemic, other potentially negative consequences, and potential positive consequences such as offering of and inclusion in more internet-based social activities or telehealth medical services. The phone survey will be the primary method of collecting the information, with the option to complete the survey in person.

A medical chart review will be used for retrieving medical history and COVID-19 medical information when applicable (some Veterans with SCI may not be treated at their VA hospital, but rather at a local non-VA hospital, in which case this medical information may not be available to the study staff). The chart review will consist of information from the Spinal Cord Injury and Disease (SCI/D) National Office for COVID-19, using the “Coronavirus Disease 2019 SCI Screen (COVID-19 SCI Screen)” and other COVID-19-related medical records, if applicable. The medical records of Veterans with SCI may contain the COVID-19 SCI Screen information and will be recorded on the survey. Any additional medical information will be recorded as appropriate.

A follow-up “in-person” visit to update information from the initial survey and to collect blood samples for virus testing, anti-viral antibody, and other immune analyses relevant to the COVID-19 disease will be planned at least one month after the initial survey. The antibody, cytokine, and other immunological analyses at a future date when our country has recovered from this current pandemic will be critical for determining the number of Veterans with SCI who were infected with the virus, the relationship with the signs and symptoms of infection, if any, and the rate of survival in this population sample. There will also be an option to complete the follow-up survey over the phone for participants who do not wish to travel to the local VAs. An option to complete the blood draw in the home will be available through the services of a VA contracted phlebotomist vendor, ExamOne.

The initial survey and follow-up survey, medical chart review, and blood draw are intended to

capture the following information:

Questions related to the **direct impact of COVID-19** on Veterans with SCI will be collected by phone interview using Doximity. Doximity allows for a registered physician (or delegate) to log in and set a phone number, such as each local VA Medical Center who would be represented in this study. The person receiving the call sees the VA Medical Center as the caller ID. Participants will also have the option to complete the initial survey by in-person interview.

In Veterans with SCI, the following information will be collected (but not limited to):

a) The number who are/were diagnosed with COVID-19, determined from the medical records and/or interviews;
b) The number who presented with symptoms, determined from the medical records and/or interviews;
c) The type, duration and severity of symptoms, determined from the medical records and/or interviews;
d) The number who have anti-SARS-CoV-2 antibodies, with testing to be performed at least one month after initial interview (or whenever is deemed safe for an in-person visit to the medical centers); and
e) The number and characteristics of those who survived.

Questions related to the **indirect impact of COVID-19** pandemic on Veterans with SCI who rely on caregiver assistance and other services that may have been interrupted during the COVID-19 outbreak will be determined via interview.

The following information will be collected (but not limited to):

a) The number who report any caregiver assistance;
b) The relationship to the person(s) who provides the assistance (family, friend, aide, healthcare worker, etc.);
c) The number whose caregivers live with them;
d) The number whose caregivers travel to their homes;
e) Adverse health consequences that resulted from reduced or lack of caregiver assistance
    a. Any clinically relevant/significant health event during the study period, such as: pressure injury, UTI, fracture, or other,
    b. Lack of access to in-person medical services when requested or needed (in other words-is the overwhelmed healthcare system also affecting their more general access to care?).
b) Other adverse consequences of the COVID-19 pandemic on persons with SCI, such as:

a. Wheelchair maintenance/repair interruption,
b. Prescription renewal interruption,
c. Social isolation (lack of interactions with others),
d. Became unemployed, or
e. Other

Questions related to the potential <u>positive consequences of the COVID-19</u> pandemic would also be explored for:

a) Participation in virtual support groups (i.e. chat groups, other)
b) Participation in virtual exercise/physical therapy programs
c) Participation in virtual recreation therapy programs, or
d) Other.

Most of the survey questions have been developed with fixed-format answers, with as few open-ended questions as possible.

C. Methods

<u>Participants:</u> Veterans with SCI who were initially screened for CS #2003 will be contacted to determine willingness to be consented for participation in the survey and medical chart review and to be consented for the follow-up survey and blood draw at least one month from the initial survey.

The goal is to interview as near to 100% of the 400 plus Veterans with SCI as possible. We believe it is possible to recruit as many as 90% (approximately n=381), but to be conservative, we are expecting 80% (approximately n=338). Eighty percent (80%) is realistic because the sites' staff already know these Veterans with SCI, many of whom would be motivated to know if they have anti-SARS-CoV-2 antibody, will have missed their annual physicals during the pandemic, and will already be scheduled, or in the process of scheduling, to visit their VAMC. (In these cases, the CS #2003 staff would coordinate with the SCI/D service whenever possible to schedule both visits at the same time.)

<u>Baseline/Initial Survey:</u> Two survey forms will be used to collect the medical chart information and the responses from the participant. The medical chart review contains SCI-specific questions to record SCI characteristics of the participants. The survey will also record the loss or reduction in home care services and other potentially negative or positive consequences.

Follow-up Visit: After the COVID-19 national crisis has subsided, a follow-up visit (at least one month after the initial survey) will be scheduled at the VA Medical Centers for any of the Veterans with SCI who participated in the initial survey. During this follow-up visit, the participant will have the option to complete a blood draw and follow-up survey. Additionally, any of the over 400 Veterans with SCI who present to the VA Medical Centers who had not received the initial contact and interview would also be eligible to be interviewed in-person and for the blood draw. If participants complete the follow-up survey, they will receive a stipend of $50. No additional funding is being requested from CSP for this stipend. All costs will remain budget neutral within each site.

Blood collection: A single blood draw for a total of 20.0 mL of blood for the following will be performed:

1. To be analyzed at each site's local VA laboratory:
    a. One red top tube (6.0 mL) for c-reactive protein (CRP), spun down for serum and separated into 4.0 mL for the local lab and 2.0 mL to be frozen and shipped to the Chair's Office for antibody testing.
    b. One purple top tube (6.0 mL) for complete blood count with differential (CBC w/Diff) as whole blood (not spun down) for the local lab.
    c. Note: Each site will need a laboratory agreement to pay for the costs of the CBC w/Diff and CRP tests.
2. To be shipped to the Chair's Office for analyses:
    a. From the red top tube above, 2.0 mL, frozen serum for antibody testing
    b. One green top tube (5.0 mL) spun down for plasma and frozen for cytokine testing
    c. One PAX gene tube (3.0 mL) frozen as whole blood for transcriptional analysis

If participants complete the blood draw, they will receive a stipend of $50. No additional funding is being requested for this stipend. All costs will remain budget neutral within each site.

There will be an option to complete the blood draw at the participant's home through services provided by a VA contracted phlebotomist vendor. The vendor, ExamOne, will only provide the blood collection service. They will not be engaged in research activities. All questions about the study (other than blood collection visit schedules and blood draw procedures) will be directed to the local site team and/or the Chair's Office. ExamOne will spin down the samples, as appropriate, and deliver them to the local site personnel for freezing or local site lab analysis.

All blood samples will be labeled with the study ID, date and a coded participant ID number. The serum and plasma of each spun down tube and the PAX gene tube with whole blood will be stored in a freezer for shipment to the James J Peters VA Medical Center, Bronx, NY (Chair's Office). The cytokine testing will include: Interleukin-1 (normal range 0-5 pg/ml); Interleukin-6 (normal range 5-15 pg/ml); Interferon-alpha (normal range 0 to 35 U/L), Interferon-gamma (normal range <8.1 pg/mL), tumor necrosis factor (TNF)-alpha (normal range ≤ 2.8 pg/mL, and TNF-gamma (normal range <10 pg/ml).

The optimum methodology to perform the antibody analyses will be determined in consultation with the VHA Infectious Disease Service and the Clinical Laboratory at the time of sample analysis. In the future, biological values and other clinical data will be compared to other populations, including Veterans without SCI or non-Veterans with SCI.

<u>Timeline:</u> It is estimated the study staff can complete the initial surveys on screened participants by the end of September 2021. Once the medical centers have re-opened to outpatient visits, the in-person or phone visits for the follow-up surveys and in-person visits for blood draws can be completed by the end of FY21, which is within the timeframe of the completion of CS #2003. An estimated timeline is provided (Table 22).

Table 22. Timeline	FY20 Q3	FY20 Q4			FY21 Q1 to Q4			
	Jun-20	Jul-20	Aug-20	Sep-20	Q1	Q2	Q3	Q4
Administrative approvals[1]	✓							
CIRB submission		✓						
CIRB approval					✓			
Survey development & refinement	✓	✓						
iDatafax form for the survey			✓	✓	✓			
Train CS #2003 staff (via Teams or other)					✓			
Initial surveys and chart review						✓	✓	✓
Follow-up surveys, chart reviews & blood draws							✓	✓
[1]CS#2003 Executive committee and CSP VACO review and approvals								

D. Relevance

This information will be used to inform the VHA of the medical, sociological and/or quality of life impacts from the COVID-19 pandemic on Veterans with SCI who were screened for CS #2003. Specifically, for the medical impact, we will learn about the number in this sample who were

infected, their symptoms (if any) and severity, any associated illnesses, immunological responses, and the number of individuals who recovered and survived. The sociological and quality of life impact of this virus will also be reported, including the interruption of vital services such as caregivers, medical appointments, wheelchair repair, levels of anxiety, and other aspects. We will also learn about any positive occurrences that may be reported such as usage of telemedicine, and more access to online social groups or web-based activities. **Important benefits from this project will be to improve preparedness for future local and/or national disasters in order to protect Veterans living with SCI from untoward exposure and/or loss of services.**

Notes for consideration:

- On the follow-up visit, if the participant has a positive test for anti-SARS-CoV-2 antibodies, we will know that they had the COVID-19 infection at some time in the past (and even could have been asymptomatic); these individuals would be categorized as persons who have survived. If they test negative for anti-SARS-CoV-2 antibodies, then we will know they had not been infected as of the date of the test.
- Some of the Veterans with SCI who participate in the baseline/initial survey will have already been diagnosed with viral infection and/or COVID-19 (many of our institutions already have some Veterans with SCI who have tested positive for the virus). Some Veterans with SCI are being treated for COVID-19 based on severe symptoms and may not have been tested for viral infection by lab diagnostics until after they have been treated. Other individuals with less severe symptoms, that did not require hospitalization, may not have been tested for COVID-19 using lab tests. In these cases, the antibody testing will reveal how many people with SCI in our study sample had the virus.
- If a Veteran with SCI who was screened for our study has died during this time, the information about the death may be in the medical record at that Veteran's VA medical center and we will be able to capture it from chart review. However, it is recognized that information about those who did not survive will be the most difficult to get for many reasons: lack of documentation in the medical record, lack of definitive information about the cause of death, lack of a COVID-19 diagnosis, etc.
- If a Veteran with SCI who participates in the baseline/initial survey dies at some time afterwards, then upon follow-up, we may learn about the death and be able to acquire some of this information from medical records.

- The additional workload to conduct the survey and in-person visit with the blood draw will be able to be continued without interruption to the usual tasks and duties that are required for CS #2003.

E. Limitations

The screened participants for CS #2003 would have met the inclusion criteria for CS #2003. As such, the Veterans with SCI in this survey sample would have tended to be those with more function and, as a result, will be a biased sample. The generalizability for the results would therefore be limited to the demography of this sample.

If some, or all, of the Sites' VA medical centers have not re-opened by next year, the study staff could still complete the follow-up survey and chart reviews from telework. The survey is of value even without the blood draw since identification of adverse and/or positive aspects of the pandemic would be informative for future preparedness for Veterans with SCI.

Considerations for how the blood draws can safely take place will be discussed at that time and may include solutions such as staff in full Personal Protective Equipment (PPE) going to the Veterans home to take the blood sample.

XXI. REFERENCES

1. Glickman, S. and M.A. Kamm, *Bowel dysfunction in spinal-cord-injury patients.* Lancet, 1996. **347**(9016): p. 1651-3.
2. Cornell, S.A., et al., *Comparison of three bowel management programs during rehabilitation of spinal cord injured patients.* Nurs Res, 1973. **22**(4): p. 321-8.
3. Korsten, M.A., et al., *Difficulty with evacuation after spinal cord injury: Colonic motility during sleep and effects of abdominal wall stimulation.* J Rehabil Res Dev, 2004. **41**(1): p. 95-100.
4. Potter, P.J., *Disordered control of the urinary bladder after human spinal cord injury: what are the problems?* Progress in brain research, 2006. **152**: p. 51-57.
5. Markman, L.J., *Bladder and bowel management of the spinal cord injured patient.* Plast Surg Nurs, 1988. **8**(4): p. 141-5.
6. Saunders, L.L. and J.S. Krause, *Behavioral factors related to fatigue among persons with spinal cord injury.* Archives of physical medicine and rehabilitation, 2012. **93**(2): p. 313-318.
7. LaVela, S., et al., *Dysfunctional sleep in persons with spinal cord injuries and disorders.* Spinal cord, 2012. **50**(9): p. 682-685.
8. Johanson, J.F., et al., *Association of constipation with neurologic diseases.* Dig Dis Sci,

1992. **37**(2): p. 179-86.
9. Stone, J.M., et al., *Chronic gastrointestinal problems in spinal cord injury patients: a prospective analysis.* Am J Gastroenterol, 1990. **85**(9): p. 1114-9.
10. Kwok, S., et al., *Does regular standing improve bowel function in people with spinal cord injury? A randomised crossover trial.* Spinal Cord, 2015. **53**(1): p. 36-41.
11. Michailidou, C., et al., *A systematic review of the prevalence of musculoskeletal pain, back and low back pain in people with spinal cord injury.* Disability & Rehabilitation, 2013. **36**(9): p. 705-715.
12. Adams, M.M. and a.L. Hicks, *Spasticity after spinal cord injury.* Spinal cord, 2005. **43**: p. 577-86.
13. Jensen, M.P., et al., *Sleep problems in individuals with spinal cord injury: frequency and age effects.* Rehabilitation psychology, 2009. **54**(3): p. 323.
14. Fawkes-Kirby, T., et al., *Clinical correlates of fatigue in spinal cord injury.* Spinal Cord, 2007. **46**(1): p. 21-25.
15. Spungen, A.M., et al., *Soft tissue body composition differences in monozygotic twins discordant for spinal cord injury.* Journal of Applied Physiology, 2000. **88**(4): p. 1310.
16. Spungen, A.M., et al., *The effects of level and completeness of lesion on body composition in subjects with spinal cord injury.* J Spinal Cord Med, 1999. **22**: p. 43.
17. Spungen, A.M., et al., *Factors influencing body composition in persons with spinal cord injury: a cross-sectional study.* J Appl Physiol, 2003. **95**(6): p. 2398-407.
18. Wilmet, E., et al., *Longitudinal study of the bone mineral content and of soft tissue composition after spinal cord section.* Paraplegia, 1995. **33**(11): p. 674-7.
19. Gorgey, A.S. and D.R. Gater, *Regional and relative adiposity patterns in relation to carbohydrate and lipid metabolism in men with spinal cord injury.* Appl Physiol Nutr Metab, 2011. **36**(1): p. 107-14.
20. Bauman, W.A., et al., *The effect of residual neurological deficit on serum lipoproteins in individuals with chronic spinal cord injury.* Spinal Cord, 1998. **36**(1): p. 13-7.
21. Bauman, W.A., et al., *The effect of residual neurological deficit on oral glucose tolerance in persons with chronic spinal cord injury.* Spinal Cord, 1999. **37**(11): p. 765-71.
22. Bauman, A., *Carbohydrate and lipid metabolism in individuals after spinal cord injury.* Top Spinal Cord Inj Rehabil 2, 1997. **2**(4): p. 1-22.
23. Duckworth, W.C., P. Jallepalli, and S.S. Solomon, *Glucose intolerance in spinal cord injury.* Arch Phys Med Rehabil, 1983. **64**(3): p. 107-10.
24. Duckworth, W.C., et al., *Glucose intolerance due to insulin resistance in patients with spinal cord injuries.* Diabetes, 1980. **29**(11): p. 906-10.
25. Bauman, W.A., et al., *Is immobilization associated with an abnormal lipoprotein profile? Observations from a diverse cohort.* Spinal Cord, 1999. **37**(7): p. 485-93.
26. Brenes, G., et al., *High density lipoprotein cholesterol concentrations in physically active and sedentary spinal cord injured patients.* Arch Phys Med Rehabil, 1986. **67**(7): p. 445-50.
27. Massucci, M., et al., *Walking with the advanced reciprocating gait orthosis (ARGO) in thoracic paraplegic patients: energy expenditure and cardiorespiratory performance.* Spinal cord, 1998. **36**(4): p. 223-227.
28. Arazpour, M., et al., *Evaluation of a novel powered gait orthosis for walking by a spinal cord injury patient.* Prosthetics and orthotics international, 2012. **36**(2): p. 239-246.

29. van Hedel, H.J., *Gait speed in relation to categories of functional ambulation after spinal cord injury.* Neurorehabil Neural Repair, 2009. **23**(4): p. 343-50.
30. Waters, R.L. and B.R. Lunsford, *Energy cost of paraplegic locomotion.* J Bone Joint Surg Am, 1985. **67**(8): p. 1245-50.
31. Tefertiller, C., et al., *Efficacy of rehabilitation robotics for walking training in neurological disorders: a review.* J Rehabil Res Dev, 2011. **48**(4): p. 387.
32. Nooijen, C.F., N. ter Hoeve, and E.C. Field-Fote, *Journal of NeuroEngineering and Rehabilitation.* Journal of neuroengineering and rehabilitation, 2009. **6**: p. 36.
33. Harkema, S.J., et al., *Balance and ambulation improvements in individuals with chronic incomplete spinal cord injury using locomotor training–based rehabilitation.* Archives of physical medicine and rehabilitation, 2012. **93**(9): p. 1508-1517.
34. Forrest, G.F., et al., *Ambulation and balance outcomes measure different aspects of recovery in individuals with chronic, incomplete spinal cord injury.* Archives of physical medicine and rehabilitation, 2012. **93**(9): p. 1553-1564.
35. Roy, R.R., S.J. Harkema, and V.R. Edgerton, *Basic concepts of activity-based interventions for improved recovery of motor function after spinal cord injury.* Archives of physical medicine and rehabilitation, 2012. **93**(9): p. 1487-1497.
36. Graupe, D., et al., *Stochastically-modulated stimulation to slow down muscle fatigue at stimulated sites in paraplegics using functional electrical stimulation for leg extension.* Neurological research, 2000. **22**(7): p. 703-704.
37. Hirokawa, S., et al., *Energy expenditure and fatiguability in paraplegic ambulation using reciprocating gait orthosis and electric stimulation.* Disability & Rehabilitation, 1996. **18**(3): p. 115-122.
38. Solomonow, M., et al., *The RGO Generation II: muscle stimulation powered orthosis as a practical walking system for thoracic paraplegics.* Orthopedics, 1989. **12**(10): p. 1309-1315.
39. Durfee, W.K. and A. Rivard. *Preliminary design and simulation of a pneumatic, stored-energy, hybrid orthosis for gait restoration*. in *ASME 2004 International Mechanical Engineering Congress and Exposition*. 2004. American Society of Mechanical Engineers.
40. Durfee, W.K. and A. Rivard, *Design and simulation of a pneumatic, stored-energy, hybrid orthosis for gait restoration.* Journal of biomechanical engineering, 2005. **127**(6): p. 1014-1019.
41. Goldfarb, M., et al., *Preliminary evaluation of a controlled-brake orthosis for FES-aided gait.* Neural Systems and Rehabilitation Engineering, IEEE Transactions on, 2003. **11**(3): p. 241-248.
42. Petrofsky, J. and J.B. Smith, *Physiologic costs of computer-controlled walking in persons with paraplegia using a reciprocating-gait orthosis.* Archives of physical medicine and rehabilitation, 1991. **72**(11): p. 890-896.
43. Esquenazi, A., et al., *The ReWalk powered exoskeleton to restore ambulatory function to individuals with thoracic-level motor-complete spinal cord injury.* Am J Phys Med Rehabil. **91**(11): p. 911-21.
44. Zeilig, G., et al., *Safety and tolerance of the ReWalk exoskeleton suit for ambulation by people with complete spinal cord injury: a pilot study.* J Spinal Cord Med. **35**(2): p. 96-101.
45. *The ReWalk Exoskeletal Walking System for Persons With Paraplegia (Clinical Trials.gov NCT01454570)*. 2011 September 14, 2011]; Available from:

http://www.clinicaltrials.gov/ct2/show/NCT01454570?term=NCT01454570&rank=1.

46. Spungen AM, et al., *Exoskeletal-Assisted Walking for Persons with Motor-Complete Paraplegia*, in *In Force Sustainment: Rehabilitation, Regeneration and Prosthetics for Re-Integration to Duty*. 2013, North Atlantic Treaty Organization (NATO): Neuilly-sur-Seine, France: STO. p. 6-1, 6-14.
47. Fineberg, D.B., et al., *Vertical ground reaction force-based analysis of powered exoskeleton-assisted walking in persons with motor-complete paraplegia.* The journal of spinal cord medicine, 2013. **36**(4): p. 313-321.
48. ReWalkRobotics. Available from: http://rewalk.com/.
49. Spungen AM, A.P., Fineberg D, Harel NY, Kornfeld S, Bauman WA., *Beneficial changes in body composition after exoskeletal-assisted walking: Implications for improved metabolic function Top.* Spinal Cord Inj Rehabil, 2013. **19(5)**: p. 8-9.
50. Asselin PK, K.S., Kornfeld S, Cirnigliaro C, Agranova-Breyter I, Bauman WA, Spungen AM., *Heart Rate and Oxygen Demand of Powered Exoskeleton-Assisted Walking in Persons with Paraplegia.* Under Review.
51. EksoBionics. Available from: http://www.eksobionics.com/.
52. RexBionics. Available from: http://www.rexbionics.com/.
53. IndegoExoskeleton. Available from: http://indego.com/indego/en/home.
54. Administration, F.a.D., 2014.
55. Cella, D., et al., *The neurology quality-of-life measurement initiative.* Archives of physical medicine and rehabilitation, 2011. **92**(10): p. S28-S36.
56. *Patient Reported Outcomes Measurement Information System (PROMIS). Dynamic Tools to Measure Health Outcomes from the Patient Perspective* Available from: http://www.nihpromis.org.
57. *Quality of Life in Neurological Disorders (Neuro-QOL)*. Available from: http://www.neuroqol.org.
58. *PROMIS Instrument Development and Psychometric Evaluation Scientific Standards*. 2012; Available from: http://www.nihpromis.org/Documents/PROMIS_Standards_050212.pdf.
59. Kisala, P.A. and D.S. Tulsky, *Opportunities for CAT applications in medical rehabilitation: development of targeted item banks.* Journal of applied measurement, 2009. **11**(3): p. 315-330.
60. Tulsky DS, K.P., Victorson D, Choi S, Gershon R, Heinemann AW, Cella DF, *Methodology and Calibration of Item Banks to Measure Patient Reported Outcomes Following Spinal Cord Injury.* . Journal of Spinal Cord Medicine., Under review.
61. Tulsky DS, K.P., Vivtorson D, Tate DG, Heinemann AW, Charlifue S, Kirshblum SC, Fyffe D, Gershon R, Spungen AM, Bombardier CH, Dyso-Hudson TA, Amt,mann D, Kalpakjian C, Choi S, Jette A, Forchheimer M, Cella DF *Overview of the Spinal Cord Injury – Quality of Life (SCI-QOL) Measurement System.* Submitted to the Journal of Spinal Cord Medicine. .
62. Heinemann, A.W., et al., *Measurement Properties of the Spinal Cord Injury-Functional Index (SCI-FI) Short Forms.* Archives of physical medicine and rehabilitation, 2014.
63. Jette, A.M., et al., *Development and initial evaluation of the spinal cord injury-functional index.* Archives of physical medicine and rehabilitation, 2012. **93**(10): p. 1733-1750.
64. Tulsky, D.S., N.E. Carlozzi, and D. Cella, *Advances in outcomes measurement in rehabilitation medicine: Current initiatives from the National Institutes of Health and the*

National Institute on Disability and Rehabilitation Research. Archives of physical medicine and rehabilitation, 2011. **92**(10): p. S1-S6.

65. Tulsky, D.S., et al., *Spinal cord injury-functional index: item banks to measure physical functioning in individuals with spinal cord injury.* Archives of physical medicine and rehabilitation, 2012. **93**(10): p. 1722-1732.
66. Tulsky, D.S., et al., *Developing a contemporary patient-reported outcomes measure for spinal cord injury.* Archives of physical medicine and rehabilitation, 2011. **92**(10): p. S44-S51.
67. Tulsky, D.S. and M. Rosenthal, *Quality of life measurement in rehabilitation medicine: building an agenda for the future.* Archives of physical medicine and rehabilitation, 2002. **83**: p. S1-S3.
68. Tulsky, D., Kisala, PA, Victorson, D, Tate, DG, Heinemann, AW, Charlifue, S, Kirshblum, SC, Fyffe, D, Gershon, R, Spungen, AM, Bombardier, CH, Dyson-Hudson, TA, Amtmann, D, Kalpakjian, C, Choi, S, Jette, A, Forchheimer, M, & Cella, DF. , *Overview of the Spinal Cord Injury – Quality of Life (SCI-QOL) Measurement System.* . Journal of Spinal Cord Medicine, 2015: p. In press.
69. Buysse, D.J., et al., *Development and validation of patient-reported outcome measures for sleep disturbance and sleep-related impairments.* Sleep, 2010. **33**(6): p. 781.
70. Yu, L., et al., *Development of short forms from the PROMIS™ sleep disturbance and sleep-related impairment item banks.* Behavioral sleep medicine, 2012. **10**(1): p. 6-24.
71. Sloan, J.A., D. Cella, and R.D. Hays, *Clinical significance of patient-reported questionnaire data: another step toward consensus.* Journal of clinical epidemiology, 2005. **58**(12): p. 1217-1219.
72. Hooker, S.P. and C.L. Wells, *Effects of low- and moderate-intensity training in spinal cord-injured persons.* Med Sci Sports Exerc, 1989. **21**(1): p. 18-22.
73. Jacobs, P.L., et al., *Relationships of oxygen uptake, heart rate, and ratings of perceived exertion in persons with paraplegia during functional neuromuscular stimulation assisted ambulation.* Spinal Cord, 1997. **35**(5): p. 292-8.
74. Jenkinson, C., A. Coulter, and L. Wright, *Short form 36 (SF36) health survey questionnaire: normative data for adults of working age.* BMJ: British Medical Journal, 1993. **306**(6890): p. 1437.
75. Lucke, K.T., et al., *Quality of life in spinal cord injured individuals and their caregivers during the initial 6 months following rehabilitation.* Quality of Life Research, 2004. **13**(1): p. 97-110.
76. Westgren, N. and R. Levi, *Quality of life and traumatic spinal cord injury.* Archives of physical medicine and rehabilitation, 1998. **79**(11): p. 1433-1439.
77. Kazis, L.E., *The veterans SF-36 health status questionnaire: development and application in the Veterans Health Administration.* Medical Outcomes Trust Monitor, 2000. **5**(1): p. 1-2.
78. Kazis, L.E., et al., *Patient-reported measures of health: the Veterans Health Study.* The Journal of ambulatory care management, 2004. **27**(1): p. 70-83.
79. Kazis, L.E., et al., *Health-related quality of life in patients served by the Department of Veterans Affairs: results from the Veterans Health Study.* Archives of Internal Medicine, 1998. **158**(6): p. 626-632.
80. Johns, M.W., *A new method for measuring daytime sleepiness: the Epworth sleepiness scale.* sleep, 1991. **14**(6): p. 540-545.

81. Kleinman, L., et al., *Psychometric evaluation of the fatigue severity scale for use in chronic hepatitis C.* Quality of Life Research, 2000. **9**(5): p. 499-508.
82. Krupp, L.B., et al., *The fatigue severity scale: application to patients with multiple sclerosis and systemic lupus erythematosus.* Archives of neurology, 1989. **46**(10): p. 1121-1123.
83. Esquenazi, A., *New Bipedal Locomotion Option for Individuals with Thoracic Level Motor Complete Spinal Cord Injury*. Journal of the Spinal Research Foundation, 2013. **8**(1): p. 26-28.

STATISTICAL ANALYSIS PLAN (SAP)

I. INTRODUCTION

"Exoskeletal-Assisted Walking (EAW) in Persons with SCI: Impact on Quality of Life" (CS #2003), is a two-group, randomized clinical trial to compare wheelchair use as standard of care (SOC) for home and community mobility to an exoskeletal-assisted walking device (EAW) plus SOC (EAW+SOC) in veterans with chronic spinal cord injury (SCI) of greater than six months, for clinically meaningful improvements in quality of life (QOL) outcomes. Overall, the study will enroll 160 participants from 10 sites over a total recruitment period of three years. Enrollment will be accomplished by using a staggered start approach: six sites will be brought up in the first year with the remaining four coming on board a year later.

There are two primary outcome measures in this study: the first is the score on the Mental Component Summary of the Veterans Rand-36 (MCS/VR-36) and the second is the sum T-score for the SCI-QOL Physical Medical Health domain (three item banks for bladder management difficulties, bowel management difficulties and pain interference). The primary study hypotheses are: 1) 33% of the intervention group (EAW+SOC) compared with 10% of the control group (SOC) will demonstrate a clinically relevant change (improvement) of ≥ 4.0 points in the MCS/VR-36, for greater vitality and social functioning, and improved role-emotional and mental health, from baseline to the end of the intervention phase, and 2) 42% of the intervention group compared with 10% of the control group will demonstrate a clinically significant improvement from baseline of ≥ 10% in the sum T score, indicating improvement in patient reported outcomes for SCI-QOL bladder management, bowel management and pain interference.

A major secondary objective of the study is to demonstrate that participants who use the exoskeleton in addition to SOC (EAW+SOC) will have at least a 1.0 kg loss in total body fat mass by the end of the four- month intervention compared to those who receive SOC only. Other secondary objectives are to demonstrate that participants in the EAW+SOC group will have greater net improvements than participants in the SOC only group in the following outcomes: 1) Global Impression of Change (participant- and companion-rated), 2) disturbed sleep as measured by the T-score of the Patient Reported Outcomes Measurement Information System (PROMIS) Sleep Disturbance, 3) self-reported methods and measures of bowel function, 4) sum T scores of the SCI Functional Index (SCI-FI) physical function item banks, 5) sum T scores of the SCI-QOL Emotional domain, 6) sum T scores of the SCI-QOL Social Participation domain,
7) lipid profile for high density lipoprotein cholesterol (HDL-c), low density lipoprotein cholesterol (LDL-c), triglycerides (TG), and total cholesterol (TC), and 8) fasting plasma glucose (FPG) and insulin (FPI) levels for calculation of Homeostasis Model of Assessment-Insulin Resistance (HOMA-IR).

II. SAMPLE SIZE

Using preliminary data generated from the Exoskeletal-assisted Walking Program at the James J. Peters VA Medical Center, three separate power calculations were performed, one for each of the primary outcome measures, the MCS/VR-36 and the sum t-score of the SCI-QOL bladder, bowel and pain item banks, and one for the major secondary measure, total body fat mass. The sample size estimates are based on the

preliminary data from these three outcome measures as a proportion of participants who achieved a clinically significant change score (MCS/VR-36 and Total Body Fat Mass) or a clinically significant percent change (SCI-QOL PMH). Assuming the proportion in the control group achieving a clinically meaningful change to be 10% (or 0.100) for all three outcome measures, and estimating the attrition rate at 15%, the sample sizes range from a low of 71 per group (95% power) for the SCI-QOL PMH outcome to a high of 79 per group (80% power) for the MCS/VR-36 group. As a note about Table 1, to adjust for multiple outcome measures, a significance level of 0.025 was used in the sample size calculations for the co-primaries (MCS/VR-36 and SCI-QOL) and also for the major secondary outcome measure (Total Body Fat Mass). Based on the sample size and power calculations below, the study will enroll a total sample size of 160 participants, 80 per group.

**Table 1: Sample Size/Power Calculations**			
Two group continuity corrected χ^2 test of equal proportions (odds ratio = 1) (equal n's)	**MCS/VR-36**	**SCI-QOL (PMH)**	**Total Body Fat Mass**
**Test significance level, α**	0.025	0.025	0.025
**1 or 2 sided test?**	2	2	2
**Control proportion, π_1**	0.100	0.100	0.100
**Intervention proportion, π_2**	0.333	0.420	0.350
**Odds ratio, $\psi = \pi_2 (1 - \pi_1) / [\pi_1 (1 - \pi_2)]$**	4.493	6.517	4.846
**Power (%)**	80	95	85
**n per group**	67	60	66
**n per group with 15% attrition**	79	71	78

III. FEASIBILITY PHASE

In order to confirm the hypothesized control proportion for the two primary and major secondary outcomes, to determine the feasibility of recruiting 8 participants per site per year, and to assess other operational aspects of the study, a feasibility phase has been built into this study. The planned duration of the feasibility phase of the study is one year. Six out of ten planned sites (including five sites that are designated CSP NODES) will be brought up in the first year.

During the feasibility phase, several site start-up activities and operational aspects of the study will be examined. These activities will include the hiring of site personnel (including classification of position descriptions, position announcements and recruitment), the processes for procuring and distributing the ReWalk and iDXA devices to the sites, the methods by which site staff are trained on the use of the devices and on data collection and management activities, the number of sessions needed for participants in the intervention group to pass the Advanced Skills Test, the practicality of Veterans with SCI to find motivated and eligible walking companions, and attrition (drop-out) rates. The lessons learned during this phase will be implemented during activation of the remaining participating sites.

Recruitment rates at the six active sites will be closely monitored during this phase. The initial six sites are each expected to enroll 8 participants in the first year of the study, for a total of 48 participants at the end of the first year. The actual recruitment experience of the six sites in the first year will be used to revise the projected recruitment rate for the remainder of the study and may lead to a lengthening or shortening of the total recruitment period, as appropriate.

Besides the verification of the assumed recruitment rate and other site start-up activities, the feasibility phase also provides an opportunity to assess the assumed success rates in the control (SOC) group. As stated above in section II, we have hypothesized that 10% (0.100) of the control group will have a clinically meaningful change in the outcome measures for sample size/power calculations. Ten percent is thought to be a conservative estimate as there is no reason to expect the control group to have clinically meaningful changes in quality of life measures or in total body fat mass while continuing to receive SOC for their spinal cord injury. However, as a means of verifying the control proportion of 10% (or 0.100), the proportion of participants considered successful in the control group for all three outcomes and 95% confidence intervals for the proportions will be reviewed upon the completion of 25% (40) of the randomized participants. Based on the extensive experience with this population it is highly unlikely that the assumed success rates in the control group (SOC) will be any higher than the assumed 10%.

At the completion of the feasibility phase, the study will be moved to a continuation phase where the remaining sties (4 remaining sites) will be activated, and the remaining participants (120 remaining participants) will be recruited and followed up unless very different (much higher than 10%) success rates in the control group are observed.

IV. BASELINE COMPARABILITY

Baseline comparability between the treatment groups will be evaluated with respect to such variables as demographics (e.g., age, gender, and race) and baseline values of outcome measures (MCS/VR-36, SCI- QOL, total body fat mass, etc.). Chi-square and analysis of variance techniques, as appropriate, will be used to determine any differences in distribution of the variables across the treatment groups. Any variable that appears to be different between the groups ($p<0.10$) will be considered as a potential covariate in statistical analyses.

V. GENERAL OUTCOME ANALYSIS GUIDELINES

All statistical tests will be 2-sided. The two primary and major secondary outcome measures will be tested at a 0.025 level of significance. Because of the large number of secondary outcomes to be analyzed, all other secondary outcomes will be tested at a significance level of 0.01 to maintain control over Type I error. SAS 9.4 will be used to conduct all the statistical analyses. A variety of analytic methods will be used for the primary endpoints, secondary endpoints and other analyses.

VI. ANALYSIS OF PRIMARY AND MAJOR SECONDARY OUTCOME MEASURES

The primary analysis will be based on an intent-to-treat (ITT) model. All participants who are randomized will be included for both groups, whether they are study completers (participants who complete the study through the four-month intervention phase) or early terminators (drop-outs). In order to test the hypotheses for the primary and major secondary outcome measures, each randomized participant will be deemed a success or failure at attaining: 1) ≥ 4.0 point improvement in the MCS of the VR-36, 2) ≥ 10% improvement in the SCI-QOL bladder management difficulties, bowel management difficulties and pain interference sum T score, and 3) ≥ 1 kg. total body fat mass loss. For each of the outcomes, the proportion of participants successful at achieving a clinically significant improvement will be compared between the two groups (Intervention vs. Control) using a chi-square analysis. These will constitute the primary and major secondary efficacy analyses. Participants who drop out will be treated as failures. As such, by design, there will be no missing data: participants either meet the outcome criteria (successes), do not meet these criteria (failures) or they drop out (also failures).

VII. SECONDARY ANALYSES

In addition to the primary analysis, several secondary analyses will be performed and are described below.

A. Analyses will be performed that include only those participants who complete the study (excluding drop-outs):

    1. Chi-square analyses of the primary and major secondary outcome measures (proportion of successes) will be repeated for only those participants who complete the 4-month intervention phase.

    2. Analyses of the MCS/VR-36, SCI-QOL, total body fat mass loss, Global Impression of Change and other secondary outcome measures will include comparisons of the mean difference scores (change from baseline to the end of the intervention phase) of these outcome variables using t-tests.

B. Interim time points of data collection for all outcome variables have been incorporated into the study design. These time points are: baseline, five-session EAW Basic Skills Test, after training/orientation phase, two months into the intervention phase, and then again after four months of the intervention phase (primary and major secondary outcomes main time point). Secondary analyses of the MCS/VR-36, SCI-QOL, total body fat mass loss, Global Impression of Change and other secondary outcome measures will include comparisons of the mean difference scores (change from baseline to the end of each time point) of these outcome variables using t-tests. Other secondary analyses of outcome measures will be performed that use all data available. For these continuous variables, repeated measures analysis of covariance will be used to analyze them, with baseline scores, plus any variables determined from the baseline comparisons, being used as covariates in the analyses. SAS MIXED and GENMOD procedures, which allow for the use of incomplete data sets, will be used if it can be safely assumed that missing data are missing at random. If this assumption is not valid, a multiple imputation strategy of the missing responses will be implemented which does not rely on the

assumption that missing data occur at random. This will allow an intent-to-treat approach for the statistical analyses, where complete cases are not required.

C. An analysis to determine the characterization of the drop-outs will be performed by using descriptive and correlation statistics. The reasons for drop-out and the number of sessions/time points completed will be described. A correlation analysis will be performed with the reasons for drop-out and the demographic variables and other potential variables to identify characteristics of persons who drop out of the study.

VIII. MONITORING OF STUDY BY STUDY GROUP AND EXECUTIVE COMMITTEE

The Study Group (all of the Site Investigators) and Executive Committee will meet 6 to 9 months after patient recruitment begins and at annual intervals thereafter until the end of the study. Three weeks prior to these meetings and at 6-month intervals between the meetings, these groups will be provided a report that will allow them to assess study progress. Since both groups are composed of study participants, no outcome data will be provided in these reports. The information provided will include data on:

A. Screening, enrollment and retention
B. Patient background characteristics at entry
C. Data quality and protocol adherence.

A. Screening, Enrollment and Retention

During the pre-screening phase of the study, potential participants will be first contacted by the Site Investigator through chart reviews, other physician referrals and/or pre-existing knowledge about their patients. Eligible and interested potential participants will be contacted by the Site ReWalk Trainers and/or Site Study Coordinators for initiation of the consenting process and the screening and baseline evaluations. Any participant who consents to the study and passes all screening and baseline evaluations is eligible to be randomized into the study.

The progress of patient accrual will be presented to the monitoring groups in three formats. Table 2 gives the first format, which presents, by site, the actual number of patients entered into the study, the expected number of patients to be entered at the time of the report, and the percent of expected that were entered. The table will allow the Executive Committee to determine which sites are not recruiting as expected and the Site Investigators to see how their sites are doing in comparison to the others. The second format, Table 3, shows the number of participants entered into the study by month. Again these data will be organized by site. This table will indicate if recruitment is improving or worsening over time at the various sites. Sites where intake is worsening can be detected and the Site Investigator contacted to identify the reason for the recruitment deficit.

TABLE 2. CS #2003 Number of Participants Randomized vs. Expected

Site	Number Randomized	Number Expected	Percent of Expected
1			
2			
3			
4			
5			
6			
7			
8			
9			
10			
**Total**			

TABLE 3. CS #2003 Number of Participants Entered Each Month by Site

Month	Site 1	Site2	…	Site 10	Total
June 2016					
July 2016					
August 2016					
September 2016					
.					
.					
.					
**Total**					

Finally, the intake data will be plotted over time for the total number of participants recruited as in Figure 1. An expected intake line is given for comparison purposes, assuming a staggered start of sites: 6 sites enrolling 48 participants in the first year of recruitment, the full complement of 10 sites enrolling during the second year of recruitment (80 participants) and 4 sites enrolling 32 participants during the third year, for a total of 160 participants randomized.

FIGURE 1. CS #2003 Observed vs. Expected Recruitment

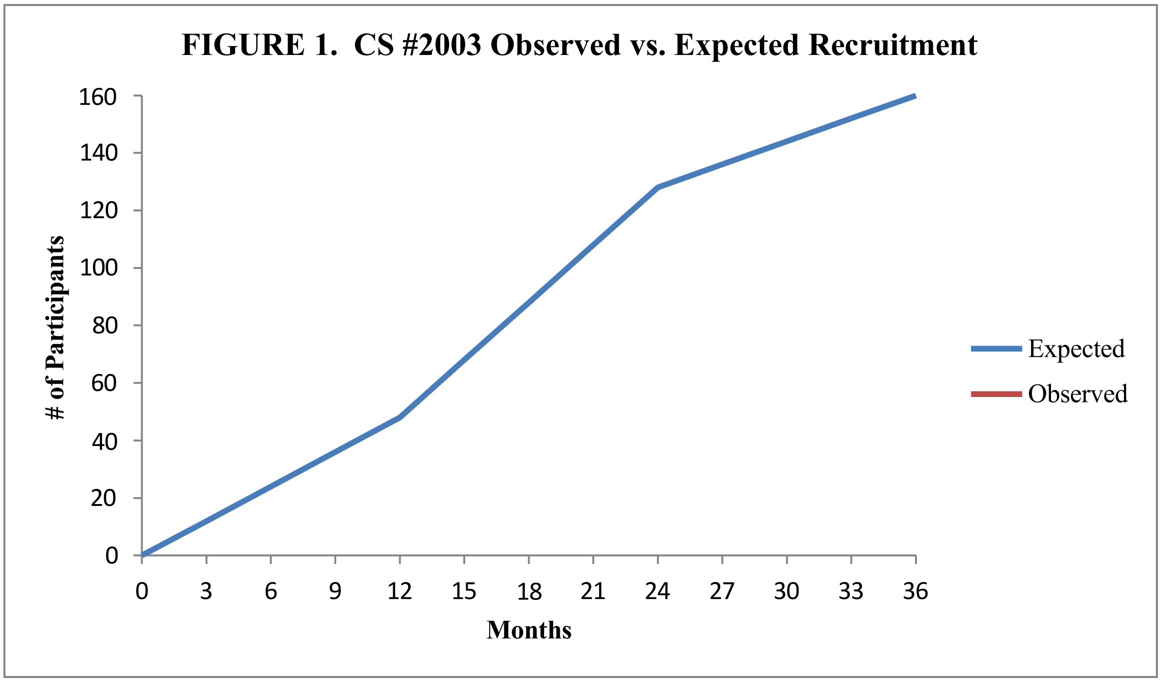

The number of participants screened and the number of those that randomize in the study will be presented in Table 4. The reasons for the exclusion of screened participants will be presented in Table 5.

TABLE 4. CS #2003 Cumulative Screening Summary: All Participants by Site

Site	Screened	Excluded	Randomized	% Excluded
1<br>2<br>3<br>.<br>.<br>10				
**Total**				

TABLE 5. CS #2003 Summary of Ineligibility: Primary Reason for Exclusion, All Sites

Primary Reason for Exclusion	# Screened	# Excluded	% of Screened
Less than 18 years of age			
Duration of SCI less than 6 months			
.			
.			
.			
No companion to assist at home			
Inability to provide informed consent			
Diagnosis of neurological injury other than SCI			
Progressive condition likely to result in changing neurological status			
.			
.			
.			
Psychopathology documentation that may conflict with study objectives			
Pregnancy or women planning to become pregnant during study			
**Total**			

B. Background Characteristics at Entry

Background characteristics of study participants are collected on the Screening/Baseline and History & Physical Forms. Tables summarizing the important background characteristics by site will be prepared and submitted to the Study Group so they will have an idea of the population being studied and comparisons of enrollment among the sites can be made. This information will be presented as means and medians for continuous variables and as frequency tables for discrete variables. Table 6 shows how the continuous variable age will be presented. Other variables that will routinely be presented will include gender, race,

education and smoking history. Analysis of variance and chi-square techniques will be used to identify any statistically significant differences that may exist among the sites.

TABLE 6. CS #2003 Age Statistics by Site

Site	N	Mean (Years)	Standard Deviation	Median
1				
2				
.				
.				
.				
10				
**Total**				

C. Data Quality and Protocol Adherence

The final type of information that will be given to the Site Investigators is data that will allow them to assess the quality of the data being submitted as well as how well the sites are adhering to the protocol. These data will be given by site, so sites performing substantially below average can be identified and remedial action taken to improve their performance.

One piece of information that will be routinely provided is the number of forms that are missing according to the participant's testing and training schedule. Table 7 indicates how this information will be displayed.

In addition to the tables for the reports, the computer auditing system produces Quality Control (QC) reports that indicate the number of errors that were found on the individual forms. QC reports that are overly large will identify those sites requiring additional training on forms completion. A monthly report summarizing data submission and problem identification for each site will be sent to the Study Chairperson so that she can monitor how the participating sites are doing.

TABLE 7. CS #2003 Number of Missing Forms by Site

# of Participants		Site				Total
		1	2	…	10	
Form 01	N					
	%					
Form 02	N					
	%					
.						
.						
.						
Form X	N					
	%					

IX. STUDY MONITORING BY THE DATA MONITORING COMMITTEE (DMC)

An independent oversight committee called the Data Monitoring Committee (DMC) will monitor study progress. This committee meets on the same basic schedule as the Study Group and Executive Committee, i.e., they will meet at 6 to 9 months after the start of patient recruitment and yearly thereafter. This committee does not usually meet during the last six months of a study. They will also meet once prior to the study startup to acquaint themselves with the study and to establish their procedures for reviewing and monitoring the study.

The major responsibility for the DMC members when they meet is to make a recommendation to the Director of the Cooperative Studies Program as to whether or not the study should continue. The study could be recommended for termination due to poor recruitment, treatment differences so large that it is possible to reach a final decision or treatment differences so small that continuation would be irresponsible. The DMC also reviews the participating sites' performance and makes recommendations concerning them. Data collected during the feasibility phase of the study, including site performance, verification of success rates in the control group and other operational measures, will be presented to the committee for its review and recommendations. Their final responsibility is to review all proposed protocol changes and sub-protocols and to make recommendations about their acceptability.

For the DMC to carry out its responsibilities, the CSPCC Study Team will provide the committee with a report approximately three weeks prior to their meetings. The report will consist of the tables described previously for the Study Group and Executive Committee reports as well as those presenting baseline participant characteristics and outcome measures by treatment group. Differences between treatment groups on participant characteristics may indicate a need to use any significantly different characteristics as covariates for the outcome measures. Formal testing of the differences between treatment groups will be done at the study's conclusion. Analyses of variance techniques will be used to test characteristics that are

continuous in nature, while chi-square techniques will be used for the discrete variable characteristics. The analyses of the primary and major secondary outcome measures are described above in section V. The results of these analyses will be provided in table format and will include the p-value from the chi-square analysis. Table 8 indicates how the MCS/VR-36 outcome measure will be displayed. Analyses of the SCI- QOL Physical Medical Health domain (three item banks for bladder management difficulties, bowel management difficulties and pain interference) and total body fat mass outcome measures will be presented in a similar fashion.

Table 8. CS #2003 Primary Outcome Measure: Mental Component Summary (MCS) of the VR-36

≥ 4-point improvement in MCS/VR-36 from baseline to end of 4-month intervention	SOC		EAW + SOC		Total		p-value
	n	%	n	%	n	%	
Success							
Failure							
Total							

Secondary outcome measures and analyses (as outlined in section VI above) will also be provided in tabular format. These tables will present statistics for the outcome variables at all the time points at which the outcome measures are assessed: baseline, after the 5-session EAW Basic Skills Test, after the training/orientation Phase, two months into the 4-month intervention phase, and then again at the end of the intervention phase. Again, using the MCS/VR-36 as an example, Table 9 indicates how these data may be presented. The p-values from the comparison of the difference scores (difference from baseline to the end of each time point) and from the mixed models analyses, as appropriate, will also be presented in these tables.

Table 9. CS #2003: VR-36 Mental Component Summary (MCS) Scores

	SOC				EAW + SOC			
Time Point	N	Mean	Std. Dev.	Median	N	Mean	Std. Dev.	Median
Baseline								
Post-EAW Basic Skills Test								
Post-Training/Orientation								
Two Months into Intervention Phase								
Post-Intervention Phase								

For the DMC to make its recommendation for continuation of the study, it will be necessary for them to see the analysis of the primary and major secondary outcome measures every time they are provided a report of study progress and it is possible to calculate these measures. Periodic monitoring of interim results can significantly affect the probability of making an incorrect decision (Type-I error rate). One interim look at the primary outcome measures (in addition to the final "look" at study's end) will be proposed to the DMC for making the recommendation about whether or not to continue the trial or to stop for early efficacy. It is

proposed that the one look occur when 80 (50%) patients have completed participation in the study. The O'Brien-Fleming method for alpha spending and rejection/acceptance boundaries will be suggested for Type-I error rate control. The O'Brien-Fleming boundaries for rejection (α) and acceptance (β) as well as the Type-I error rates for the two looks are given in Tables 10 and 11 for the MCS/VR-36 and SCI-QOL/PMH outcomes, respectively. The boundaries are presented graphically in Figures 2 and 3 for the two primary outcome measures.

Table 10. CS #2003: Two-sided O'Brien-Fleming Boundaries for Rejection (α) and Acceptance (β) – MCS/VR-36

Interim Look (Stage)	No. of Randomized Participants	Std. z (Rejection)		Std. z (Acceptance)		Type-I error
		Lower	Upper	Lower	Upper	
1	80	-3.12376	3.12376	-0.88945	0.88945	0.00089
2	160	-2.20883	2.20883	-2.20883	2.20883	0.01250

Figure 2. CS #2003 Combined Boundary Plot for MCS/VR-36

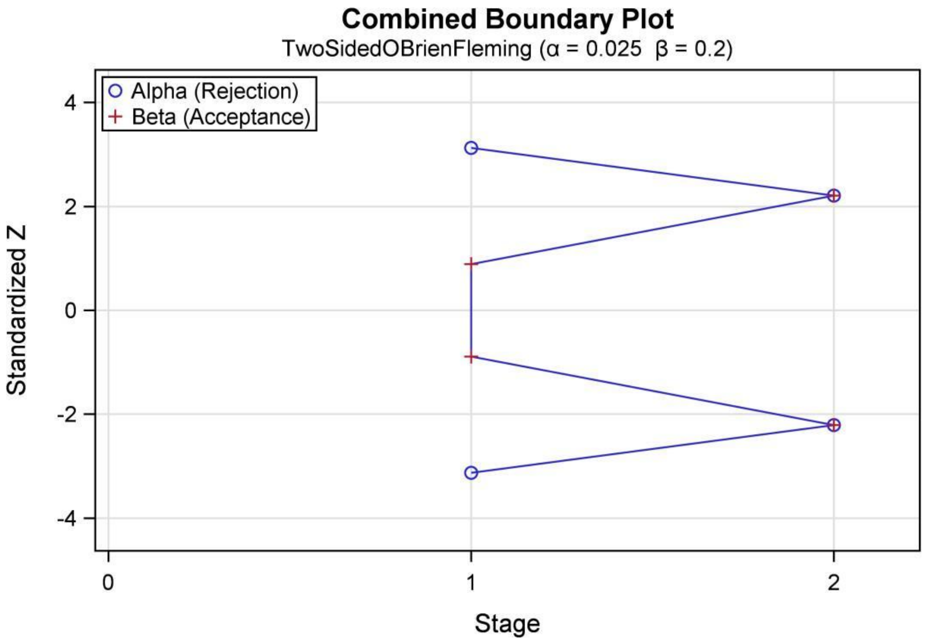

Table 11. CS #2003: Two-sided O'Brien-Fleming Boundaries for Rejection (α) and Acceptance (β) – SCI-QOL Physical Medical Health (PMH) domain

Interim Look (Stage)	No. of Randomized Participants	Std. z (Rejection)		Std. z (Acceptance)		Type-I error
		Lower	Upper	Lower	Upper	
1	80	-3.16956	3.16956	-0.40071	0.40071	0.00076
2	160	-2.24122	2.24122	-2.24122	2.24122	0.01250

Figure 3. CS #2003 Combined Boundary Plot for SCI-QOL Physical Medical Health (PMH) Domain

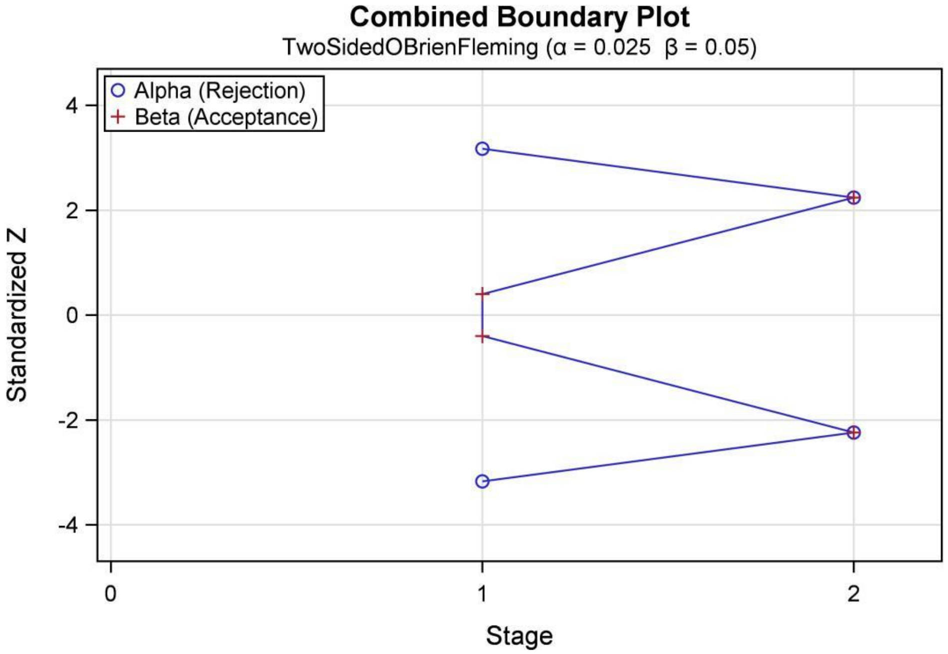

As with any clinical trial, the safety of the participants will be of utmost concern. Safety will be monitored closely, and data will be collected on adverse events (AEs) and serious adverse events (SAEs) throughout the course of the study. All AEs and SAEs will be systematically recorded on case report forms and coded by the CSP Clinical Research Pharmacy Coordinating Center (CSPCRPCC) at Albuquerque using the MedDRA system. The MedDRA system employs standard terminology to present adverse events and to organize them by body system. The incidence of adverse events will be summarized for each treatment group and overall, by body system and MedDRA preferred term. The incidence differences between the control and

intervention groups for each event will be tested using the Pearson chi-square test or Fisher's Exact test. Table 12 illustrates how AE data may be presented for this study.

Table 12. CS #2003 Cumulative Incidence of Adverse Events by Body System and MedDRA Term

	SOC (n = ?)	EAW + SOC (n = ?)	All Participants (N = ?)	
Body System and Preferred Term	n (%)	n (%)	n (%)	p-value
All Adverse Events				
Subjects with at least one Adverse Event				
Body System 1				
Event 1				
Event 2				
Event 3				
.				
.				
.				
Body System 2				
Event 7				
Event 8				
Event 9				
Event 10				
.				
.				
.				
Body System N				
Event 14				
Event 15				
Event 16				
.				
.				
.				
Event N				

It is the responsibility of the CSPCC Study Team to provide the DMC with whatever information they feel they need to successfully monitor the study. Thus, additional tables will be added as required. In addition to the reports for the yearly meetings, the DMC will also be provided with reports between meetings at 6-month intervals.

CSP #2003 (Exoskeletal-Assisted Walking in Persons with SCI: Impact on Quality of Life)

Interim Analysis

March 5, 2019

As specified in the Statistical Analysis Plan (Section XIII) of the protocol, one interim look at the primary and major secondary outcome measures (in addition to the final "look" at study's end) was proposed to occur when 80, or 50%, of patients had completed their participation in the study. As of March 5, 2019, 86 participants had completed the study. Thus, we have performed the one interim look of the primary and major secondary outcome measures, success or failure at achieving the following (change from baseline to the end of the 4-month intervention phase):

- ≥ 4-point Improvement in the VR-36 Mental Component Summary (primary)
- ≥ 10% Improvement in the Sum T-Score of the SCI-QOL Physical Medical Health (PMH) Domain (primary)
- ≥ 1 kg. Total Body Fat Loss (major secondary)

VR-36 Mental Component Summary (primary outcome)

Table III-6a provides the frequencies of success and failure at achieving at least a 4-point improvement in the VR-36 Mental Component Summary (MCS) from baseline to the end of the 4-month Intervention phase of the study. As the table shows, the EAW and SOC groups had 8 successes each. The chi-square test did not find a significant difference between the groups (p=0.918). Figure III-1 provides the two-sided O'Brien-Fleming boundaries for rejection and acceptance regions for the two looks (interim and final). At this interim look, the standardized Z-score corresponding to the chi-square test was 0.103. As the figure illustrates, this point falls in the acceptance region. Table III-6b shows the frequency table for those participants that fully completed the study (no early terminators). The chi-square test again showed no difference between the groups (p=0.360). Descriptive statistics for the VR-36 MCS by time point and treatment group are provided in Table III-6c while Table III-6d gives statistics on the change from baseline to each of the time points at which the outcome was assessed. As a secondary analysis, differences between the groups were assessed at each time point using a paired t-test.

SCI-QOL Physical Medical Health (PMH) Domain (primary outcome)

Table III-7a provides the frequencies of success and failure at achieving at least a 10% improvement in the SCI-QOL Physical Medical Health Domain (bladder management difficulties, bowel management difficulties and pain interference item banks) sum T-score from baseline to the end of the 4-month Intervention phase of the study. As the table shows, of the 42 participants in the EAW group, 5 were successes (11.9%) while 8 of 44 (18.2%) in the SOC group were successful at achieving at least a 10% improvement in the measure. The chi-square test did not find a significant difference between the groups (p=0.417). Figure III-2 provides the two-sided O'Brien-Fleming boundaries for rejection and acceptance regions for the two looks (interim and final). At this interim look, the standardized Z-score corresponding to the chi-square test was 0.812. As the figure illustrates, this point falls in the "no decision" zone of the graph. Table III-7b shows the frequency table for those participants that fully completed the

study (no early terminators). The chi-square test again shows no difference between the groups (p=0.878). Descriptive statistics for the SCI-QOL PMH by time point and treatment group are provided in Table III-7c while Table III-7d gives statistics on the change from baseline to each of the time points at which the outcome was assessed. As a secondary analysis, differences between the groups were assessed at each time point using a paired t-test.

Total Body Fat (major secondary outcome)

Table III-8a provides the frequencies of success and failure at achieving at least a 1 kg. loss in total body fat from baseline to the end of the 4-month Intervention phase of the study. As the table shows, of the 42 participants in the EAW group, 6 were successes (14.3%) while 10 of 44 (22.7%) in the SOC group were successful at achieving at least a 1 kg. loss in total body fat. The chi-square test did not find a significant difference between the groups (p=0.315). Figure III-3 provides the two-sided O'Brien-Fleming boundaries for rejection and acceptance regions for the two looks (interim and final). At this interim look, the standardized Z-score corresponding to the chi-square test was 1.006. As the figure illustrates, this point falls in the "no decision" zone of the graph. Table III-8b shows the frequency table for those participants that fully completed the study (no early terminators). The chi-square test again shows no difference between the groups (p=0.789). Descriptive statistics for total body fat by time point and treatment group are provided in Table III-8c while Table III-8d gives statistics on the change from baseline to each of the time points at which the outcome was assessed. As a secondary analysis, differences between the groups were assessed at each time point using a paired t-test.

PRIVILEGED AND CONFIDENTIAL
Not to be Disseminated Beyond Official Use

CSP 2003 Exoskeletal-Assisted Walking in Persons with SCI: Impact on Quality of Life
Table III-6a Veterans RAND 36 Item Health Survey: Improvement in the Mental Component Summary (MCS) from Baseline to End of 4 Month Intervention, by Treatment Group

≥4 Point Improvement	EAW (n= 42) n (%)	SOC (n= 44) n (%)	Total (n= 86) n (%)	P-value
**Success**	8 (19.0)	8 (18.2)	16 (18.6)	0.918
**Failure**	34 (81.0)	36 (81.8)	70 (81.4)	
**Total**	**42 (48.8)**	**44 (51.2)**	**86 (100.0)**	

SAS program: P2003-J16_J19-VR36-V1 Calculated: 05APR19 15:15 (Data update: 05MAR2019)
T:\P2003\Reports\DMC\DMC-2019-03-05\Tables\interim analysis\VR36_PrimaryOutcome-05APR19.doc

Figure III-1 Two-sided O'Brien-Fleming Boundaries for Rejection and Acceptance Regions for MCS/VR36

The SEQTEST Procedure

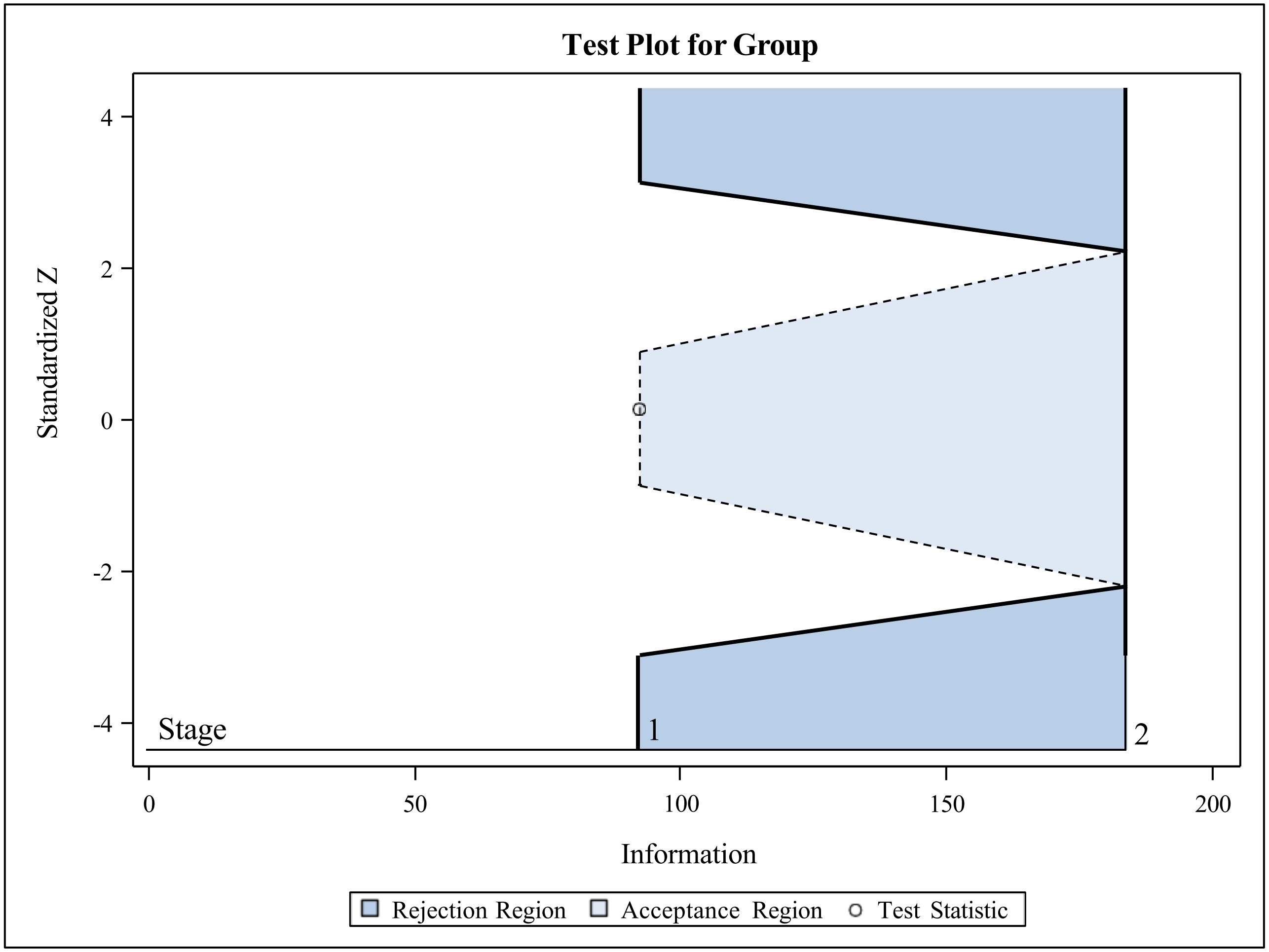

CSP 2003 Exoskeletal-Assisted Walking in Persons with SCI: Impact on Quality of Life
Table III-6b Veterans RAND 36 Item Health Survey: Improvement in the Mental Component Summary (MCS) from Baseline to End of 4 Month Intervention, by Treatment Group, in Participants Who Completed the 4 Month Intervention Phase

	EAW (n= 25)	SOC (n= 37)	Total (n= 62)	
≥4 Point Improvement	n (%)	n (%)	n (%)	P-value
**Success**	8 (32.0)	8 (21.6)	16 (25.8)	0.360
**Failure**	17 (68.0)	29 (78.4)	46 (74.2)	
**Total**	**25 (40.3)**	**37 (59.7)**	**62 (100.0)**	

CSP 2003 Exoskeletal-Assisted Walking in Persons with SCI: Impact on Quality of Life
Table III-6c Veterans RAND 36 Item Health Survey: Mental Component Summary (MCS) by Time Point and Treatment Group

	EAW (n= 53)						SOC (n= 50)					
Time Point	N	Mean	SD	Median	Min	Max	N	Mean	SD	Median	Min	Max
**Baseline**	52	61.1	11.17	65.8	28.3	72.1	48	58.4	11.13	62.5	23.9	74.2
**Post-Training/Orientation**	31	64.8	7.26	67.0	37.4	73.8	37	58.3	10.32	62.3	29.9	70.3
**Two Months into Intervention Phase**	28	63.5	9.30	67.0	29.8	74.7	36	58.3	12.42	64.1	25.6	75.6
**Post-Intervention Phase**	25	63.3	9.89	64.9	30.3	76.7	37	57.2	12.94	61.1	26.4	72.8

CSP 2003 Exoskeletal-Assisted Walking in Persons with SCI: Impact on Quality of Life
Table III-6d Veterans RAND 36 Item Health Survey: Change in Mental Component Summary (MCS) from Baseline, by Treatment Group

	EAW (n= 53)						SOC (n= 50)						
Time Point	N	Mean	SD	Median	Min	Max	N	Mean	SD	Median	Min	Max	P-value
Post-Training/Orientation	31	1.5	11.69	0.5	-28	32.7	35	-0.2	6.29	0.0	-14	15.8	0.468
Two Months into Intervention Phase	28	0.6	8.40	1.1	-18	26.4	34	0.4	7.57	0.7	-21	14.1	0.948
Post-Intervention Phase	25	0.8	10.54	1.0	-27	29.3	35	-1.5	9.61	1.5	-34	16.1	0.375

CSP 2003 Exoskeletal-Assisted Walking in Persons with SCI: Impact on Quality of Life
Table III-7a SCI-QOL Physical Medical Health Domain: Improvement in the Sum T-Score from Baseline to End of 4 Month Intervention, by Treatment Group

	EAW (N= 42)	SOC (N= 44)	Total (N= 86)	
≥10 Percent Improvement	N (%)	N (%)	N (%)	P-value
Success	5 (11.9)	8 (18.2)	13 (15.1)	0.417
Failure	37 (88.1)	36 (81.8)	73 (84.9)	
**Total**	**42 (48.8)**	**44 (51.2)**	**86 (100.0)**	

Figure III-2 Two-sided O'Brien-Fleming Boundaries for Rejection and Acceptance Regions for SCI-QOL Physical Medical Health Domain

The SEQTEST Procedure

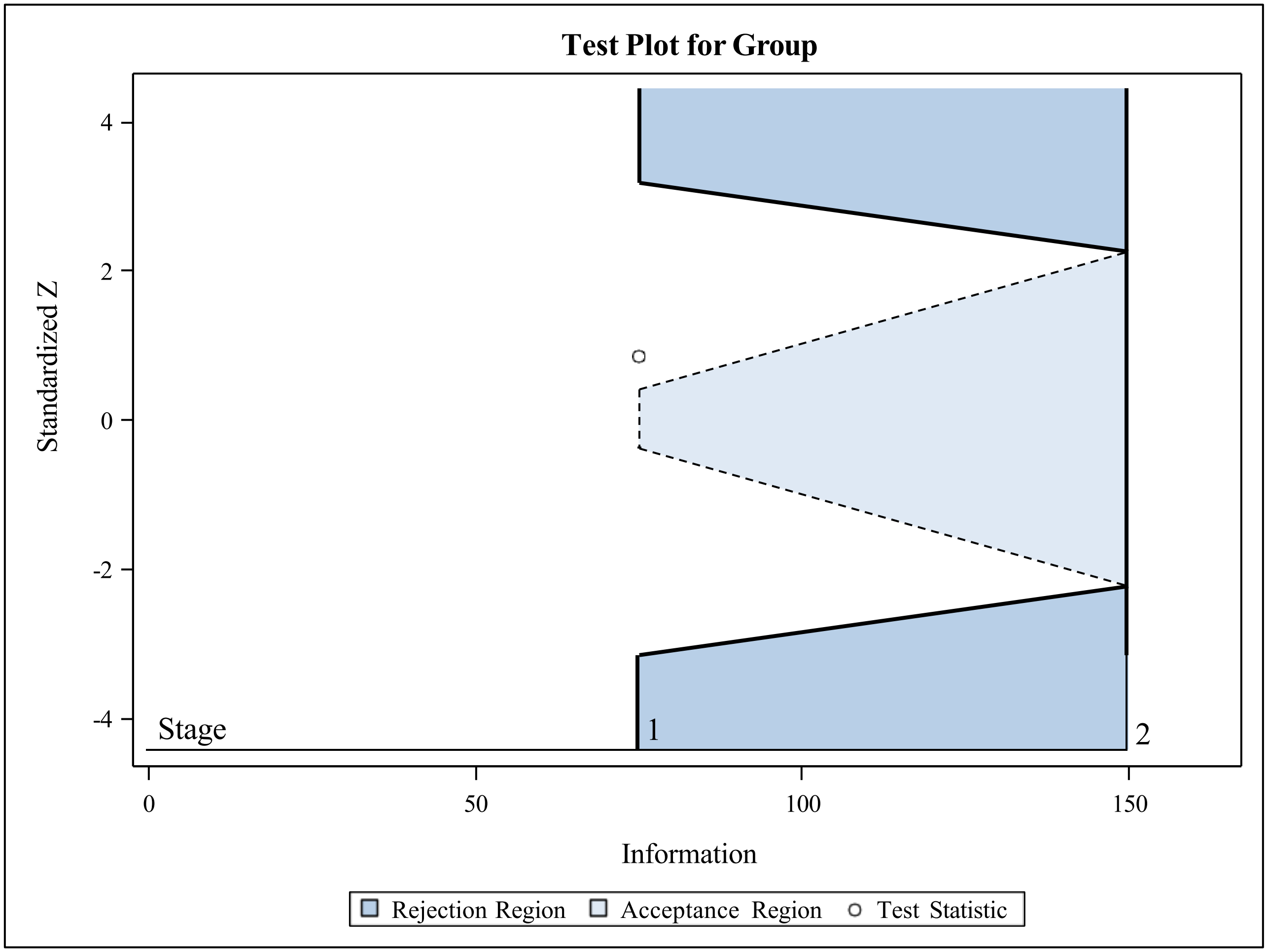

CSP 2003 Exoskeletal-Assisted Walking in Persons with SCI: Impact on Quality of Life
Table III-7b SCI-QOL Physical Medical Health Domain: Improvement in the Sum T-Score from Baseline to End of 4 Month Intervention, by Treatment Group, in Participants Who Completed the 4 Month Intervention Phase

	EAW (N= 25)	SOC (N= 37)	Total (N= 62)	
**≥10 Percent Improvement**	**n (%)**	**n (%)**	**n (%)**	**P-value**
**Success**	5 (20.0)	8 (21.6)	13 (21.0)	0.878
**Failure**	20 (80.0)	29 (78.4)	49 (79.0)	
**Total**	**25 (40.3)**	**37 (59.7)**	**62 (100.0)**	

CSP 2003 Exoskeletal-Assisted Walking in Persons with SCI: Impact on Quality of Life
Table III-7c SCI-QOL Physical Medical Health Domain: Sum T-Score by Time Point and Treatment Group

	EAW (N= 53)						SOC (N= 50)					
**Time Point**	**N**	**Mean**	**SD**	**Median**	**Min**	**Max**	**N**	**Mean**	**SD**	**Median**	**Min**	**Max**
**Baseline**	48	151	22.88	149	110	217	47	155	23.08	156	110	198
**Post-Training/Orientation**	29	146	15.99	145	115	174	38	151	25.68	153	110	201
**Two Months into Intervention Phase**	25	147	20.77	154	110	179	33	153	23.31	155	118	195
**Post-Intervention Phase**	23	143	19.71	143	110	175	36	148	26.76	141	110	206

CSP 2003 Exoskeletal-Assisted Walking in Persons with SCI: Impact on Quality of Life
Table III-7d SCI-QOL Physical Medical Health Domain: Change in Sum T-Score from Baseline by Treatment Group

	EAW (N= 53)						SOC (N= 50)						
**Time Point**	**N**	**Mean**	**SD**	**Median**	**Min**	**Max**	**N**	**Mean**	**SD**	**Median**	**Min**	**Max**	**P-value**
**Post-Training/Orientation**	29	-3.2	20.49	-2.7	-46	39.8	37	-2.2	16.35	-2.9	-32	31.4	0.828
**Two Months into Intervention Phase**	23	-4.1	18.10	0.0	-47	26.5	31	-2.7	15.81	-2.6	-33	29.6	0.771
**Post-Intervention Phase**	21	-7.6	21.89	-4.4	-62	22.1	35	-3.4	19.94	-2.9	-44	32.4	0.470

CSP 2003 Exoskeletal-Assisted Walking in Persons with SCI: Impact on Quality of Life
Table III-8a Total Body Fat Loss from Baseline to End of 4 Month Intervention by Treatment Group

	EAW (n= 42)	SOC (n= 44)	Total (n= 86)	
**≥1 KG loss**	**n (%)**	**n (%)**	**n (%)**	**P-value**
**Success**	6 (14.3)	10 (22.7)	16 (18.6)	0.315
**Failure**	36 (85.7)	34 (77.3)	70 (81.4)	
**Total**	**42 (48.8)**	**44 (51.2)**	**86 (100.0)**	

Figure III-3 Two-sided O'Brien-Fleming Boundaries for Rejection and Acceptance Regions for Total Body Fat Mass

The SEQTEST Procedure

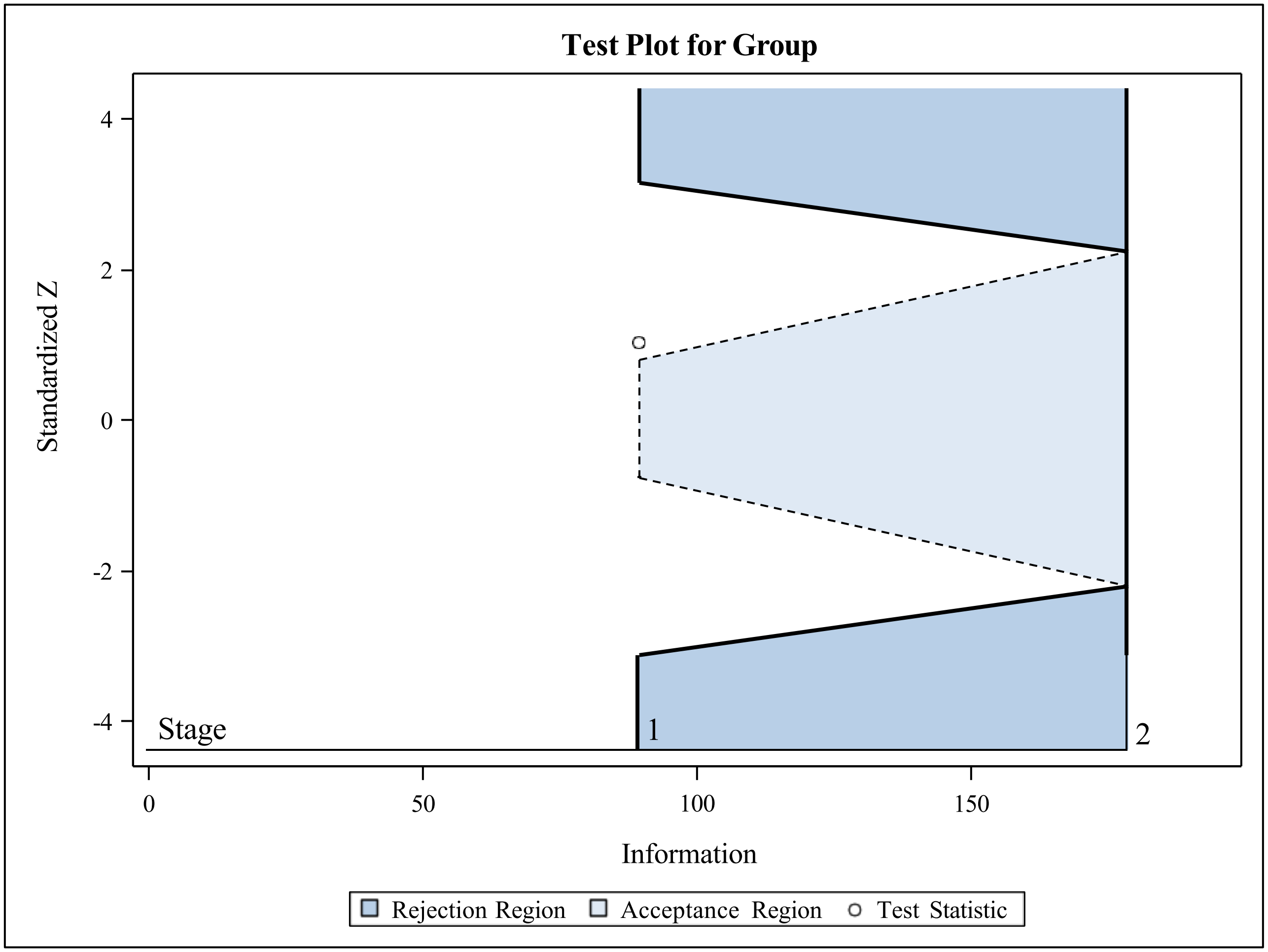

CSP 2003 Exoskeletal-Assisted Walking in Persons with SCI: Impact on Quality of Life
Table III-8b Total Body Fat Loss from Baseline to End of 4 Month Intervention, by Treatment Group, in Participants Who Completed the 4 Month Intervention Phase

≥1 KG loss	EAW (n= 25) n (%)	SOC (n= 37) n (%)	Total (n= 62) n (%)	P-value
Success	6 (24.0)	10 (27.0)	16 (25.8)	0.789
Failure	19 (76.0)	27 (73.0)	46 (74.2)	
**Total**	**25 (40.3)**	**37 (59.7)**	**62 (100.0)**	

CSP 2003 Exoskeletal-Assisted Walking in Persons with SCI: Impact on Quality of Life
Table III-8c Total Body Fat, in Kilograms, by Time Point and Treatment Group

	EAW (n= 53)						SOC (n= 50)					
Time Point	N	Mean	SD	Median	Min	Max	N	Mean	SD	Median	Min	Max
Baseline	53	26.9	9.35	27.9	8.3	47.7	50	26.6	8.44	26.8	10.4	44.0
Post-Training/Orientation	31	28.4	10.24	31.1	9.4	46.1	36	26.5	8.79	26.4	10.6	43.9
Two Months into Intervention Phase	27	28.7	10.59	28.7	9.0	46.6	34	26.1	9.23	26.6	2.9	39.1
Post-Intervention Phase	25	28.9	10.63	28.9	9.0	46.9	36	26.6	7.49	27.0	10.1	39.4

CSP 2003 Exoskeletal-Assisted Walking in Persons with SCI: Impact on Quality of Life
Table III-8d Change in Total Body Fat, in Kilograms, from Baseline by Treatment Group

	EAW (n= 53)						SOC (n= 50)						
Time Point	N	Mean	SD	Median	Min	Max	N	Mean	SD	Median	Min	Max	P-value
Post-Training/Orientation	31	0.3	1.83	0.6	-3.7	5.4	36	0.0	1.86	-0.4	-5.0	4.0	0.584
Two Months into Intervention Phase	27	0.7	2.84	0.9	-5.7	7.2	34	-1.1	5.66	-0.3	-28	6.9	0.110
Post-Intervention Phase	25	0.3	3.47	0.7	-11	6.1	36	-0.1	3.80	-0.3	-12	7.9	0.682
