# Supplementary material for: Exoskeletal-Assisted Walking in Veterans With Paralysis: A Randomized Clinical Trial
Source: JAMA Netw Open. 2024 Sep 4;7(9):e2431501. doi: 10.1001/jamanetworkopen.2024.31501 (PMC11375472; doi:10.1001/jamanetworkopen.2024.31501)
Supplement: Supplement 3. — Data Sharing Statement [file jamanetwopen-e2431501-s003.pdf]

Data Sharing Statement

Spungen. Exoskeletal-Assisted Walking in Veterans With Paralysis. *JAMA Netw Open*. Published September 04, 2024. doi:10.1001/jamanetworkopen.2024.31501

Data
Data available: Yes
Data types: Deidentified participant data, Data dictionary
How to access data: Please direct inquiries to: Dr. Kousick Biswas, PhD Health Science Officer Director, CSP Coordinating Center, Perry Point VA Medical Center VA Maryland Health Care System, Building 362T, Room 101 Perry Point, MD 21902 kousick.biswas@va.gov 410-642-2411, x25283
When available: With publication

Supporting Documents
Document types: None

Additional Information
Who can access the data: Per Department of Veterans Affairs Office of Research & Development Data Management and Access Plan (DMAP), Final data sets that are deidentified resulting from this research will be shared upon written request. Individually identifiable private information, protected health information (PHI), and other VA sensitive data will not be shared.
Types of analyses: Per Department of Veterans Affairs Office of Research & Development Data Management and Access Plan (DMAP), Final data sets that are de-identified resulting from this research will be shared upon written request. Individually identifiable private information, protected health information (PHI), and other VA sensitive data will not be shared.
Mechanisms of data availability: Per Department of Veterans Affairs Office of Research & Development Data Management and Access Plan (DMAP), Final data sets that are de-identified resulting from this research will be shared upon written request. Individually identifiable private information, protected health information (PHI), and other VA sensitive data will not be shared.
Any additional restrictions: Per Department of Veterans Affairs Office of Research & Development Data Management and Access Plan (DMAP), Final data sets that are de-identified resulting from this research will be shared upon written request. Individually identifiable private information, protected health information (PHI), and other VA sensitive data will not be shared.
